# *De Novo* Characterization of the Mung Bean Transcriptome and Transcriptomic Analysis of Adventitious Rooting in Seedlings Using RNA-Seq

**DOI:** 10.1371/journal.pone.0132969

**Published:** 2015-07-15

**Authors:** Shi-Weng Li, Rui-Fang Shi, Yan Leng

**Affiliations:** School of Chemical and Biological Engineering, Key Laboratory of Extreme Environmental Microbial Resources and Engineering Gansu Province, Lanzhou Jiaotong University, 88 West Anning Road, Lanzhou, 730070, P.R. China; Cankiri Karatekin University, TURKEY

## Abstract

Adventitious rooting is the most important mechanism underlying vegetative propagation and an important strategy for plant propagation under environmental stress. The present study was conducted to obtain transcriptomic data and examine gene expression using RNA-Seq and bioinformatics analysis, thereby providing a foundation for understanding the molecular mechanisms controlling adventitious rooting. Three cDNA libraries constructed from mRNA samples from mung bean hypocotyls during adventitious rooting were sequenced. These three samples generated a total of 73 million, 60 million, and 59 million 100-bp reads, respectively. These reads were assembled into 78,697 unigenes with an average length of 832 bp, totaling 65 Mb. The unigenes were aligned against six public protein databases, and 29,029 unigenes (36.77%) were annotated using BLASTx. Among them, 28,225 (35.75%) and 28,119 (35.62%) unigenes had homologs in the TrEMBL and NCBI non-redundant (Nr) databases, respectively. Of these unigenes, 21,140 were assigned to gene ontology classes, and a total of 11,990 unigenes were classified into 25 KOG functional categories. A total of 7,357 unigenes were annotated to 4,524 KOs, and 4,651 unigenes were mapped onto 342 KEGG pathways using BLAST comparison against the KEGG database. A total of 11,717 unigenes were differentially expressed (fold change>2) during the root induction stage, with 8,772 unigenes down-regulated and 2,945 unigenes up-regulated. A total of 12,737 unigenes were differentially expressed during the root initiation stage, with 9,303 unigenes down-regulated and 3,434 unigenes up-regulated. A total of 5,334 unigenes were differentially expressed between the root induction and initiation stage, with 2,167 unigenes down-regulated and 3,167 unigenes up-regulated. qRT-PCR validation of the 39 genes with known functions indicated a strong correlation (92.3%) with the RNA-Seq data. The GO enrichment, pathway mapping, and gene expression profiles reveal molecular traits for root induction and initiation. This study provides a platform for functional genomic research with this species.

## Introduction

Adventitious roots refer to roots that form from any tissue that is not a root, such as leaves and stems. Adventitious rooting is one of the most important mechanisms of vegetative propagation in plants and one of the most important methods for the commercial production of horticultural species throughout the world [1]. As an alternative or supplement to seed propagation in ecosystems where soil disturbances occur frequently, adventitious rooting is an important plant response to environmental stresses and a strategy for plant propagation under stress [2]. The formation of adventitious roots has been associated with an important aspect of tissue dedifferentiation that involves shifting cells from normal morphogenetic pathways to functions associated with the development of root primordia [3]. This shift leads to *de novo* root formation and a multitude of metabolic changes involving the enzymes and macromolecules associated with the induction, initiation, and development of root primordia in plant cuttings [4]. Although the physiological and biochemical changes that occur during adventitious root formation have been extensively studied, the molecular mechanisms involved remain less well understood.

Various molecular and genetic approaches have been used to study adventitious root development in *Arabidopsis* and other plants [5]. The physiological and biochemical changes that occur during the complex process of *in vitro* root development must be attributed to the presence and activity of metabolic pathways. In turn, these metabolic pathways must be controlled by the regulation of RNA transcription. Identifying the RNA transcription profile during this process will thus improve our understanding of the fundamental processes that control adventitious rooting. To this end, several studies have sought to investigate the transcriptional changes and differences in gene expression that occur during adventitious root formation using proteomic and cDNA microarrays [6–10]. Using the latter method, Brinker et al. (2004) identified 220 genes that were changed significantly during root development in hypocotyl cuttings of *Pinus contorta* [6]. Proteomic analyses were also used to investigate the proteins involved in the adventitious rooting of *Arabidopsis thaliana* mutants by Sorin et al. (2006), who identified 11 proteins predicted to be involved in different biological processes, including the regulation of auxin homeostasis and light-associated metabolic pathways [7]. Using the Medicago GeneChip, Holmes et al. (2010) identified 904 and 993 up- and down-regulated probe sets in root-forming cultures of *Medicago truncatula* as well as significant changes in metabolism, signaling and the expression of transcription factors linked to *in vitro* adventitious root formation processes [8]. Recently, using a NimbleGen microarray, Rigal et al. (2012) identified 7,107 transcript levels that changed during early stages of adventitious root development in the model tree *Populus trichocarpa* [9].

A major limitation of the microarray method is that only a portion of the total transcripts can be assayed. Many genes are not represented on the microarrays, while genes from large and highly similar families may yield ambiguous expression results due to non-specific hybridization [11]. Recently, a high-throughput deep-sequencing technology (i.e., next-generation sequencing, NGS), RNA-Seq, has been widely used to explore transcriptomic data and study gene expression at the whole genome level in model and non-model organisms [12, 13]. Emerging *de novo* short read assembly technology has been successfully applied to identify gene expression profiles and discover new genes without a reference genome sequence [13, 14]. This technology platform enables the precise elucidation of transcripts present within a particular sample and can be used to calculate gene expression based on absolute transcript abundance [15].

The process of adventitious rooting consists of three successive but interdependent physiological stages, namely, induction, initiation and expression. The induction stage comprises molecular and biochemical events without visible changes. The initiation stage is characterized by cell division and organization of the root primordia [1, 16]. Studies in herbaceous plants reveal that the critical events that culminate in the formation of adventitious roots in hypocotyl cuttings occur within the first 3–12 h after excision of the primary roots [3, 17]. In mung bean hypocotyl cuttings, the induction stage lasts from 0 h to 12 h after primary root excision, and the initiation stage lasts from 12 h to 48 h. The first emerging adventitious root primordia were clearly visible at 48 h and adventitious roots grown through the epidermis of the hypocotyls within 72 h of the start of the cutting cultures [16, 18]. In cuttings of woody plants such as in *Malus* and *Populus*, cell divisions as early as 48 h after auxin exposure [17, 19]. Early significant physiological and biochemical changes in endogenous hormone pools occur during the first 48 h after excision [20]. Transcriptome monitoring in *Populus trichocarpa* cuttings revealed significant shifts during 0–48 h time period after excision. 27% of the genes were differentially regulated between 0 and 6 h, 36% between 6 and 24 h, and 4% between 24 and 48 h [2,19]. The critical dedifferentiation events during the process of adventitious rooting occur within these two stages.

Herein, we exploited RNA-Seq technology to characterize the mung bean transcriptome and further to highlight global changes in gene expression during early stage of root development (i.e., induction and initiation stages) in mung bean hypocotyl cuttings. Mung bean is one of the most important tropical grain legumes that serves as a significant and a cheap source of carbohydrates and easily digestible protein for the people of Asia and Africa, but increasingly extends into Australia, USA, Canada and Ethiopia [21]. However, the genomic and transcriptomic data of this plant have not been revealed so far. Furthermore, this plant has been widely used as a model plant species for studying physiological, biochemical, and molecular mechanisms under the process of adventitious root formation [16, 18, 22–25]. In present study, we aimed to characterize the molecular basis of physiological processes that occur during early stage of root development and to identify differentially expressed genes (DEGs) and metabolic pathways. Real-time quantitative PCR was used to validate several of the transcriptional changes observed.

## Materials and Methods

### Plant material and culture conditions

**Fig 1:**
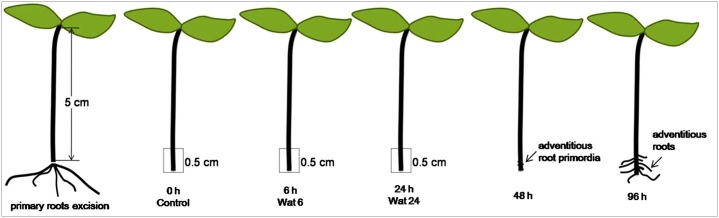
Time course of adventitious root development in mung bean hypocotyls after the primary roots excision. Adventitious root primordia are visible at 48 h after the primary roots excision and adventitious roots grow through the epidermis of the hypocotyls within 96 h. The basal 0.5 cm of hypocotyls at 0 h (Con), 6 h (Wat6), and 24 h (Wat24) after the primary roots excision and incubation in water were harvested and used as study samples.

Mung bean [*Vigna radiata* (L.) R. Wilczek] seeds were washed in distilled water and sterilized in a 6% sodium hypochlorite solution for 15 min. The seeds were subsequently washed three times in sterile distilled water, sown in Petri dishes (30 seeds per a 12 cm- Petri dishes), covered with a 5 mm-layer of sterilized perlite, and incubated in a growth chamber at 25±1°C for 36 h in the dark and then at 25±1°C with a 14-h photoperiod under white fluorescent lamps (PAR of 100 μM m^−2^ s^−1^). Five days after germination, seedlings that were 5 cm in height were used for the experiments. To investigate gene expression changes during adventitious rooting, the primary roots of the seedlings were removed from the bases of the hypocotyls, and the resulting explants (10 per beaker) were cultured in 50-mL beakers containing 40 mL sterilized distilled water for 6 h (Wat6) or 24 h (Wat24) under the same aseptic conditions applied to the seedling culture. The basal 0.5 cm of each hypocotyl, where adventitious roots originated *in vitro*, was cut and harvested after a 6- or 24-h incubation. The same parts of seedling hypocotyls were directly harvested and used as the control tissues (Con) (Fig 1). The three parallel treatments were set in each group. All of the harvested tissues were immediately frozen in liquid nitrogen and stored at -80°C until further analysis.

### Total RNA extraction

The tissues from 10 hypocotyls were fully ground in liquid nitrogen, and approximately 50 mg of tissue powder was mixed with 600 μL buffer Rlysis-P (from kit SK8631, Sangon, Shanghai, China) in a 1.5 mL RNase-free tube for 5 min in a water bath at 65°C to ensure sufficient lysis. Next, 60 μL buffer PCA (from kit SK8631, Sangon) was added and mixed thoroughly, and the mixture was incubated at -20°C for 3 min. After centrifugation at 10,000 g for 5 min at 4°C, an equal volume of cooled phenol chloroform (phenol water) was added to the supernatant, mixed, and then centrifuged at 12,000 g for 5 min at 4°C. An equal volume of cooled chloroform was added to the supernatant and mixed. Following centrifugation at 12,000 g for 5 min at 4°C, an equal volume of cooled isopropanol was added to the supernatant, shaken gently, and left to precipitate for 10 min. After centrifugation at 12,000 g for 20 min at 4°C, the pellet was recovered, washed twice with 75% ethanol, dried for 5–15 min at ambient temperature, dissolved in 50 μL RNase-free water, and stored at -80°C. A 2100 Bioanalyzer (Agilent Technologies, Santa Clara, CA, USA) was used to confirm RNA integrity with RNA Integrity Number (RIN) values of 8.1–9.9. RNA concentration was determined using a NanoDrop ND-1000 Spectrophotometer (NanoDrop, Wilmington, DE, USA).

### cDNA library construction and transcriptome sequencing

Equal amounts of total RNA from each sample were pooled to construct the cDNA library. Oligo(dT) 25 beads (Invitrogen) were used to enrich for poly(A) mRNAs from the total RNA pool. Following purification, the mRNA was cleaved into fragments using Fragment Mix reactive system at 94°C for 4 min. First-strand cDNA was synthesized using Superscript II reverse transcriptase (18064–014, Invitrogen), First Strand Master Mix, random hexamer (N6) primers, and the fragmented mRNA templates. The reaction was performed at 25°C for 10 min, 42°C for 50 min, 70°C for 15 min, and then held at 4°C. Subsequently, the second strand cDNA was synthesized using Second Strand Master Mix (18064–014, Invitrogen). The synthesized dscDNA fragments were purified with Agencourt AMPure XP Beads (Agencourt). The End Repair Control and AMPure XP beads were used to repair the 3' ends and purify the repaired cDNA fragments. Subsequently, adenylation of the 3' ends of the cDNA fragments was conducted using Klenow exo (M0212L, NEB). After end repair and A-tailing, Illumina paired-end adapters were ligated to the cDNA fragments using T4 Ligase (Fermentas) and purified twice with AMPure XP Beads. To prepare the cDNA sequencing library, the ligated cDNA was enriched and amplified using selective PCR. The PCR procedure was performed as follows: 98°C for 30 s; 15 cycles of 98°C for 10 s, 60°C for 30 s, 72°C for 30 s, and 72°C for 5 min; holding at 4°C, followed by purification with AMPure XP beads. The quality and quantity of the cDNA library were measured using the Agilent 2100 Bioanalyzer and Qubit 2.0 (Life Technologies). Finally, paired-end sequencing of the constructed cDNA library was carried out at Sangon Biotech. Co. Ltd. (Shanghai, China) on an Illumina HiSeq 2000 system (Illumina).

### *De novo* assembly and sequence clustering

The raw reads were filtered, and high-quality clean read data were obtained by deleting adaptor sequences, removing reads containing more than 5% ambiguous bases (undetermined bases, N) and low-quality reads (reads containing more than 10% bases with a Q-value ≤20). The *de novo* assembly of the clean reads was carried out using the TRINITY paired-end assembly method (Trinity RNA-Seq r2013-02-25, http://trinityrnaseq.sourceforge.net/) [26] with an optimized k-mer length of 25. The assembled sequences were clustered with Chrysalis, a module of Trinity. The longest transcript that could not be extended on either end within each clustered loci was defined as a unigene. The assembled unigenes (longer than 200 bp) have been deposited in the Transcriptome Shotgun Assembly Sequence Database (http://www.ncbi.nih.gov/genbank/tsa.html) at DDBJ/EMBL/GenBank under the accession number GBXO01000001-GBXO01078617.

Similarity searches were performed using locally installed BLAST+ v2.2.27 software [27]. The transcripts and unigenes were subjected to similarity searches against protein and nucleotide sequence databases using BLASTx and MEGABLAST, respectively, at an e-value cut-off of e-5. BLAST annotations were filtered using either subject or query coverage (>30%) and sequence identity (>50% for megablast and >30% for blastx).

### Mapping reads, calling variations and quantifying transcripts

Due to the lack of a reference sequence, the assembled transcripts were assumed to be the reference sequence to compute transcript expression levels [26, 28, 29]. The expression values were used to create an expression profile with the help of Agilent's GeneSpring program. The read sequences were aligned against these transcript reference sequences using BWA-0.6.2-http://bio-bwa.sourceforge.net/ [30] in the end-to-end alignment mode.

### Functional annotation and classification

All resulting unigenes that exceeded 200 bp in length were annotated according to their sequence similarity to previously annotated genes. First, the unigenes were aligned using BLASTx to the public protein databases NR, SWISS-PROT, TrEMBL, Pfam, and CDD with similarity set at >30% and an E-value ≤1e-5. The KOG (Clusters of Orthologous Groups for eukaryotic complete genomes) and KEGG (Kyoto Encyclopedia of Genes and Genomes) pathway annotations were performed by sequence comparisons against the two databases using BLASTALL and KAAS software (ftp://ftp.ncbi.nih.gov/blast/executables/release/2.2.18/) with an E-value ≤1e-5. The resulting blast hits were processed using Blast2GO software (version 2.3.5, http://www.blast2go.de/) [31] with an E-value threshold of 1e-5 to retrieve associated GO terms. GO classification was achieved using WEGO software [32]. The results that presented the best alignment were used to identify the sequence direction and to predict the coding regions using BLASTx searches against protein databases, with the priority order of NR, SWISS-PROT, KEGG and KOG if conflicting results were obtained. The ESTScan software [33] was used to analyze the unigenes that did not align to any of the above databases. KEGG mapping was used to determine the metabolic pathways. Enzyme codes were extracted, and KEGG [34–36] pathways were retrieved from the KEGG web server (http://www.genome.jp/kegg/). To further enrich the pathway annotations, unigenes were submitted to the KEGG Automatic Annotation Server (KAAS) [37], and the single-directional best hit information method was selected. To identify the enriched pathways, the phyper test was used to measure the relative coverage of the annotated KEGG orthologous groups of a pathway against the transcriptome background, and the pathways with a p-value ≤0.05 were classified as enriched.

### Expression analysis and identification of DEGs

The expression levels of unigenes were measured by mapping back the number of clean reads to the assembled unigenes using BWA-0.6.2-http://bio-bwa.sourceforge.net/ [30]. The number of clean reads mapped to each unigene was calculated and then normalized to RPKM (reads per Kb per million reads) using ERANGE3.1 software [15]. Unigene expression levels were analyzed using the DEGseq R package [38] with the MARS (MA-plot-based method with Random Sampling) model. The DEGs between each pair of samples were screened using the Audic-Claverie algorithm [39] with an FDR threshold of ≤0.001 and an absolute value of log2 ≥1. Multiple test corrections of the p-value and FDR were performed with the Benjamini-Hochberg correction [40].

### Real-time quantitative reverse transcription PCR validation

To validate the transcriptome data, 39 genes with known functions that were assumed to play roles in adventitious root initiation were selected for further analysis. Hypocotyl tissues were harvested from three biological replicates subjected to the same experimental design as that of the samples subjected to Illumina sequencing for RNA-Seq. Total RNA was extracted with TRIzol reagent (Invitrogen, Carlsbad, CA, USA) and purified on RNeasy mini spin columns (Qiagen) with on-column DNase I treatment according to the manufacturer’s protocol. RNA integrity was examined with an Agilent Bioanalyzer 2100 (Agilent Technologies). First strand cDNA was synthesized using the AMV First Strand cDNA Synthesis Kit (Roche Applied Science, Mannheim, Germany) according to the manufacturer’s instructions. The gene-specific primer pairs (S8 Table) were designed using Primer Premier 5.0 software (Applied Biosystems, Foster City, CA, USA) according to the confirmed sequences. Real-time PCR was run in a LightCycler480 II (Roche Applied Science) with ABI SYBR Green PCR Master Mix (ABI, Foster, USA). The thermal cycling program was 95°C for 3 min and 40 cycles of 95°C for 15 s and 60°C for 40 s. Melting curve analysis was carried out for each primer set to verify the presence of a single melting peak after amplification. ‘No cDNA’ samples (water) and ‘no RT’ samples were included as negative controls. Output data were generated with Sequence Detector version 1.3.1 software (ABI) and evaluated using Student’s t-tests with the delta-delta Ct method described by Livak and Schmittgen [41]. The standard error of the mean was calculated for the three biological replicates. Expression levels were calculated relative to the reference gene using the comparative threshold cycle method.

## Results

### Solexa RNA paired-end sequencing

Total RNA extraction and cDNA synthesis were performed from three samples: hypocotyls (Con), hypocotyls after primary root excision and incubation in water for 6 h (Wat6, root induction stage), and hypocotyls after primary root removal and incubation in water for 24 h (Wat24, root initiation stage). The three cDNA libraries were sequenced separately using the Illumina HiSeq 2000 system and respectively generated 7.361e+09 bp, 5.998e+09 bp, and 5.885e+09 bp raw reads. Raw reads were subjected to quality control using SeqQC. The ratio of >Q20 bases was more than 87% across the three libraries. The percentages of undetermined bases (Ns) were 0.144%, 0.137%, and 0.224% in the three libraries, respectively (Table 1). After deleting adapter sequences and discarding low-quality sequences from the raw data, 6.832 Gbp (92.81% of the total reads), 5.558 Gbp (92.66% of the total reads), and 5.557 Gbp (94.42% of the total reads) of high-quality reads were obtained for the three libraries, respectively. The average length of the clean reads exceeded 95 bp, and the ratio of retained reads was more than 95% by pre-processing (Table 1). To assess the contamination of the processed reads, random sets of one hundred thousand sequences were aligned against the Nr database. The results are presented in S1 Table. This assay indicated that the sequencing quality was high enough for further analysis. These processed paired-end reads were used for transcript assembly.

**Table 1:** Sequencing data information.

	Con	Wat6	Wat24
Total reads	73613420	59983870	58854292
Total bases (bp)	7.361E+09	5.998 E+09	5.885E+09
Average read length (bp)	100	100	100
Q20 bases (bp)	6.418E+09	5.216 E+09	5.305E+09
Q20 bases ratio (%)	87.18	86.95	90.13
N bases (bp)	10592904	8243779	13161875
N bases ratio (%)	0.144	0.137	0.224
Clean bases	6.505E+09	5.285E+09	5.359E+09
Clean sequences	68319673	55579848	55569347
Clean ratio (%)	92.81	92.66	94.42
Clean mean length (bp)	95.22	95.09	96.44

### *De novo* assembly

The paired-end *de novo* assembly of the processed reads was performed using the TRINITY transcriptome assembly software program. After filtering out repetitive sequences and those shorter than 200 bases in length, a total of 133,287 transcripts (166 Mb) with a sequence length > 200 bp were generated. The total length of the transcripts was 1.66e+08 bases, and the mean length of the transcripts was approximately 1248 bases (Table 2). The average GC content of the transcripts was 37.84%, indicating that the transcripts were AT-rich at 62.16% (Table 3; S1 Fig). The N50 was 2132 in this assembly, which was higher than most other plant transcriptome assemblies [12, 26, 28, 42, 43]. The higher the N50 value, the better the assembly [12]. Further clustering using the Chrysalis cluster module of TRINITY resulted in 78,697 unigenes (65 Mb), which represented the longest transcripts in sequence length within each loci. Approximately 47% (37,438) of the unigenes had a length that exceeded 500 bp (Table 2; S2 Fig). It has been demonstrated that longer transcripts are easier and more likely to be mapped to correct transcript sequences [44]. The lengths of the assembled transcripts and unigenes are shown in S2 Fig. The ratios of mapped reads were 93.55%, 94.08%, and 94.04%, and the expression ratios of unigenes were 91.92% (72,342), 84.71% (66,663), and 82.19% (64,680) in the Con, Wat6, and Wat24 samples, respectively, demonstrating a decreasing trend in gene expression during root development (Table 3).

**Table 2:** Statistics of reads assembly with the Trinity method.

	Transcript	Unigene
Total number	133287	78697
Maximum length (in bases)	15660	15660
Minimum length (in bases)	201	201
Average length (in bases)	1248.7	832
Total length (in bases)	1.66E+08	6.568 E+08
> 500 b number	84251	37438
> 1 Kb number	57388	19706
N50 size (in bases)	2132	1403
GC %	37.84	36.94
AT %	62.16	63.06

**Table 3:** Sample mapping results and unigene abundance measurements in the samples.

	Con	Wat6	Wat24
Clean reads	68319673	55579848	55569347
Mapped reads	63915078	52288727	52255111
Mapped ratio	93.55%	94.08%	94.04%
All gene	78697	78697	78697
Expressed gene	72342	66663	64680
Expressed ratio	91.92%	84.71%	82.19%
RPKM≥1000 unigenes	49	51	55
RPKM 500–1000 unigenes	53	73	61
RPKM 100–500 unigenes	835	973	996
RPKM 10–100 unigenes	10126	8691	9322
RPKM 1–10 unigenes	19073	15643	14901
RPKM <1 unigenes	42454	41480	39593
Maxim RPKM	8080	22135	11141
Average RPKM	9.48	10.57	10.68

### Functional annotation of the unigenes

As a non-model plant, the mung bean unigenes obtained in this RNA-Seq analysis were aligned against the six public protein databases, Nr (NCBI non-redundant (nr) database), the SWISS-PROT protein database, TrEMBL, Pfam, KOG (Clusters of Orthologous Groups of proteins in eukaryotes), and CDD with the criteria of similarity >30% and E-value ≤1e-5. Approximately 36.77% of the unigenes (29,029) were annotated using BLASTx. Among them, 28,084 (35.69%), 27,934 (35.50%), 19434 (24.62%), 16704 (21.16%), 12738 (16.14%), and 11990 (15.19%) unigenes could be annotated using the TrEMBL, Nr, SWISS-PROT, CDD, Pfam, and KOG databases, respectively. A four-way Venn diagram was constructed to depict the shared sets of transcripts annotated by the four databases (S3 Fig).

**Fig 2:**
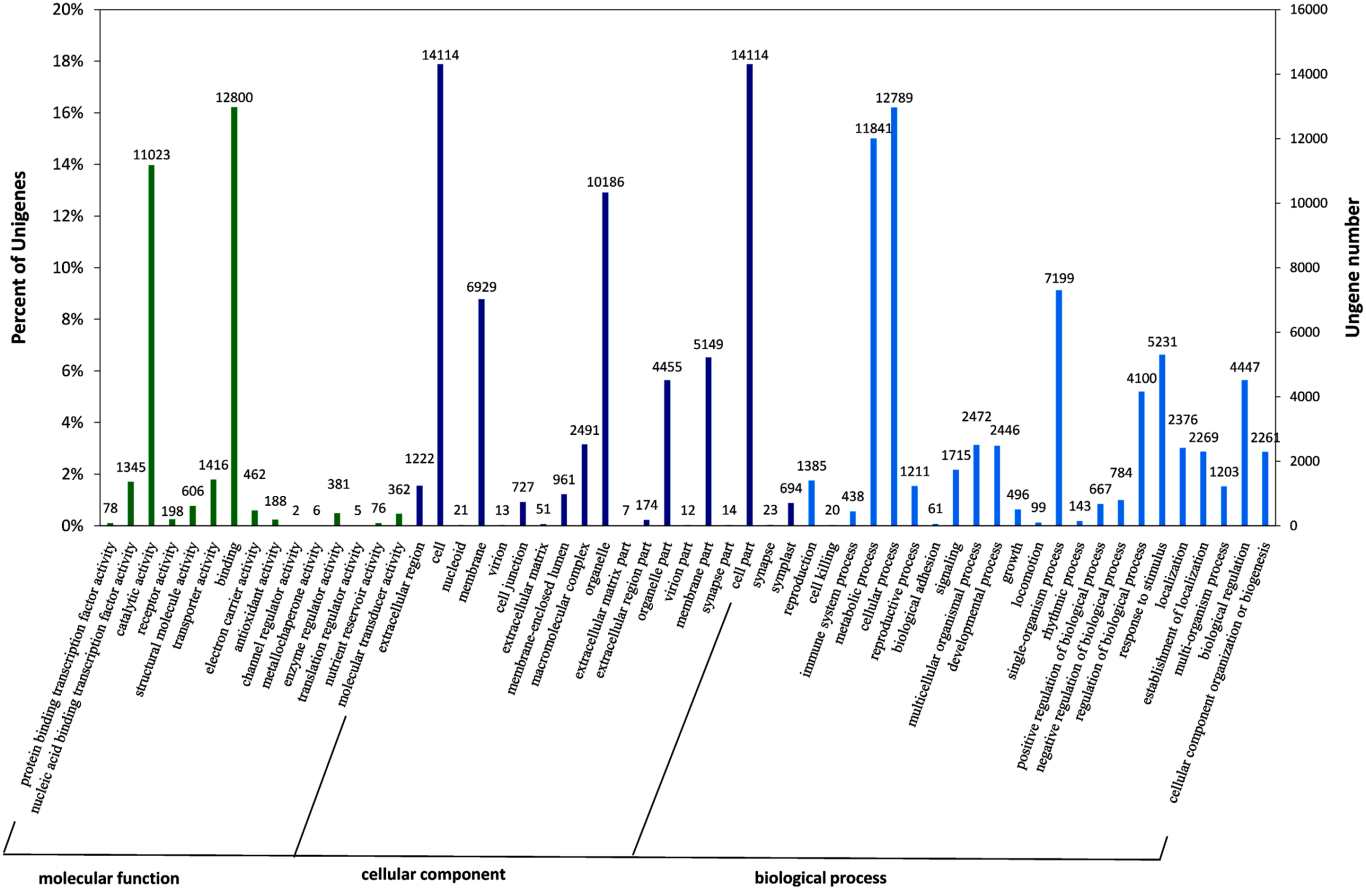
Gene Ontology classification of mung bean transcriptome. Unigenes with BLASTx matches against the plant Nr database were classified into three main GO categories (biological process, cellular component, molecular function) and 57 sub-categories. The left-hand scale on the y-axis shows the percentage of the unigenes in each of the categories. The right-hand scale on the y-axis indicates the number of the unigenes in the same category.

**Fig 3:**
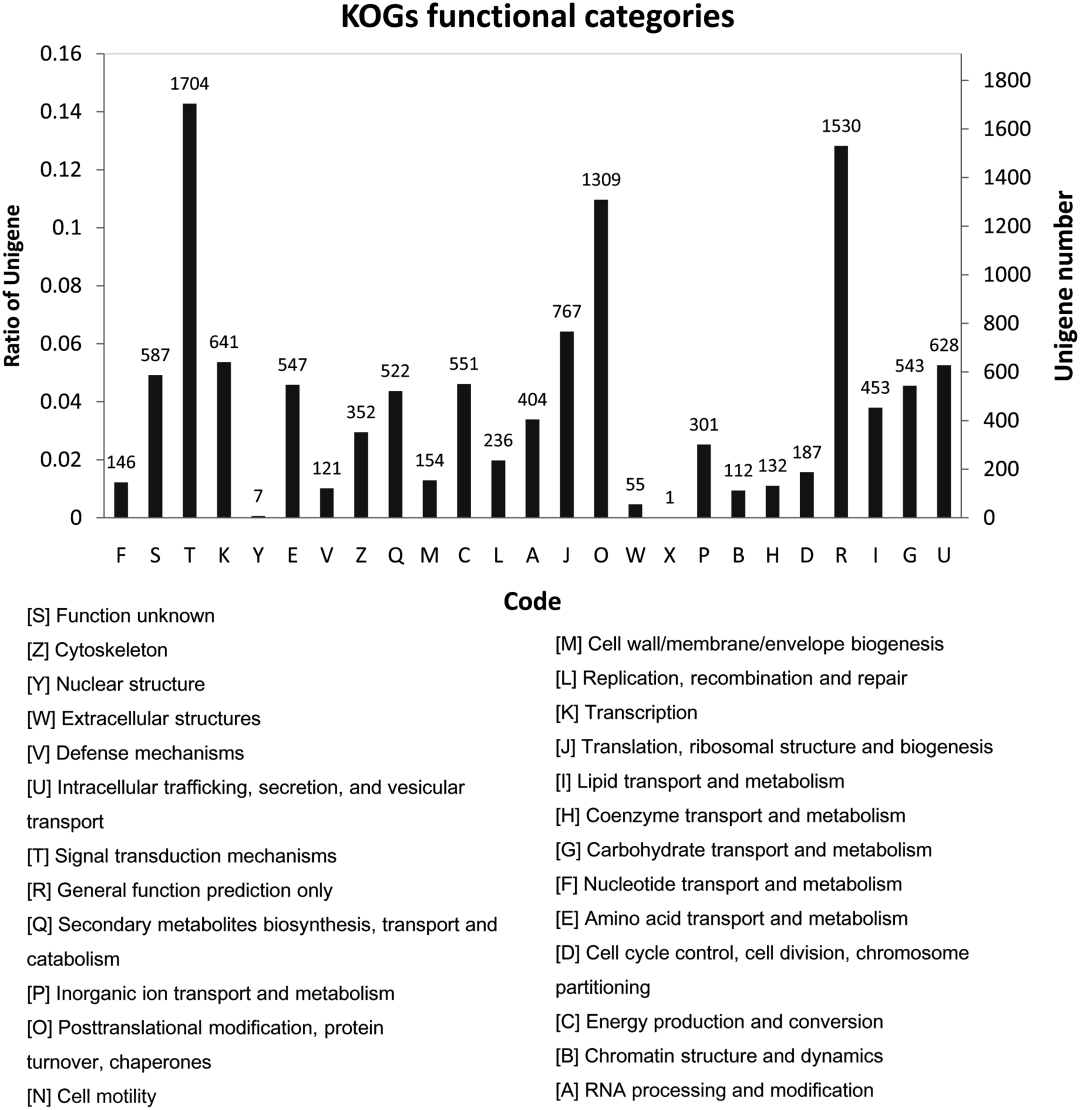
KOG functional classification of the unigenes. Unigenes were assigned to one or more of the 25 COG classification categories.

**Fig 4:**
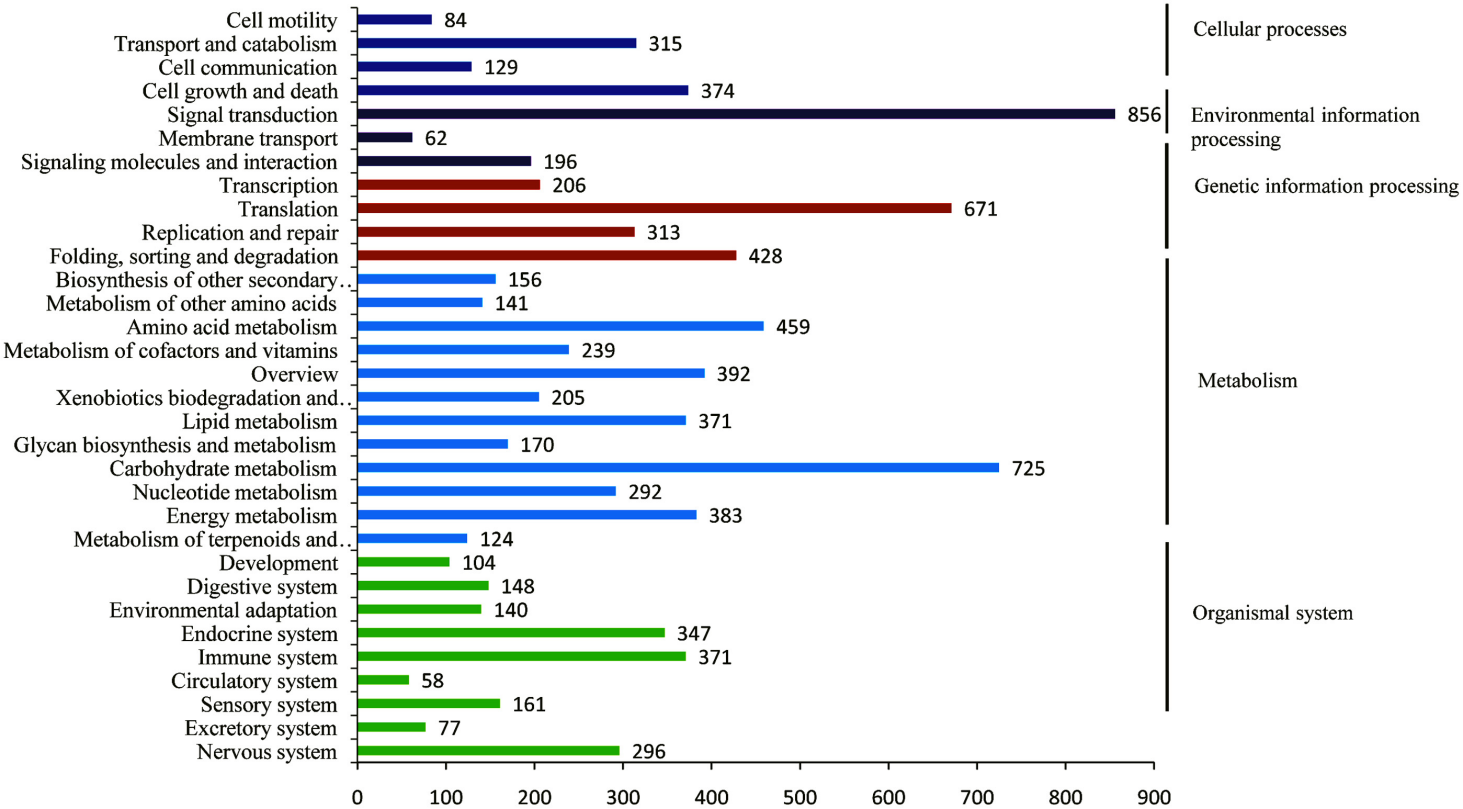
Pathway annotation of the unigenes.

The blast statistics showed that 88.11% of the unigenes exhibited strong homology (E-value < 10–20), and 68.71% exhibited very strong homology (E-value < 10–50) to available plant sequences in the TrEMBL database, most of which belonged to *Glycine max*. The percentage of unigenes with both a bitscore >1000 and an E-value = 0 account for 32.25% (Table 4, S2 Table). The 10 top-hit species based on Nr annotation indicated that 81% of the unigenes can be annotated with sequences from *Glycine max*, while nearly 96% of the unigenes can be annotated with sequences from 5 top-hit species, including *Glycine max*, *Cicer arietinum*, *Medicago truncatula*, *Vitis vinifera*, and *Phaseolus vulgaris* (S4 Fig). Gene ontology (GO) category analysis assigned 61,357, 65,653, and 28,948 unigenes to the GO terms cellular component, biological process, and molecular function, respectively (Fig 2). The top-3 GO subcategories under cellular component are cell (14,114 unigenes, 17.9%), organelle (10,186 unigenes, 12.9%), and cell part (14,114 unigenes, 17.8%). The top-2 GO subcategories under biological process are metabolic process (11,841 unigenes, 15.0%) and cellular process (12,789 unigenes, 16.2%). The top-2 GO subcategories under molecular function are binding (12,800 unigenes, 16.2%) and catalytic activity (11,023, 14.0%). A total of 11,990 unigenes were classed into 25 KOG functional categories, with the top 3 subcategories identified as signal transduction mechanisms (1,704 unigenes, 14.2%), general function prediction only (1,530 unigenes, 12.8%), and posttranslational modification, protein turnover, chaperones (1,309 unigenes, 10.9%) (Fig 3). A total of 7,357 unigenes were annotated to 4,524 KOs (KEGG Orthology), and 4,651 unigenes were mapped into 342 KEGG pathways (Fig 4). The top 10 pathways are presented in Table 5.

**Table 4:** E-value distribution of the BLASTx hits against the Nr and TrEMBL databases for each unigene.

Database	Nr		TrEMBL	
	Unigenes	%	Unigenes	%
E-value = 0	8543	30.38	8622	30.55
E-value <1e-100	5082	18.07	5164	18.30
E-value 1e-100–1e-50	5632	20.03	5606	19.86
E-value 1e-50–1e-20	5456	19.40	5475	19.40
E-value 1e-20–1e-5	3403	12.10	3444	12.20
Total unigenes	28119	100	28225	100
Bitscore>1000, E-value = 0	2749	9.78	2718	9.63
Bitscore>5000 E-value 0-<1e-165	6627	23.57	6384	22.62

**Table 5:** The top 10 pathways with highest percentages of unigenes mapped to.

KO ID	Pathway	Unigene nomber
ko03010	Ribosome	337
ko01230	Biosynthesis of amino acids	180
ko00230	Purine metabolism	173
ko01200	Carbon metabolism	158
ko04141	Protein processing in endoplasmic reticulum	145
ko00190	Oxidative phosphorylation	142
ko04740	Olfactory transduction	135
ko03013	RNA transport	131
ko04075	Plant hormone signal transduction	129
ko03040	Spliceosome	127

### GO enrichment analysis

GO enrichment analysis is a proven method to identify primary biological functions. The functional enrichment of DEGs indicated that, at FDR≤0.05, 258 GOs were enriched in the Wat6 versus Con (Wat6:Con), 183 GOs were enriched in the Wat24 versus Con (Wat24:Con), and 222 GOs were enriched in Wat24 versus Wat6 (Wat24:Wat6). The functional enrichment of the DEGs revealed different GOs in the three samples. For example, the functions of oxidoreductase activity, response to oxidative stress, DNA binding transcription factor activity, and photosynthesis were enriched in Wat6, while the functions of ribosome and translation were enriched in Wat24 (S3 Table). These results suggest that profound cellular and metabolic reorganization occurs during the root induction stage.

**Fig 5:**
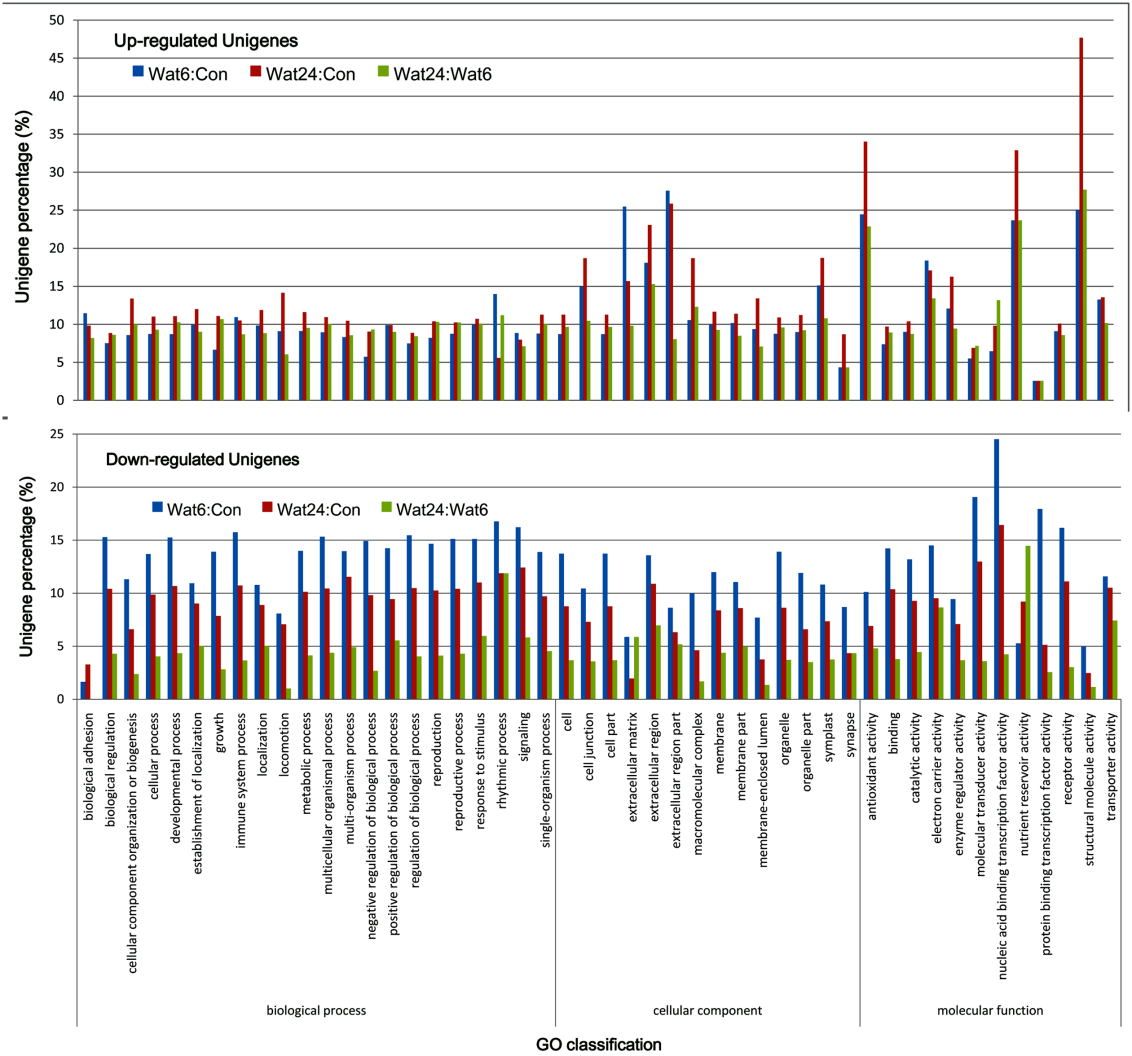
GO enrichment for up- and down-regulated unigenes.

GO enrichment further demonstrated that total of 897 terms were significantly regulated with 595 up-regulated and 302 down-regulated in Wat6:Con, whereas total of 487 terms were significantly regulated with 232 up-regulated and 255 down-regulated in Wat24:Con, and total of 484 terms were significantly regulated with 128 up-regulated and 356 terms down-regulated from Wat6 to Wat24 (Table 6). The up-regulation and down-regulation of GO categories are presented in Fig 5. In the group of down-regulated terms, the proportions of unigenes in each GO category exhibited a trend of Wat6 > Wat24 > Wat24:Wat6, with the exception of the subcategory of nutrient reservoir activity in molecular function, suggesting that the more significant down-regulation of DEGs occurs during the root induction stage. In the group of up-regulated terms, the major GO categories exhibited a trend of Wat24 > Wat6 > Wat24:Wat6, with the exceptions of rhythmic process in biological process and extracellular matrix in cellular component, suggesting that the more significant up-regulation of DEGs occurs during the root induction stage. Comparing between the down-regulated and up-regulated groups, we found that the significant down-regulated categories appeared under molecular function, with the top subcategories of protein binding transcription factor activity, nucleic acid binding transcription factor activity, and molecular transducer activity. The significant up-regulated categories appeared under both cellular component and molecular function, with the top subcategories of antioxidant activity, structural molecule activity, and nutrient reservoir activity in molecular function and extracellular matrix, extracellular region part, cell junction, and macromolecular complex in the cellular component category. The top 10 significant up- and down-regulated GO categories are listed in Table 7. Among the top-10 up-regulated GO groups, GO:0003735, GO:0005840, GO:0005198, GO:0022626, GO:0044445, GO:0006412, GO:0044391, and GO:0030529 were all up-regulated in Wat6, Wat24, and Wat24:Wat6. Moreover, the top-10 up-regulated GO categories were identical in Wat24 and Wat24:Wat6. However, in the down-regulated groups, only GO:0001071 and GO:0003700 were both down-regulated in Wat6 and Wat24; the others were all associated with different GO categories across the three samples (Table 7, S4 Table). These results indicate that nearly the same groups of GO categories were significantly up-regulated at the root induction and initiation stages, including ribosome, structural constituent of ribosome, structural molecule activity, translation, ribonucleoprotein complex, ribosomal subunit, cytosolic ribosome, non-membrane-bounded organelle, intracellular non-membrane-bounded organelle, and cytosolic part. Clearly, these GO categories are associated with protein synthesis. However, several distinct GO categories were significantly down-regulated at the root induction and initiation stages, including nucleic acid binding transcription factor activity, sequence-specific DNA binding transcription factor activity, RNA biosynthetic process, DNA integration, nucleic acid binding transcription factor activity, sequence-specific DNA binding transcription factor activity, and nucleic acid metabolic process. These GO categories are associated with RNA transcription. Interestingly, GO:0016491, oxidoreductase activity, was significantly up-regulated in Wat6 but significantly down-regulated in Wat24:Wat6, suggesting an increase in cellular oxidoreductase activity during the root induction stage that became a decrease during the root initiation stage. Compared with Wat6, the significant down-regulated GO categories include response to chemical stimulus, oxidoreductase activity, response to endogenous stimulus, response to auxin stimulus, response to stimulus, response to hormone stimulus, and response to organic substance. Clearly, these GO categories involve responses to stimulus and hormone signaling.

**Table 6:** Differentially enriched GO categories and KEGG pathways at the time points during adventitious rooting.

Samples	GO (FDR<0.05)			KEGG pathway (FDR<0.05)		
	Total	Down-regulated	up-regulated	Total	down-regulated	up-regulated
**Wat6:Con**	897	302	595	19	5	14
**Wat24:Con**	487	255	232	11	5	6
**Wat24:Wat6**	484	356	128	9	3	6

**Table 7:** Top 10 significantly down- and up-regulated GO categories in the samples.

GO term	Total genes	DEGs	FDR	Level	Description
**Wat6 down-regulation**					
GO:0001071	1345	330	1.08E-26	2	nucleic acid binding transcription factor activity
GO:0003700	1345	330	1.08E-26	3	sequence-specific DNA binding transcription factor activity
GO:0032774	2282	480	1.24E-23	6	RNA biosynthetic process
GO:0006351	2276	479	1.24E-23	6	transcription, DNA-dependent
GO:0044271	2774	550	2.82E-21	5	cellular nitrogen compound biosynthetic process
GO:0034654	2580	511	2.07E-19	5	nucleobase-containing compound biosynthetic process
GO:0018130	2786	542	4.34E-19	5	heterocycle biosynthetic process
GO:0003677	2302	460	5.56E-18	5	DNA binding
GO:0019438	2870	549	8.31E-18	5	aromatic compound biosynthetic process
GO:0015979	192	77	1.32E-17	4	photosynthesis
GO:0003735	471	140	8.19E-35	3	structural constituent of ribosome
GO:0005840	538	149	1.38E-33	4	ribosome
GO:0005198	606	152	4.09E-29	2	structural molecule activity
GO:0016491	1856	320	7.09E-29	3	oxidoreductase activity
GO:0022626	198	77	3.09E-27	5	cytosolic ribosome
GO:0044445	243	82	3.37E-24	5	cytosolic part
GO:0006412	671	151	8.03E-24	6	translation
GO:0044391	216	76	9.29E-24	4	ribosomal subunit
GO:0005576	1222	221	6.56E-22	2	extracellular region
GO:0030529	801	161	3.92E-20	3	ribonucleoprotein complex
**Wat24 down-regulation**					
GO:0015074	1125	201	1.01E-15	6	DNA integration
GO:0001071	1345	221	9.96E-14	2	nucleic acid binding transcription factor activity
GO:0003700	1345	221	9.96E-14	3	sequence-specific DNA binding transcription factor activity
GO:0090304	4585	585	9.96E-14	5	nucleic acid metabolic process
GO:1901360	5919	722	1.50E-13	4	organic cyclic compound metabolic process
GO:0046483	5667	693	4.67E-13	4	heterocycle metabolic process
GO:0006139	5376	660	1.40E-12	4	nucleobase-containing compound metabolic process
GO:0006725	5817	704	2.03E-12	4	cellular aromatic compound metabolic process
GO:0034641	5701	689	6.26E-12	4	cellular nitrogen compound metabolic process
GO:0006351	2276	322	7.93E-12	6	transcription, DNA-dependent
**Wat24 up-regulation**					
GO:0005840	538	297	2.94E-139	4	ribosome
GO:0003735	471	277	2.94E-139	3	structural constituent of ribosome
GO:0005198	606	289	1.48E-113	2	structural molecule activity
GO:0006412	671	288	7.86E-99	6	translation
GO:0030529	801	312	3.08E-94	3	ribonucleoprotein complex
GO:0044391	216	146	4.33E-84	4	ribosomal subunit
GO:0022626	198	132	1.15E-74	5	cytosolic ribosome
GO:0043228	1628	418	2.78E-63	3	non-membrane-bounded organelle
GO:0043232	1628	418	2.78E-63	4	intracellular non-membrane-bounded organelle
GO:0044445	243	136	4.18E-63	5	cytosolic part
**Wat24:Wat6 down-regulation**					
GO:0042221	2287	182	3.10E-14	3	response to chemical stimulus
GO:0016491	1856	156	4.56E-14	3	oxidoreductase activity
GO:0009719	1364	117	1.32E-10	3	response to endogenous stimulus
GO:0009733	367	50	1.97E-10	5	response to auxin stimulus
GO:0050896	5231	312	1.72E-09	2	response to stimulus
GO:0009725	1235	105	2.38E-09	4	response to hormone stimulus
GO:0010033	1584	125	2.60E-09	4	response to organic substance
GO:0080167	105	24	3.30E-09	4	response to karrikin
GO:0010378	9	8	2.57E-08	4	temperature compensation of the circadian clock
GO:0071365	247	36	2.57E-08	6	cellular response to auxin stimulus
**Wat24:Wat6 up-regulation**					
GO:0005840	538	170	2.50E-45	4	ribosome
GO:0003735	471	153	1.95E-42	3	structural constituent of ribosome
GO:0005198	606	168	7.43E-37	2	structural molecule activity
GO:0006412	671	166	7.35E-30	6	translation
GO:0030529	801	177	1.26E-25	3	ribonucleoprotein complex
GO:0044391	216	77	1.31E-23	4	ribosomal subunit
GO:0022626	198	69	1.67E-20	5	cytosolic ribosome
GO:0043228	1628	273	1.67E-20	3	non-membrane-bounded organelle
GO:0043232	1628	273	1.67E-20	4	intracellular non-membrane-bounded organelle
GO:0044445	243	70	1.20E-15	5	cytosolic part

### KEGG enrichment analysis

Pathway enrichment analysis revealed that 9, 11, and 9 pathways were the significant difference pathways enriched in Wat6, Wat24, and Wat24:Wat6, respectively. Further analysis indicated that 5, 5, and 3 pathways were significantly (RDF ≤0.05) down-regulated and 14, 6, and 6 pathways were significantly up-regulated in Wat6, Wat24, and Wat24:Wat6, respectively (Table 6). These results indicate that more KOs were up-regulated than down-regulated, especially in Wat6, suggesting that the key up-regulation of KOs occurred during the root induction stage. KEGG enrichment analysis further indicated that ko03010 (ribosome), ko0094 (phenylpropanoid biosynthesis), ko00360 (phenylalanine metabolism), and ko00909 (sesquiterpenoid and triterpenoid biosynthesis) were all up-regulated in Wat6, Wat24, and Wat24:Wat6. The significant down-regulated KOs during Wat6 were photosynthesis, carbon fixation in photosynthetic organisms, carotenoid biosynthesis, nitrogen metabolism, sphingolipid metabolism, glycerolipid metabolism, and porphyrin and chlorophyll metabolism. The significant down-regulated KOs during Wat24 were cutin, diterpenoid biosynthesis, cytokine-cytokine receptor interaction, and circadian rhythm—plant, and those in Wat24:Wat6 were oxidative phosphorylation, nitrogen metabolism, plant hormone signal transduction, diterpenoid biosynthesis, photosynthesis, and cysteine and methionine metabolism. Among them, ko00195 (photosynthesis) and ko00910 (nitrogen metabolism) were down-regulated in both Wat6 and Wat24:Wat6, suggesting that photosynthesis and nitrogen metabolism were continuously down-regulated from the root induction stage to the root initiation stage (Tables 8 and 9, S5 Table). The principal aspects of the KEGG enrichment results were consistent with the GO enrichment results.

**Table 8:** The pathway enrichment of the DEGs (FDR<0.05).

KO ID	Total gene	DEGs	FDR	Description
**Wat6**				
ko00195	53	29	0.000197	Photosynthesis
ko00940	69	33	0.0009	Phenylpropanoid biosynthesis
ko03010	337	112	0.000918	Ribosome
ko00910	31	18	0.002374	Nitrogen metabolism
ko00360	57	25	0.026989	Phenylalanine metabolism
ko00909	7	6	0.045994	Sesquiterpenoid and triterpenoid biosynthesis
**Wat24**				
ko03010	337	217	2.52E-66	Ribosome
ko00940	69	40	1.05E-08	Phenylpropanoid biosynthesis
ko00360	57	32	1.74E-06	Phenylalanine metabolism
ko00904	8	7	0.0122	Diterpenoid biosynthesis
ko00909	7	6	0.032752	Sesquiterpenoid and triterpenoid biosynthesis
ko00073	12	8	0.046727	Cutin
ko00363	20	11	0.046727	Bisphenol degradation
ko00945	26	13	0.046727	Stilbenoid
ko00627	29	14	0.046727	Aminobenzoate degradation
**Wat24:Wat6**				
ko03010	337	118	9.35E-24	Ribosome
ko00940	69	28	2.24E-06	Phenylpropanoid biosynthesis
ko00360	57	21	0.000618	Phenylalanine metabolism

**Table 9:** Top significantly up- and down-regulated KOs (p <0.01) in the three samples.

KO ID	Total gene	DEGs	P-value	FDR	Description
**Wat6 down-regulation**					
ko00195	53	20	1.51E-06	0.0004	Photosynthesis
ko00710	46	15	0.0002	0.0149	Carbon fixation in photosynthetic organisms
ko00906	20	9	0.0003	0.0149	Carotenoid biosynthesis
ko00910	31	10	0.0027	0.1250	Nitrogen metabolism
ko00600	34	10	0.0057	0.1740	Sphingolipid metabolism
ko00561	52	13	0.0078	0.1867	Glycerolipid metabolism
ko00860	58	14	0.0082	0.1867	Porphyrin and chlorophyll metabolism
**Wat6 up-regulation**					
ko03010	337	110	4.42E-29	1.02E-26	Ribosome
ko00940	69	25	2.66E-08	3.07E-06	Phenylpropanoid biosynthesis
ko00360	57	21	2.55E-07	1.96E-05	Phenylalanine metabolism
ko00980	40	13	0.0002	0.0078	Metabolism of xenobiotics by cytochrome P450
ko00909	7	5	0.0003	0.0078	Sesquiterpenoid and triterpenoid biosynthesis
ko00480	63	17	0.0003	0.0078	Glutathione metabolism
ko04610	18	8	0.0003	0.0078	complement and coagulation cascades
ko00270	68	17	0.0009	0.0181	Cysteine and methionine metabolism
ko00260	52	14	0.0011	0.0216	Glycine
ko00750	10	5	0.0025	0.0451	Vitamin B6 metabolism
ko00920	29	9	0.0030	0.0496	Sulfur metabolism
ko01230	180	32	0.0040	0.0610	Biosynthesis of amino acids
**Wat24 down-regulation**					
ko00073	12	8	1.40E-06	0.0002	Cutin
ko00904	8	6	1.20E-05	0.0011	Diterpenoid biosynthesis
ko04060	58	15	0.0001	0.0081	Cytokine-cytokine receptor interaction
ko04712	23	7	0.0031	0.1194	Circadian rhythm—plant
**Wat24 up-regulation**					
ko03010	337	212	5.34E-115	1.46E-112	Ribosome
ko00940	69	34	1.92E-13	2.63E-11	Phenylpropanoid biosynthesis
ko00360	57	27	2.00E-10	1.82E-08	Phenylalanine metabolism
ko00909	7	5	0.0006	0.0276	Sesquiterpenoid and triterpenoid biosynthesis
ko04110	94	21	0.0077	0.2684	Cell cycle
ko00040	49	13	0.0078	0.2684	Pentose and glucuronate interconversions
**Wat24:Wat6 down-regulation**					
ko00190	143	16	0.0003	0.0293	Oxidative phosphorylation
ko00910	31	6	0.0017	0.0753	Nitrogen metabolism
ko04075	130	13	0.0033	0.1095	Plant hormone signal transduction
ko00904	8	3	0.0036	0.1095	Diterpenoid biosynthesis
ko00195	53	7	0.0067	0.1720	Photosynthesis
ko00270	68	8	0.0077	0.1743	Cysteine and methionine metabolism
**Wat24:Wat6 up-regulation**					
ko03010	337	117	5.20E-43	1.32E-40	Ribosome
ko00940	69	21	3.96E-07	5.03E-05	Phenylpropanoid biosynthesis
ko00360	57	15	0.0001	0.0077	Phenylalanine metabolism
ko04110	94	20	0.0002	0.0122	Cell cycle
ko00052	38	11	0.0004	0.0170	Galactose metabolism
ko00500	119	19	0.0108	0.3036	Starch and sucrose metabolism

### Gene expression profiling during adventitious rooting

Gene expression levels can be estimated from Illumina sequencing based on the number of clean reads for a gene. The RPKM method [15] was used to calculate the expression abundances of unigenes during adventitious rooting. The results indicated that the unigenes numbered with RPKM = 100–500, RPKM = 500–1000, and RPKM≥1000 exhibited a clearly increasing trend from Con to Wat24, suggesting that the expression abundances of certain genes greatly increased during root development (Table 3). A total of 11,717 unigenes showed differential expression (log2 ≥1) in Wat6, with 8,772 unigenes down-regulated and 2,945 unigenes up-regulated. A total of 12,737 unigenes showed differential expression during Wat24, with 9,303 unigenes down-regulated and 3,434 unigenes up-regulated. Compared with Wat6, a total of 5,334 unigenes showed differential expression in the Wat24 sample, with 2,167 unigenes down-regulated and 3,167 unigenes up-regulated. These results indicate that 74.9% and 73.04% of the DEGs were down-regulated at the root induction and initiation stages, respectively, while 59.4% of the DEGs were up-regulated from the root induction stage to the initiation stage (Table 10). Further analysis revealed that 283 unigenes were specifically up-regulated DEGs and 546 unigenes were specifically down-regulated DEGs in Wat6; 619 and 753 unigenes were specifically up- and down-regulated DEGs in Wat24; and 424 and 163 unigenes were specifically up- and down-regulated DEGs from Wat6 to Wat24. Most of the specifically expressed DEGs were low-abundance genes (read number ≤100). For example, among the specifically expressed DEGs with a read number ≥100, 34 were up-regulated and 11 were down-regulated in Wat6, 69 were up-regulated and 11 were down-regulated in Wat24, and 29 were up-regulated and 0 were down-regulated from Wat6 to Wat24. Moreover, among the specifically expressed DEGs with both a read number ≥100 and log2 ≥4, 209 unigenes were up-regulated and 96 were down-regulated in Wat6, 238 were up-regulated and 59 were down-regulated in Wat24, and 100 were up-regulated and 34 were down-regulated from Wat6 to Wat24 (Table 10). These results indicate that many more specific DEGs were significantly up-regulated than down-regulated during adventitious root induction and initiation.

**Table 10:** Statistics of the DEGs (FDR<0.001) at the time points during adventitious rooting in mung bean.

Samples	DEGs	Log2	NA	≥5	≥4	≥3	≥2	≥1	Total	Total DEGs
**Wat6:Con**	down	total	546	48	226	694	1846	5412	8772	11717
		reads≥100	11	28	68	177	788	3954	5026	
	up	total	283	149	161	277	514	1561	2945	
		reads≥100	34	116	93	131	324	1265	1963	
**Wat24:Con**	down	total	753	48	270	834	2128	5270	9303	12737
		reads≥100	11	16	43	124	689	3415	4298	
	up	total	619	195	168	336	557	1559	3434	
		reads≥100	69	14	224	169	326	1284	2086	
**Wat24:Wat6**	down	total	163	29	59	205	431	1280	2167	5334
		reads≥100	0	15	19	60	167	844	1105	
	up	total	424	90	122	250	634	1647	3167	
		reads≥100	29	54	46	130	397	1328	1984	

### Specifically up- and down-regulated unigenes during adventitious root induction

To evaluate the changes in DEGs during adventitious root induction and initiation, we selected the top 50 DEGs with both a read number >1000 and log2 >5 (fold change >32) (S6 Table). After filtering out the unigenes termed hypothetical protein, uncharacterized protein, and unknown in the database, the remaining DEGs are listed in Tables 11, 12 and 13. Among the top-25 genes with more than 32-fold up-regulation in the Wat6 sample, the most abundantly expressed genes (read number >1000) include five cationic peroxidase genes (Vr39448, Vr31128, Vr22610, Vr39339, and Vr39180), two pathogenesis-related protein genes (Vr39039 and Vr36526), two anthocyanin metabolism-associated genes (Vr36323 and Vr36176), and two isoflavone metabolism-associated genes (Vr38993 and Vr35207). The other important genes include basic chitinase class 3 (Vr40472) and trypsin protease inhibitor precursor (Vr35851). It is worth noting that an auxin-related gene, auxin efflux carrier (Vr21159), was significantly up-regulated. However, only six genes with more than 32-fold down-regulation appeared in the top DEGs list, including three MYB transcription factor genes (Vr40489, Vr39799, and Vr13836), polyprotein precursor gene (Vr38043), S-type anion channel SLAH3-like gene (Vr24590), and auxin-induced protein 5NG4-like gene (Vr55469) (Table 12). The other genes with more than 16-fold down-regulation include heat shock 70 kDa protein-like (Vr40796 and Vr42894), ABC transporter G family member 22-like (Vr50534), serine glyoxylate aminotransferase 2 (Vr41217), probable E3 ubiquitin-protein ligase HERC1-like (Vr15096), putative organic cation transport protein (Vr56588), and histidine kinase 1-like isoform X2 (Vr33063) (S6 and S7 Tables).

**Table 11:** Top up-and down-regulated DEGs in the Wat6 sample.

Gene ID	Reads	RPKM	Log2	P-value	FDR	Nr references	Functional description
Vr39448	973	53.6	NA	0	0	XP_004500339.1	cationic peroxidase 1-like
Vr39180	6952	155	12.05	1.6E-15	1.3E-14	XP_003538325.1	cationic peroxidase 1-like
Vr41762	1370	16.4	10.71	2.2E-16	2E-15	XP_003556683.1	polygalacturonase-like
Vr22610	7681	140	9.50	0	0	XP_003544327.1	cationic peroxidase 1-like
Vr49226	1028	11.5	8.71	0	0	XP_003525322.1	cytochrome P450 716B2-like
Vr39039	10890	210	8.00	6.2E-14	4.9E-13	ADX66343.1	pathogenesis-related protein
Vr50416	1313	13.3	7.06	0	0	XP_003548705.1	D-inositol-3-phosphate glycosyltransferase-like
Vr49784	1553	35.1	6.72	1.3E-14	1.1E-13	XP_003543377.1	cysteine-rich repeat secretory protein 38
Vr40472	7732	106	6.44	0	0	CAA61279.1	basic chitinase class 3
Vr36526	10690	486	6.23	5.8E-13	4.4E-12	ABS70717.1	pathogen-related protein
Vr36176	5444	63.3	6.13	0	0	XP_003529158.1	leucoanthocyanidin dioxygenase-like
Vr13687	4326	32.1	6.10	0	0	XP_003531311.1	low affinity cationic amino acid transporter 2-like
Vr35851	43774	752	6.02	0	0	NP_001237786.1	Kunitz trypsin protease inhibitor precursor
Vr36323	1573	17	5.78	4.5E-14	3.6E-13	XP_003552581.1	anthocyanin 5-aromatic acyltransferase-like
Vr39225	5616	104	5.69	6.4E-13	4.9E-12	XP_003534655.1	cationic peroxidase 1-like
Vr21159	1088	15.1	5.57	5.9E-14	4.7E-13	ABN08535.1	auxin efflux carrier
Vr41271	2487	38.4	5.53	0	0	ACM89628.1	TIR-NBS-LRR type disease resistance protein
Vr41972	3151	43.1	5.37	0	0	XP_003536550.1	thiazole biosynthetic enzyme, chloroplastic-like
Vr28474	1074	8.72	5.36	4.9E-14	3.9E-13	XP_003543367.1	equilibrative nucleoside transporter 3-like
Vr31128	12075	169	5.33	4.7E-12	3.3E-11	XP_003547616.1	cationic peroxidase 2-like
Vr31555	1542	14.9	5.30	9.5E-14	7.5E-13	XP_003521608.1	sex determination protein tasselseed-2-like
Vr38993	15083	141	5.18	9.5E-12	6.4E-11	NP_001240000.1	isoflavone 2'-hydroxylase-like
Vr39339	6519	80.2	5.13	7.2E-13	5.4E-12	NP_001236520.1	vestitone reductase
Vr42500	4293	44.5	5.07	1.3E-12	9.8E-12	XP_003547675.1	lysine histidine transporter 1-like
Vr35207	18340	258	5.07	1.6E-11	1.1E-10	XP_003537598.1	isoflavone reductase-like
Vr38043	12585	19.59	-10.33	0	0	ACE95704.1	polyprotein precursor
Vr24590	2030	13.88	-5.89	0	0	XP_003535586.1	S-type anion channel SLAH3-like
Vr40489	13321	223.86	-5.77	0	0	NP_001236400.1	MYB transcription factor MYB114
Vr39799	21964	249.38	-5.57	0	0	ABH02878.1	MYB transcription factor MYB134
Vr13836	4622	75.72	-5.49	0	0	ABH02918.1	MYB transcription factor MYB114
Vr55469	3578	27.63	-5.31	0	0	XP_003537193.1	auxin-induced protein 5NG4-like isoform 1

**Table 12:** Top up- and down-regulated DEGs in the Wat24 sample.

Gene ID	Reads	RPKM	Log2	P-value	FDR	Nr references	Functional description
Vr43029	5100	160.26	NA	4.4E-16	4.4E-15	XP_003529031.1	patatin group A-3-like
Vr39448	4245	234.11	NA	4.4E-16	4.4E-15	XP_004500339.1	cationic peroxidase 1-like
Vr44006	2547	51.04	NA	4.4E-16	4.4E-15	BAJ22384.1	terminal flower 1a
Vr58791	1221	15.62	NA	4.4E-16	4.4E-15	XP_003529031.1	patatin group A-3-like
Vr39180	7480	166.84	12.2	4.4E-16	4.4E-15	XP_003538325.1	cationic peroxidase 1-like
Vr41762	1669	20.04	11	8.9E-16	8.6E-15	XP_003556683.1	polygalacturonase-like
Vr22610	20516	372.85	10.91	0	0	XP_003544327.1	cationic peroxidase 1-like
Vr13838	7752	70.042	9.75	0	0	XP_003518665.1	polygalacturonase At1g48100-like
Vr34411	2782	28.56	9.15	0	0	XP_003521576.1	endoglucanase 17-like
Vr42199	1858	23.72	7.34	0	0	XP_003528650.1	ethylene-responsive transcription factor ERF086-like
Vr50416	1158	11.77	6.88	0	0	XP_003548705.1	D-inositol-3-phosphate glycosyltransferase-like
Vr46251	2892	28.53	6.88	5.4E-14	4.8E-13	XP_003517028.1	pectinesterase 2-like
Vr39225	12040	223.7	6.79	6.3E-13	5.2E-12	XP_003534655.1	cationic peroxidase 1-like
Vr34889	13312	225.24	6.72	1.4E-12	1.1E-11	P29024.1	acidic endochitinase
Vr39778	1532	91.33	6.62	1.7E-14	1.6E-13	XP_003552297.1	cationic peroxidase 1-like
Vr39039	3989	76.95	6.55	6.3E-14	5.5E-13	ADX66343.1	pathogenesis-related protein
Vr38890	10070	114.16	6.48	7.6E-13	6.2E-12	XP_003536300.1	7-ethoxycoumarin O-deethylase-like
Vr45152	1579	33.99	6.46	3.6E-14	3.3E-13	XP_003527180.1	blue copper protein-like
Vr39755	5637	64.135	6.23	0	0	NP_001238091.1	polygalacturonase PG1 precursor
Vr18948	1081	7.771	6.2	1.1E-14	1E-13	XP_003553810.1	potassium transporter 5-like
Vr35851	46041	791.63	6.09	0	0	NP_001237786.1	Kunitz trypsin protease inhibitor precursor
Vr41032	2736	51.69	6.04	8E-14	7E-13	XP_003535070.1	peroxidase C3-like isoform 2
Vr31555	2186	21.09	5.8	9.5E-14	8.2E-13	XP_003521608.1	sex determination protein tasselseed-2-like
Vr40216	2120	66.18	5.79	8.7E-14	7.6E-13	XP_003535071.1	peroxidase C3-like isoform 3
Vr31128	16176	226.783	5.76	4.8E-12	3.6E-11	XP_003547616.1	cationic peroxidase 2-like
Vr42825	2087	36.88	5.76	8.7E-14	7.6E-13	XP_003542895.1	endo-1,3;1,4-beta-D-glucanase-like
Vr36698	1294	29.65	5.54	4.6E-14	4.1E-13	P0DI40.1	casparian strip membrane protein 2
Vr40472	4060	55.66	5.51	0	0	CAA61279.1	basic chitinase class 3
Vr34521	1E+05	2117.22	5.48	2.7E-11	1.8E-10	AAA66288.1	proline-rich protein
Vr36526	5962	270.99	5.39	5.6E-13	4.7E-12	ABS70717.1	pathogen-related protein
Vr59584	1296	21.83	5.20	7.3E-14	6.4E-13	XP_003543376.1	early nodulin-like protein 1-like
Vr41271	1918	29.65	5.15	0	0	ACM89628.1	TIR-NBS-LRR type disease resistance protein
Vr44815	1059	8.48	5.13	5.1E-14	4.5E-13	XP_003529133.1	laccase-9-like
Vr38043	12585	19.59	-7.35	0	0	ACE95704.1	polyprotein precursor
Vr48206	3644	65.60	-5.41	0	0	XP_003523924.1	auxin-induced protein 5NG4-like

**Table 13:** Top up- and down-regulated DEGs between Wat24 and Wat6 sample.

Gene ID	Reads/Wat24	RPKM	Log2	P-value	FDR	Nr references	Description
Vr68124	988	22.01	NA	0	0	P0DI41.1	casparian strip membrane protein 3
Vr51177	244	4.99	NA	0	0	XP_003528524.1	auxin-binding protein ABP19a-like
Vr36698	1294	29.64	8.34	0	0	P0DI40.1	casparian strip membrane protein 2
Vr44673	3255	61.19	7.76	2.66E-15	4.34E-14	XP_003539771.1	vignain-like
Vr59584	1296	21.83	7.53	0	0	XP_003543376.1	early nodulin-like protein 1-like
Vr34411	2782	28.56	7.12	2.35E-14	3.69E-13	XP_003521576.1	endoglucanase 17-like
Vr22476	1023	10.96	6.09	2.66E-15	4.34E-14	XP_003556105.1	low-temperature-induced 65 kDa protein-like
Vr35442	868	24.32	5.96	0	0	AAF81194.1	LEA-18
Vr35419	972	12.57	5.60	2.35E-14	3.69E-13	AAU94657	ef1a
Vr41972	3151	43.11	-8.30	0	0	XP_003536550.1	thiazole biosynthetic enzyme, chloroplastic-like
Vr49846	2439	19.96	-5.04	0	0	NP_001235886.1	circadian clock-associated FKF1

### Specifically up- and down-regulated unigenes during adventitious root initiation

There were 33 highly abundant (read number >1000) genes with more than 32-fold (log2 > 5) up-regulation in the Wat24 sample. Similar to the Wat6 sample, six cationic peroxidase 1-like genes, two pathogen-related protein genes, a polygalacturonase gene, a polygalacturonase PG1 precursor gene, a basic chitinase class 3 gene, and a trypsin protease inhibitor precursor gene were all significantly up-regulated in the Wat24 sample. However, many other genes were exclusively up-regulated in Wat24, such as patatin group A-3-like (Vr43029 and Vr58791), ethylene-responsive transcription factor ERF086-like (Vr42199), 7-ethoxycoumarin O-deethylase-like (Vr38890), potassium transporter 5-like (Vr18948), peroxidase C3-like isoform 2 (Vr40216 and Vr41032), casparian strip membrane protein 2 (Vr36698), and proline-rich protein (Vr34521). Only two genes, polyprotein precursor (Vr38043) and auxin-induced protein 5NG4-like (Vr48206), which were also observed in Wat6, were down-regulated more than 32-fold (Table 12; S5 and S6 Tables).

We further analyzed the DEGs between the Wat6 and Wat24 samples. Seven genes with a read number >1000 were up-regulated by more than 32-fold from Wat6 to Wat24, including casparian strip membrane protein 2 (Vr36698), vignain-like (Vr44673), early nodulin-like protein 1-like (Vr59584), low-temperature-induced 65 kDa protein-like (Vr34411), LEA-18 (Vr35442), and ef1a (Vr35419) (Table 13). In addition, the genes auxin-binding protein ABP19a-like (Vr51177) and casparian strip membrane protein 3 (Vr68124) were specifically expressed in Wat24 compared with Wat6. A number of the important genes that exhibited highly abundant expression and more than 16-fold up-regulation include two heat shock 70 kDa protein-like genes (Vr42894 and Vr40796), two MYB transcription factor MYB114 genes (Vr40489 and Vr39799), two patatin group A-3-like genes (Vr43029 and Vr42547), a metacaspase-9-like gene (Vr39095), and a probable E3 ubiquitin-protein ligase HERC1-like gene (Vr15096) (S6 Table). Compared with the Wat6 sample, only two genes with reads >1000 were down-regulated by more than 32-fold, including a thiazole biosynthetic enzyme gene, chloroplastic-like gene (Vr41972), and circadian clock-associated FKF1 gene (Vr49846) (Table 13). Four genes with reads >1000 were down-regulated more than 16-fold: formate dehydrogenase (Vr13406), GIR1 (Vr38378), beta-glucosidase 47-like (Vr41355), and GDSL esterase/lipase (Vr45510) (S6 and S7 Tables).

### Validation of gene expression

**Fig 6:**
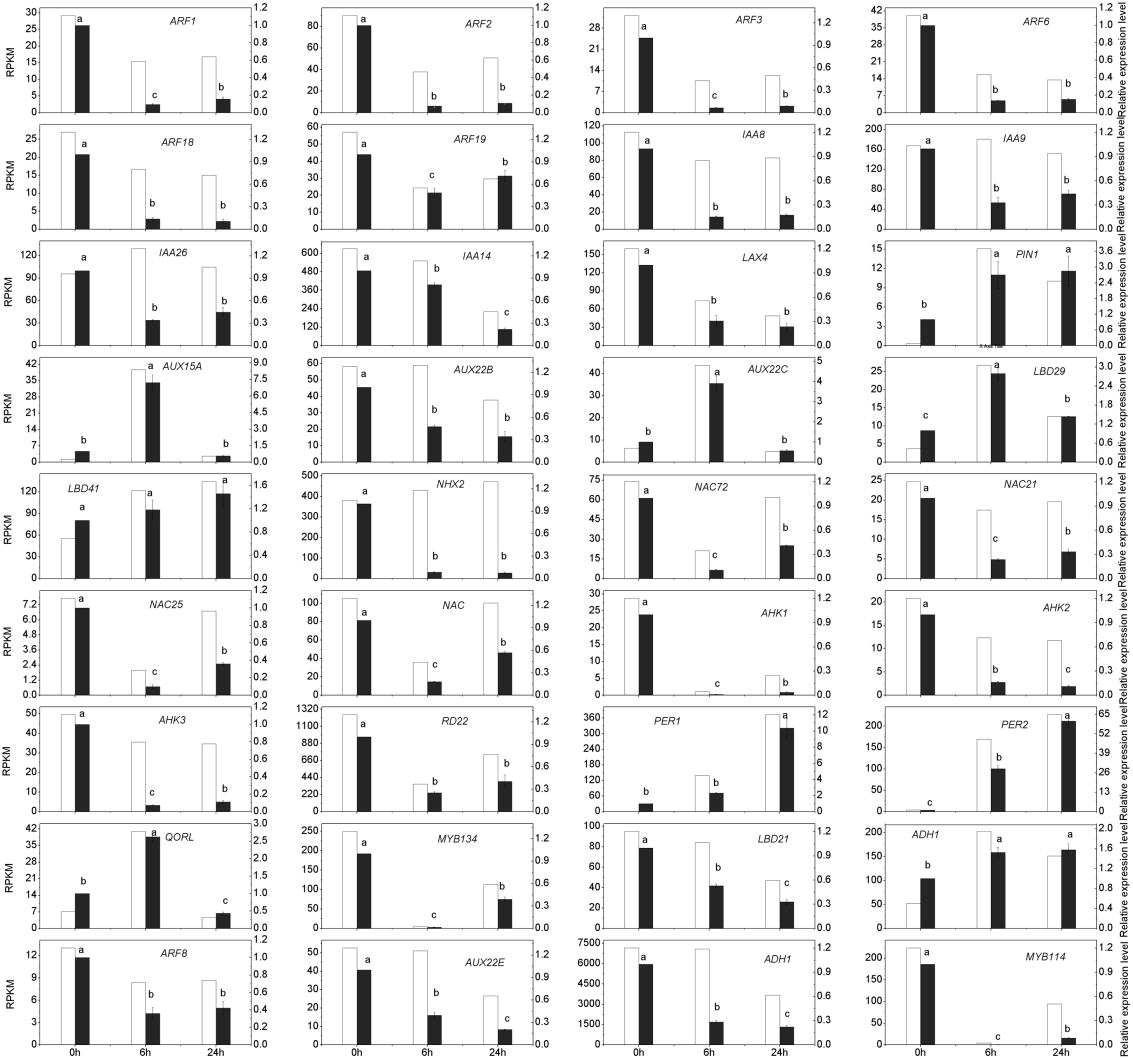
Validation of selected genes involved in adventitious rooting by qRT-PCR. The gene expression levels measured by qRT-PCR were compared with that of RNA-Seq. White histograms represent expression levels determined by RNA-Seq in RPKM units (left axis), while grey columns represent gene expression levels determined by qRT-PCR and normalized to three control genes (right axis). Bars represent the mean (± SE) of three experiments. Different letters (a, b, and c) represent statistically significant differences (P < 0.01) among the data of qRT-PCR, analysed using Student’s t-test.

To validate the differential expression data obtained through statistical comparisons of RPKM values, a total of 39 interesting DEGs of four types: 17 auxin signaling-related genes, 14 stress response-related genes, 3 LATERAL ORGAN BOUNDARY (LBD)-DOMAIN genes, and 3 internal reference genes were selected for validation of the transcriptomic data using real-time quantitative PCR (qRT-PCR). Detailed information on these genes is presented in S8 Table. According to the RNA-Seq results and the study published by Jian et al. [45], we selected three genes: *CPY20*, *eIF*5*A*, and *ACTIN* (*Actin-related protein 4*), as internal reference genes for qRT-PCR. The qRT-PCR results showed that *CPY20* was the most stable housekeeping gene, so it was used to calculate the relative expression levels in this study. Out of the 39 selected genes, 36 showed a strong correlation (92.3%) to the RNA-Seq data (Fig 6). The qRT-PCR results confirmed that *PER1*, *PER2*, *ADH1*, *LBD29*, *LBD41*, and *PIN1* were significantly up-regulated at the two time points; *AUX22C*, *AUX15A*, and *QORL* (Quinone oxidoreductase-like protein) were significantly up-regulated at Wat6 but returned to their original levels by Wat24; and the other genes showed a significant reduction at both time points.

## Discussion

Transcriptomic data can reveal gene expression profiles and give fundamental insights into biological processes. As a high-throughput, accurate and low-cost method, RNA-Seq, a new next-generation sequencing (NGS) method, has been widely applied to analyze transcriptomes qualitatively and quantitatively. NGS has proven to be a powerful tool for DEG screening, especially for species without available genomic information [42, 43]. In this study, the Illumina HiSeq 2000 platform was used to perform a *de novo* transcriptome sequencing analysis of the mung bean to better understand gene expression changes during adventitious rooting. Pooled RNA samples from hypocotyls and hypocotyls sampled at two time points after primary root excision were used to construct cDNA libraries for deep sequencing. This sequencing generated 7.36 Gbp, 5.998 Gbp, and 5.885 Gbp of sequence data, and obtained approximately 68.32 million, 55.58 million, and 55.57 million paired-end clean reads in the mung bean hypocotyls 0 h, 6 h, and 24 h after primary root excision, respectively. The newly developed Trinity method was used for *de novo* reads assembly. The Trinity method can recover more full-length transcripts across a broad range of expression levels and provides a unified, sensitive solution for transcriptome reconstruction in species without a reference genome, similar to methods that rely on genome alignments [26]. Another study demonstrated that Trinity was a better approach than was SOAPdenovo for assembly, as the assembled unigenes did not contain gaps, and the average unigene length was nearly twice the length of those produced by SOAPdenovo [28]. After *de novo* assembly, we obtained 78,697 unigenes with a mean length of 832 bp, which is longer than has been reported previously in studies using the same technology [26, 28, 42, 43]. Among the total number of unigenes, 91.92% (72,342), 84.71% (66,663), and 82.19% (64,680) of the unigenes were expressed in the Con, Wat6, and Wat24 samples, respectively. Consequently, the read number, mapped read number, and expressed genes show decreasing trends during adventitious rooting.

To understand the gene expression profile during rooting, the clean reads were mapped back to the assembled unigenes using the BWA-0.6.2 software. The number of reads mapped to each unigene was then counted and normalized using RPKM [15]. Gene expression values were measured using the method described by DEGseq R package [38]. We identified a total of 11,717 unigenes that showed differential expression (fold change>2) during the adventitious root induction stage, whereas 12,737 unigenes showed differential expression during the adventitious root initiation stage. Between the induction stage and the initiation stage, 5,334 unigenes showed differential expression, suggesting their possible role in the activation of the primordium and root meristem formation. Using a DNA microarray method, Rigal et al. (2012) studied gene expression changes during adventitious rooting in the model tree *Populus trichocarpa*. Their results indicated that 5,781 genes were differentially expressed in the organization of the adventitious root primordium; 6,538 genes were differentially expressed during primordium differentiation; and 1,146 genes were differentially expressed between these two stages [9]. In another similar study using cDNA microarrays, Brinker et al. (2004) identified 220 genes that changed significantly during root development in hypocotyl cuttings of *Pinus contorta* [6]. The results obtained suggest that RNA-Seq is a sensitive, low-cost, and accurate method for deep-sequencing transcriptome of plant without available genomic information and was able to identify more DEGs during the early stages of adventitious rooting relative to the results of DNA microarrays. This technology also enables the precise elucidation of transcripts in the samples.

GO enrichment analysis indicated that the majority of GO categories significantly up-regulated at the root induction and initiation stages were protein synthesis-related, including ribosome, structural constituent of ribosome, translation, ribonucleoprotein complex, ribosomal subunit, cytosolic ribosome, non-membrane-bounded organelle, intracellular non-membrane-bounded organelle, and cytosolic part. Conversely, the significantly down-regulated GO categories were DNA, RNA synthesis-related, and signal transduction-related, which included DNA integration, RNA biosynthetic, nucleic acid metabolic process, nucleic acid binding transcription factor activity, sequence-specific DNA binding transcription factor activity. These results indicate that during the root induction stage, the cells experience an increase in the assembly of ribosomes and protein synthesis and a reduction of DNA and RNA synthesis [6]. GO categories related to response to stimulus and hormone signaling, such as response to chemical stimulus, oxidoreductase activity, response to endogenous stimulus, response to auxin stimulus, response to stimulus, response to hormone stimulus, and response to organic substance were significantly up-regulated at the root induction stage and down-regulated at the root initiation stage.

KEGG enrichment revealed that pathways such as ribosome, phenylpropanoid biosynthesis, phenylalanine metabolism, and terpenoid biosynthesis were up-regulated, whereas pathways such as photosynthesis, carbon fixation, carotenoid biosynthesis, nitrogen metabolism, sphingolipid metabolism, glycerolipid metabolism, cutin, cytokine-cytokine receptor interaction, oxidative phosphorylation, and plant hormone signal transduction were significantly down-regulated during the early stage of adventitious rooting. The loss of the photosynthetic function of hypocotyl cells was also revealed in hypocotyl cuttings of *P*. *contorta* at an early stage of adventitious root formation [6].

Although many genes specifically involved in the regulation of adventitious rooting have been identified in several plant species, the global profiling of gene expression during this process is not well studied using transcriptomic method. To better understand gene expression patterns during early root development, we selected the genes that exhibited greater than 32-fold changes and higher abundant expression (read number >1000). The most highly up-regulated unigenes encoded proteins involved in (1) functions related to stress, such as cationic peroxidase, pathogenesis-related protein, 7-ethoxycoumarin O-deethylase-like (cytochrome P450 monooxygenase), peroxidase C3-like isoform 2, low-temperature-induced 65 kDa protein-like (water stress-induced), early nodulin-like protein 1-like (phytocyanin family of blue copper proteins, a ubiquitous family of plant cupredoxins), late embryogenesis abundant protein (LEA-18, water stress-induced), heat shock 70 kDa protein-like; anthocyanin metabolism-associated and flavone metabolism-associated genes; (2) functions related to cell wall remodeling, such as polygalacturonase and polygalacturonase PG1 precursor (a pectin lyase-like superfamily protein), endoglucanase 17-like, peroxidase C3-like isoform 2 (lignin biosynthesis activity); (3) functions related to protein and lipid metabolism, such as ef1a, metacaspase-9-like (a peptidase), vignain-like (a peptidase), E3 ubiquitin-protein ligase HERC1-like, trypsin protease inhibitor precursor, and patatin group A-3-like (phospholipase A2 activity); (4) functions related to auxin transport and signal transduction, such as auxin efflux carrier, auxin-binding protein ABP19a-like, and proline-rich protein; and (5) a function act as transcription factors, such as ethylene-responsive transcription factor ERF086-like and the MYB family MYB114. In addition, the gene encoding casparian strip membrane protein 2 (unknown function) was specifically expressed in Wat24 compared with Wat6 (Table 14).

**Table 14:** Summary of the most differentially expressed genes during early stage of adventitious rooting in several plants investigated.

Plant material and treatment	Development stage of adventitious roots	Total genes identified	Differentially expressed genes	Most significantly up-regulated genes	Most significantly down-regulated genes
***Pinus contorta* hypocotyl cuttings treated with 1.23 mM IBA [** 6 **]**	root initiation and meristem formation	2,178	220 (fold change>2)	Cell replication: histone H3, CDC2. Cell wall weakening: cellulose, pectate lyase, endoxyloglucan transferase. Cell wall synthesis: arabinogalactan protin, peroxidase. Protein synthesis: ribosomal proteins. Protein assembly and folding: protein disulfide isomerase. Stress response: pathogenesis-related protein, late embryogenesis-abundant proteins. Auxin transport: ABC transporter, integral membrane transporter. Signal transduction: PINHEAD/ZWILLE-like protein, DNA binding protein, B-box zinc finger like protein. Protein degradation: ubiquitin-like proteins.	Photosynthesis: PS II protein, chloroplast proteins. Cell wall weakening: pectate lyase. Cell wall synthesis: arabinogalactan protin, caffeoyl-CoA-methyltransferase. Protein degradation: ubiquitin-like proteins. Stress response: late embryogenesis-abundant proteins, water stress inducible proteins. Flavonoid pathway: naringinin, 2-oxogluterate-3 dioxygenase, flavoprotein monooxygenase. Auxin signaling: auxin-repressed proteins, ABC transporter, AUX1-like, SAMS. Signal transduction: protein kinase PK-1. Transcription factors: EREBP.
***Camellia sinensis* L. single nodal cuttings treated with 0.4 mM IBA [** 46 **]**	24 h after treatment compared with the control	1,091	656 up-regulated and 435 down-regulated (fold change>2)	Plant hormone signaling: GH3, indole-3-acetate O-methyltransferase, cytokinin oxidases. Secondary metabolism: flavonoid biosynthesis, isoprenoid biosynthesis, 3-hydroxy-3-methylglutaryl-Co A reductase (mevalonate pathway). Cell wall modification: expansins, pectinesterase, cellulase, leucine-rich repeat extension, cellulose synthase. Transcription factor: bHLH135. Glutathione metabolism: glutathione synthetase, glutathione S-transferases.	Plant hormone signaling: adenylate isopentenyltransferase, cytokinin hydroxylases. Secondary metabolism: 2-C-methyl-d-erythritol 4-phosphate pathway, 1-deoxy-D-xylulose-5-phosphate synthase, 4-hydroxy-3-methylbut-2-enyl diphosphate reductase, geranyl pyrophosphate synthase.
***Populus trichocarpa* stem cuttings without auxin [** 9 **]**	root primordium organization and differentiation comparison with the dormant stage	55,970	7,107 (fold change>5)	Cell wall remodeling: glycoside hydrolases, pectate lyases, pectin esterases, expansins. Plant hormone signaling: auxin-, gibberellin-, ethylene-responsive genes. Signal transduction: Ser/Thr protein kinases. Transcription factors: lateral root primordium, AP2/ERF, MYB, NAC, WRKY, bHLH. AP2/ERF family: SCARECROW-like6, PISTILLATA, AINTEGUMENTA LIKE1, WRKY75.	
***Vigna radiata* L. seedling cuttings without auxin**	root induction compared with the control	78,697	11,717 (fold change>2), 2,945 up-regulated and 8,772 down-reglated	Stress response: peroxidase, pathogenesis-related protein, 7-ethoxycoumarin O-deethylase-like, low-temperature-induced 65 kDa protein-like, early nodulin-like protein 1-like (phytocyanin), late embryogenesis abundant protein. Flavonoid biosynthesis: anthocyanin metabolism-associated, flavone metabolism-associated. Cell wall remodeling: polygalacturonase (pectin lyase), endoglucanase 17-like, peroxidase, basic chitinase. Auxin signaling: auxin efflux carrier, auxin-binding protein ABP19a-like. Signal transduction: proline-rich protein. Transcription factors: ERF086-like, MYB114. Protein synthesis: ef1a. Protein degradation: metacaspase-9-like (a peptidase), vignain-like (a peptidase), trypsin protease inhibitor precursor. Lipid metabolism: patatin group A-3-like (phospholipase).	Protein degradation: E3 ubiquitin-protein ligase, polyprotein precursor (peptidase). Protein folding: heat shock 70 kDa protein-like. Auxin signaling: ABC transporter G family member 22-like, auxin-induced protein 5NG4-like. Amino metabolism: serine glyoxylate aminotransferase. Transporters: S-type anion channel SLAH3-like, organic cation transport protein. Signal transduction: histidine kinase. Transcription factor: MYB134.
	root initiation compared with the control	78,697	12,737, 3,434 up-regulated and 9,303 down- regulated	Stress response: peroxidase, pathogen-related protein, 7-ethoxycoumarin O-deethylase-like. Cell wall remodeling: polygalacturonase, basic chitinase. Protein degradation: trypsin protease inhibitor precursor. Lipid metabolism: patatin (phospholipase). Transcription factor: ERF086-like. Transporters: potassium transporter 5-like. Signal transduction: proline-rich protein. Unknown: casparian strip membrane protein.	Protein degradation: polyprotein precursor. Auxin signaling: auxin-induced protein 5NG4-like.

During the auxin-induced root initiation stage in hypocotyl cuttings of *Pinus contorta*, genes involved in cell replication and cell wall weakening and a transcript encoding a PINHEAD/ZWILLE-like protein were up-regulated, while genes related to auxin transport, photosynthesis, and cell wall synthesis were down-regulated. During the root meristem formation stage, the transcript abundance of genes involved in auxin transport, auxin responsive transcription, and cell wall synthesis, as well as a gene encoding a B-box zinc finger-like protein, was increased, while transcripts encoding proteins involved in cell wall weakening were decreased [6]. The most highly over-expressed transcripts during the induction stage of adventitious rooting in the stem cuttings of *Populus trichocarpa* were from genes that encoded proteins involved in cell wall remodeling, such as glycoside hydrolases (GHs), pectate lyases, pectin esterases and expansins, auxin-, gibberellin-, or ethylene-responsive genes, as well as genes that have been implicated in signaling, such as Ser/Thr protein kinases. The members of the AP2/ERF, MYB, NAC, WRKY, and bHLH transcription factor families exhibited significant expression changes during this process [9]. Table 14 summarizes the most differentially expressed genes during early stage of adventitious rooting with or without auxin treatment in several plant species investigated using DNA microarray or RNA-seq technologies. This summary indicates that, during early stage of adventitious rooting, the common gene functional categories occur in all plants investigated, including stress response, cell wall weakening and modification, plant hormone signaling, signal transduction, and transcription factors. Although the same genes appear in this list, the auxin-induced expression genes, even the plant-specific expression genes are also distinct. For example, genes encoding histone H3 and CDC2 associated with cell replication, genes encoding PINHEAD/ZWILLE-like protein, DNA binding protein, and B-box zinc finger like protein involved in signal transduction, were induced by IBA; and genes encoding AUX1-like and SAMS were repressed by IBA in *Pinus contorta* [6]. Genes encoding GH3, indole-3-acetate O-methyltransferase, and cytokinin oxidases involved in plant hormone signaling, and gene encoding glutathione S-transferases (GSTs) in glutathione synthesis, were induced by IBA; and genes encoding adenylate isopentenyltransferase and cytokinin hydroxylases involved in plant hormone signaling were down-regulated by IBA in *Camellia sinensis* [46]. Genes encoding lateral root primordium (*lrp1*), SCARECROW-like6, PISTILLATA, and AINTEGUMENTA LIKE1 (PtAIL1, PtPLT1.1, and PtAIL9) in AP2/ERF transcription factor family, were up-regulated in *Populus trichocarpa* without auxin treatment [9]. Genes encoding metacaspase-9-like (a peptidase), vignain-like (a peptidase), and trypsin protease inhibitor precursor involved in protein degradation, gene encoding patatin group A-3-like (a phospholipase) involved in lipid metabolism, and genes encoding potassium transporter 5-like related to transporter, were up-regulated; and genes encoding S-type anion channel SLAH3-like and organic cation transport protein functioning as transporters were down-regulated in *Vigna radiata* in this study.

The profile of the top up-regulated genes indicates that stress-response processes are paramount during the early stage of adventitious rooting. The excision of primary roots, acting as a type of wounding stress, causes an oxidative burst leading to oxidative stress during adventitious rooting in mung bean seedlings [16, 18, 23–25]. Many studies have shown that in the course of adventitious rooting, peroxidase (POD) activity is sharply reduced during the induction stage, increased during the initiation stage and gradually reduced during the expression stage [16, 23, 47, 48]. Peroxidases comprise a large family of enzymes that function as antioxidants and also respond to water stress [49]. Late embryogenesis abundant (LEA) proteins act as water-binding molecules, membrane-stabilizers, and ion modulators and are induced by drought stress [49–52]. Increases in LEA proteins and pathogenesis-related proteins indicate that the plants were exposed to water stress [6]. During the root primordia formation stage in *P*. *contorta*, transcripts encoding enzymes of the flavonoid pathway were up-regulated [6]. One of these flavonoid pathway proteins, chalcone synthase, and a pathogenesis-related protein contribute to a constitutive defense barrier in the root epidermis of the pea [53]. Increases in the expression of isoflavone reductase-like and isoflavone 2'-hydroxylase-like genes suggests a role for the flavonoid pathway in response to stress during early stages of rooting.

The regulation of genes with potential roles in cell wall remodeling is an essential process for adventitious root formation. In this study, the top up-regulated genes included many that encode proteins involved in cell wall synthesis and loosening, such as the significantly up-regulated genes endoglucanase 17-like and endo-1,3(4)-beta-glucanases, which are members of the glycoside hydrolase family and potentially participate in cell wall loosening [54]. The phenylpropanoid biosynthesis and phenylalanine metabolism pathways were also significantly up-regulated. The phenylpropanoid polymer complex is the component of lignin that accumulates between cellulose, hemicellulose and pectin components in the cell wall. Phenylpropanoid synthesis starts from phenylalanine [55]. The common derivatives from phenylpropanoid pathway, phenolic acids, flavonoids, and lignin [56], are crucial regulators in cell division and differentiation [57] and stimulate in vitro rooting [58]. During the early stage of adventitious rooting, genes with the potential to be active in cell wall synthesis were down-regulated, while genes involved in weakening cell walls were up-regulated, suggesting that the cell walls were undergoing remodeling and weakening [6, 9].

Genes associated with auxin transport and signaling are regulated during the early stage of adventitious rooting. During the root induction stage, the gene encoding auxin efflux carrier was significantly up-regulated. Auxin efflux carriers control auxin distribution to establish and maintain auxin concentration gradients in various tissues [59], triggering the establishment of new growth axes [60]. During the root initiation stage, an auxin signaling gene, auxin-binding protein ABP19a-like, was specifically expressed. ABP1 has known to mediate rapid cellular auxin effects through the non-transcriptional auxin response pathway and is essential for auxin-regulated processes. ABP1 also regulates the expression of AUX/IAA genes [61, 62], suggesting that active transport of auxin starts during the early stage of root meristem formation. However, down-regulation was observed in another auxin carrier complex gene, ABC transporter G family member 22-like, which functions in cellular auxin efflux and influx [63].

In this study, the gene encoding the ethylene-responsive transcription factor ERF017-like was significantly down-regulated during the root initiation stage. During root primordia formation in *P*. *contorta* cuttings, the gene encoding an ethylene responsive element binding protein (EREBP)-like protein was down-regulated [6]. Ethylene biosynthesis has been demonstrated to be required for adventitious root formation, and there is crosstalk between ethylene and auxin during the process of adventitious root formation in tomato [64, 65]. These results suggest that ethylene signaling also mediates adventitious rooting.

The MYB transcription factor is an important regulator of the initiation and primordium formation of adventitious roots. Interestingly, three genes encoding the MYB transcription factor MYB134 were significantly down-regulated during the root induction stage but significantly up-regulated during the root initiation stage (S7 Table and Fig 6) in this study. In cuttings of *Populus trichocarpa*, MYB family expression levels changed the most during primordium differentiation [9]. MYB77 acts in a synergistic manner with ARF7 to enhance the expression of auxin-responsive genes and mediate the auxin response [66]. These results suggest that members of the MYB transcription factor family mediate the initiation of and the formation of adventitious root primordium.

## Conclusions

In recent years, we have made great efforts to reveal the mechanisms that regulate adventitious rooting in plants at the physiological and molecular levels using a model plant *Vigna radiata* (L.) R. Wilczek, a tropical legume that serves as a significant source of dietary protein for the people of Asia and Africa. In this paper, we provide the first study to report the transcriptome of seedling hypocotyls of *V*. *radiata* and *in vitro* adventitious rooting of hypocotyl cuttings using RNA-Seq analysis. We obtained 78,697 assembled unigenes using the Trinity *de novo* assembly method. Among these unigenes, 72,342, 66,663, and 64,680 genes were expressed in hypocotyls or at the 6 h and 24 h time points during adventitious rooting, respectively, but only 29,029 (36.77%) unigenes could be annotated using public databases. The global transcriptomic data reveal that profound cellular and metabolic reorganization occurs during the root induction stage. We used gene clustering and the enrichment of GO terms and KEGG pathways to describe the overall biological processes regulated during this developmental process. We also used RPKM analysis to investigate the differentially expressed gene profiles at the three developmental stages. Furthermore, real-time quantitative PCR was used to confirm the differential expression levels observed for 39 of the unigenes. The results obtained using RNA-Seq were consistent with the average expression levels in three biological replicates. Further investigation of the transcriptional changes at more closely spaced developmental stages will provide additional valuable information. Our full transcript abundance analysis, presented in S2 and S7 Tables, represents a useful resource for further insight into mung bean transcriptome and *in vitro* adventitious root development and for candidate gene selection.

## Supporting Information

S1 Fig GC content distribution of unigenes. (TIF)

S2 Fig Length distribution of unigenes. (TIF)

S3 Fig Venn diagram of number of unigenes annotated by BLASTx with an E-value threshold of 10^−5^ against protein databases. The numbers in the circles indicate the number of unigenes annotated by single or multiple databases. The Venn diagram shows unigenes unique to each database and which are shared amongst different databases.
(TIF)

S4 Fig Percentage of unigenes matching the 10 top species using BLASTx against the Nr database. (TIF)

S1 Table Statistics of random 100,000 sequences alignment against Nr database. (PDF)

S2 Table BLAST results against the NCBI Nr database for all the assembled unigenes with an E-value threshold of 1e-5. (XLSX)

S3 Table Top 10 significant GOs in the three samples. (PDF)

S4 Table Top list of significantly up- and down-regulated GOs. (PDF)

S5 Table Top list of significantly up- and down-regulated KOs. (PDF)

S6 Table Top list of significantly up- and down-regulated DEGs in the three samples. (PDF)

S7 Table The expression abundance of unigenes in the three samples presented as read number and RPKM. (XLSX)

S8 Table List of the genes and primers selected for q-PCR validation. (PDF)
