# Supplementary figures and images for: De Novo Characterization of the Mung Bean Transcriptome and Transcriptomic Analysis of Adventitious Rooting in Seedlings Using RNA-Seq
Source: PLoS One. 2015 Jul 15;10(7):e0132969. doi: 10.1371/journal.pone.0132969 (PMC4503682; doi:10.1371/journal.pone.0132969)

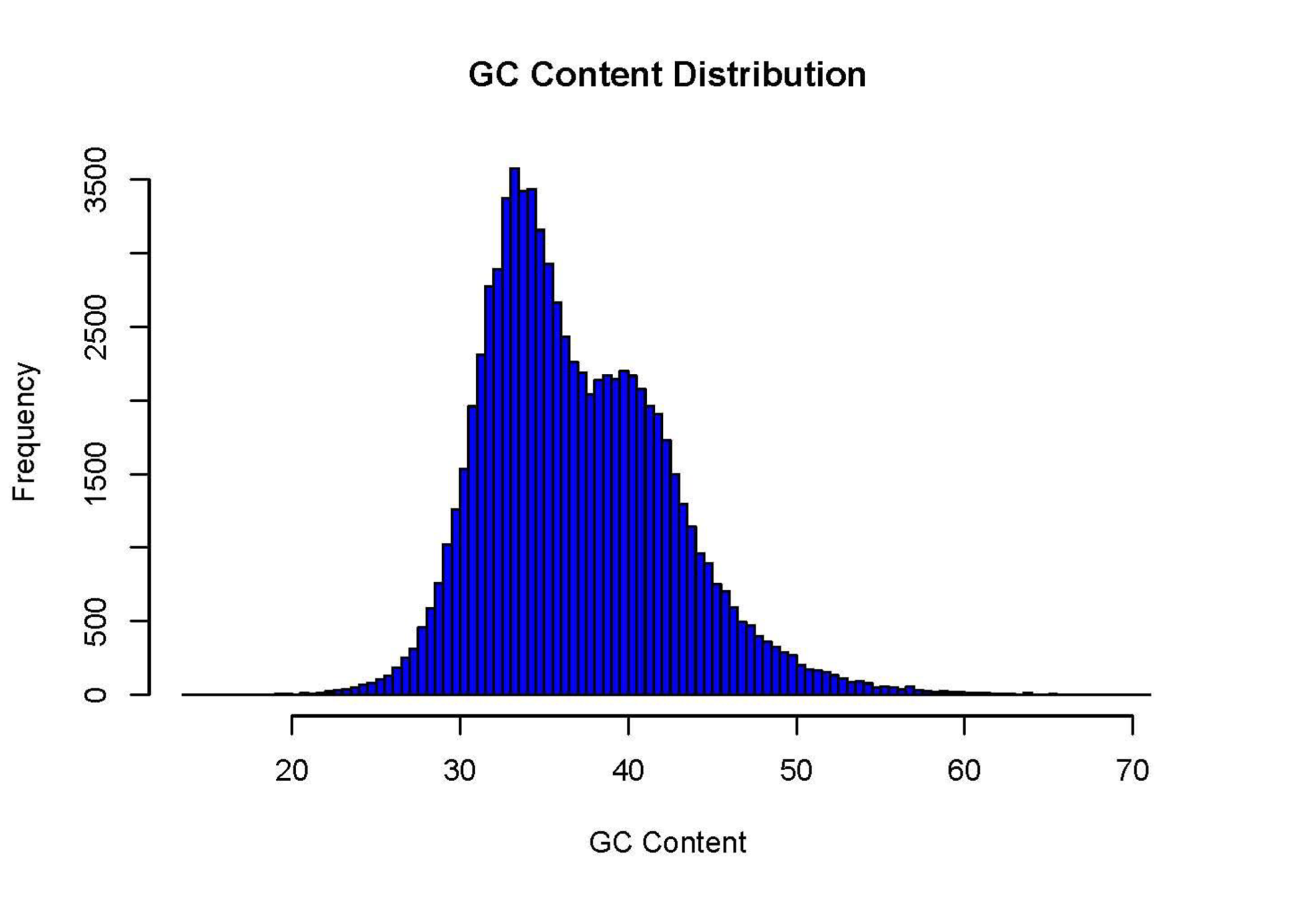

Supplement: S1 Fig — (TIF) [file pone.0132969.s001.tif]

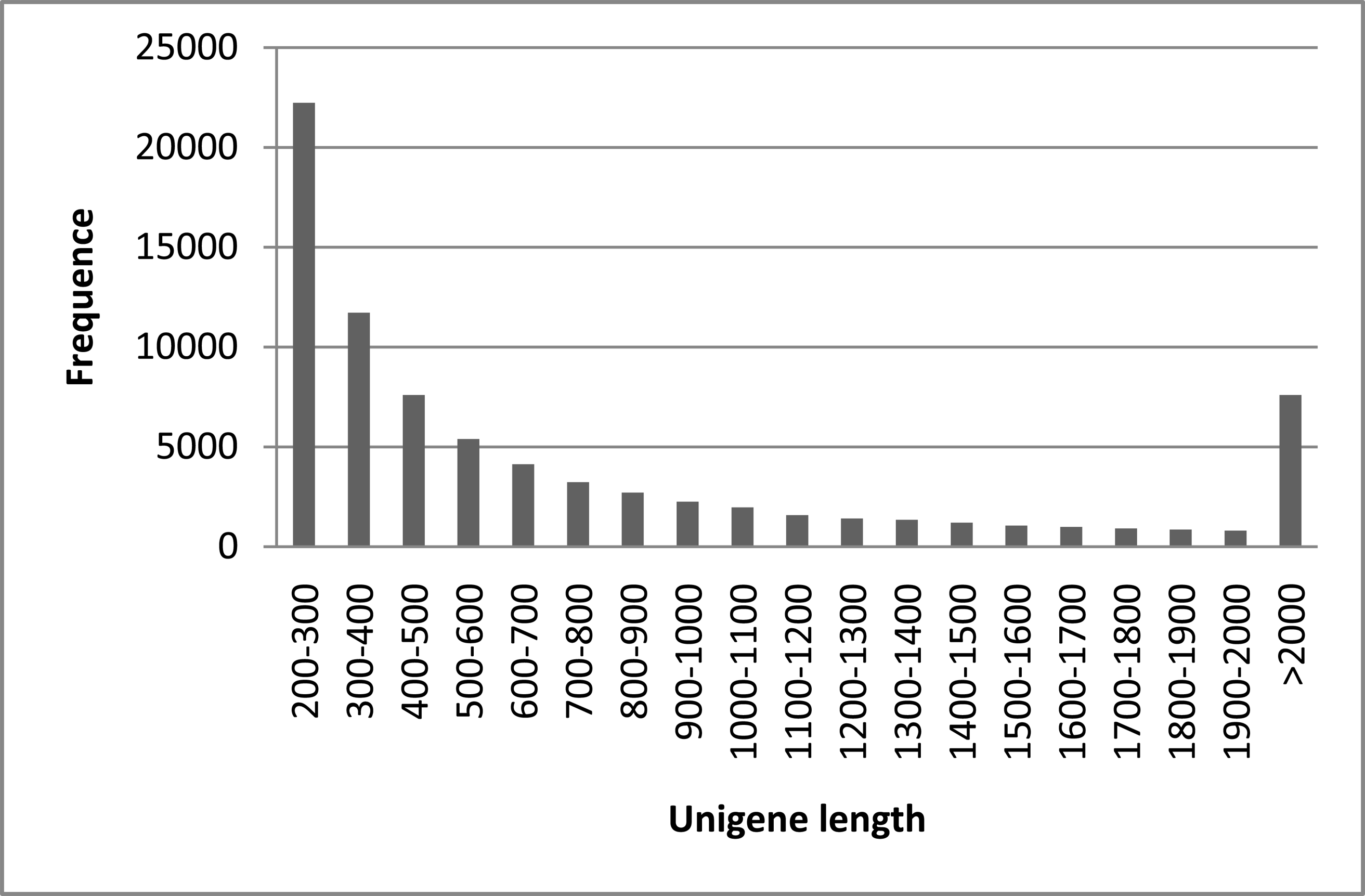

Supplement: S2 Fig — (TIF) [file pone.0132969.s002.tif]

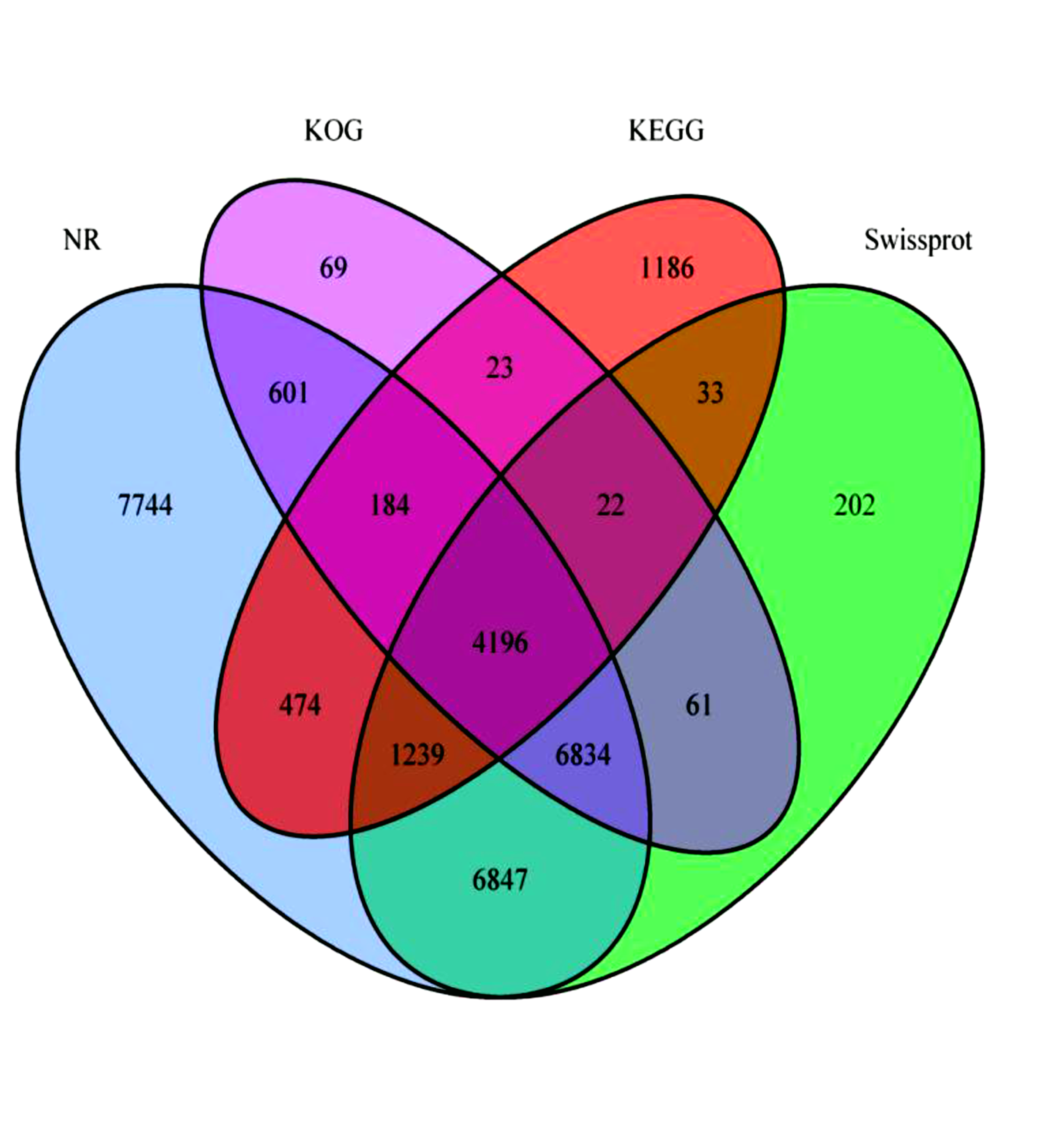

Supplement: S3 Fig — The numbers in the circles indicate the number of unigenes annotated by single or multiple databases. The Venn diagram shows unigenes unique to each database and which are shared amongst different databases. (TIF) [file pone.0132969.s003.tif]

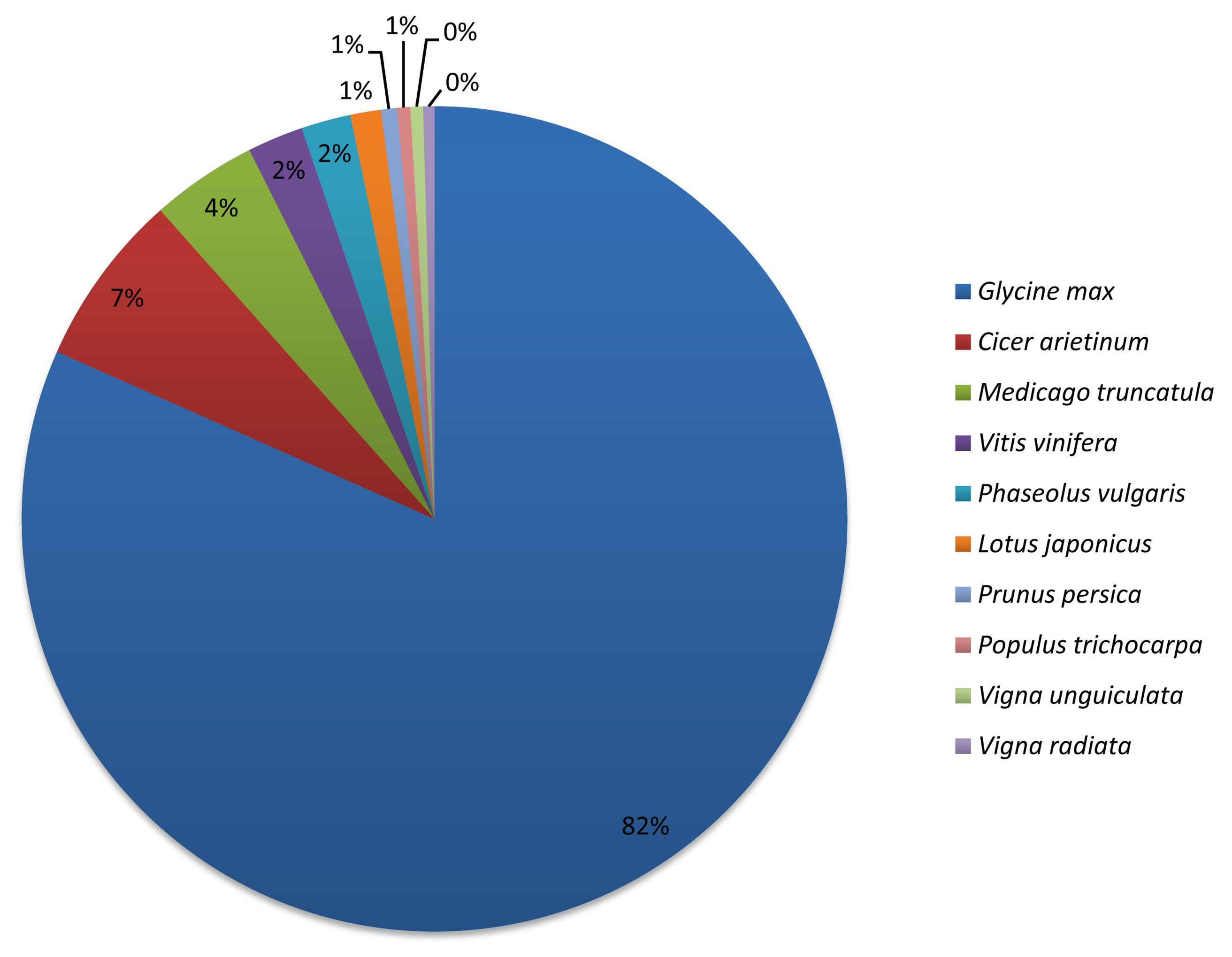

Supplement: S4 Fig — (TIF) [file pone.0132969.s004.tif]
