# Supplementary material for: De Novo Characterization of the Mung Bean Transcriptome and Transcriptomic Analysis of Adventitious Rooting in Seedlings Using RNA-Seq
Source: PLoS One. 2015 Jul 15;10(7):e0132969. doi: 10.1371/journal.pone.0132969 (PMC4503682; doi:10.1371/journal.pone.0132969)
Supplement: S1 Table — (PDF) [file pone.0132969.s005.pdf]

S1Table. Statistics of random 100,000 sequences alignment against Nr database

| Con. | | Wat6 | | Wat24 | |
|---|---|---|---|---|---|
| *Glycine max* | 54716 | *Glycine max* | 54448 | *Glycine max* | 56376 |
| *Phaseolus vulgaris* | 4240 | *Phaseolus vulgaris* | 4483 | *Phaseolus vulgaris* | 5312 |
| *Vigna radiata* | 4027 | *Vigna radiata* | 3913 | *Vigna radiata* | 3807 |
| *Cicer arietinum* | 2056 | *Cicer arietinum* | 2344 | *Cicer arietinum* | 2214 |
| *Lotus japonicus* | 1366 | *Lotus japonicus* | 1499 | *Lotus japonicus* | 1598 |
| *Medicago truncatula* | 1125 | *Vigna unguiculata* | 1170 | *Medicago truncatula* | 1152 |
| *Vigna unguiculata* | 927 | *Medicago truncatula* | 1129 | *Vigna unguiculata* | 1022 |
| *Vigna radiata var. radiata* | 509 | *Vigna radiata var. radiata* | 657 | *Vigna radiata var. radiata* | 504 |
| *Vigna angularis* | 455 | *Vigna angularis* | 506 | *Vigna angularis* | 495 |
| *Phaseolus lunatus* | 231 | *Phaseolus lunatus* | 409 | *Phaseolus lunatus* | 329 |
| *Vigna mungo* | 200 | *Vigna mungo* | 262 | *Vigna mungo* | 309 |
| *Glycine tomentella* | 155 | *Glycine tomentella* | 154 | *Vigna aconitifolia* | 182 |
| *Fragaria vesca subsp. vesca* | 101 | *Cajanus cajan* | 131 | *Fragaria vesca subsp. vesca* | 146 |
| *Cajanus cajan* | 98 | *Pueraria montana var. lobata* | 124 | *Cajanus cajan* | 135 |
| *Pisum sativum* | 91 | *Fragaria vesca subsp. vesca* | 123 | *Pueraria montana var. lobata* | 108 |
| *Solanum lycopersicum* | 81 | *Vigna aconitifolia* | 102 | *Pisum sativum* | 96 |
| *Vitis vinifera* | 68 | *Pisum sativum* | 94 | *Glycine tomentella* | 78 |
| *Lablab purpureus* | 68 | *Lablab purpureus* | 89 | *Phaseolus acutifolius* | 71 |
| *Vigna aconitifolia* | 66 | *Cloning vector pFosill-2* | 73 | *Solanum lycopersicum* | 63 |
| *Cucumis sativus* | 64 | *Vitis vinifera* | 72 | *Lablab purpureus* | 59 |
| *Phaseolus acutifolius* | 63 | *Phaseolus acutifolius* | 71 | *Populus trichocarpa* | 59 |
