# Supplementary material for: De Novo Characterization of the Mung Bean Transcriptome and Transcriptomic Analysis of Adventitious Rooting in Seedlings Using RNA-Seq
Source: PLoS One. 2015 Jul 15;10(7):e0132969. doi: 10.1371/journal.pone.0132969 (PMC4503682; doi:10.1371/journal.pone.0132969)
Supplement: S3 Table — (PDF) [file pone.0132969.s007.pdf]

S3 Table. Top 10 significant GOs in the three samples

| GO ID | Total Gene num | DEGs num | FDR | Level | Terms |
|---|---|---|---|---|---|
| Wat6 | | | | | |
| GO:0016491 | 1856 | 571 | 1.81E-14 | 3 | oxidoreductase activity |
| GO:1901700 | 1372 | 428 | 2.33E-11 | 4 | response to oxygen-containing compound |
| GO:0005576 | 1222 | 387 | 2.98E-11 | 2 | extracellular region |
| GO:0001071 | 1345 | 417 | 6.13E-11 | 2 | nucleic acid binding transcription factor activity |
| GO:0003700 | 1345 | 417 | 6.13E-11 | 3 | sequence-specific DNA binding transcription factor activity |
| GO:0015979 | 192 | 86 | 5.45E-09 | 4 | photosynthesis |
| GO:0042221 | 2287 | 643 | 1.21E-08 | 3 | response to chemical stimulus |
| GO:0009637 | 73 | 43 | 1.60E-08 | 6 | response to blue light |
| GO:0006979 | 368 | 138 | 2.59E-08 | 4 | response to oxidative stress |
| GO:0046906 | 485 | 171 | 4.00E-08 | 4 | tetrapyrrole binding |
| Wat24 | | | | | |
| GO:0003735 | 471 | 283 | 1.37E-75 | 3 | structural constituent of ribosome |
| GO:0005840 | 538 | 307 | 6.59E-75 | 4 | ribosome |
| GO:0005198 | 606 | 304 | 5.99E-57 | 2 | structural molecule activity |
| GO:0044391 | 216 | 147 | 3.23E-48 | 4 | ribosomal subunit |
| GO:0022626 | 198 | 135 | 2.37E-44 | 5 | cytosolic ribosome |
| GO:0006412 | 671 | 301 | 1.24E-43 | 6 | translation |
| GO:0030529 | 801 | 328 | 1.31E-37 | 3 | ribonucleoprotein complex |
| GO:0044445 | 243 | 139 | 3.99E-33 | 5 | cytosolic part |
| GO:0022625 | 96 | 74 | 1.64E-29 | 6 | cytosolic large ribosomal subunit |
| GO:0015934 | 125 | 85 | 2.00E-27 | 5 | large ribosomal subunit |
| GO:0005576 | 1222 | 415 | 2.65E-26 | 2 | extracellular region |
| Wat24:Wat6 | | | | | |
| GO:0005840 | 538 | 175 | 1.03E-26 | 4 | ribosome |
| GO:0003735 | 471 | 156 | 8.92E-25 | 3 | structural constituent of ribosome |
| GO:0005198 | 606 | 175 | 1.68E-20 | 2 | structural molecule activity |
| GO:0006412 | 671 | 176 | 6.93E-16 | 6 | translation |
| GO:0005576 | 1222 | 272 | 7.80E-15 | 2 | extracellular region |
| GO:0044391 | 216 | 77 | 1.19E-13 | 4 | ribosomal subunit |
| GO:0046906 | 485 | 133 | 1.75E-13 | 4 | tetrapyrrole binding |
| GO:0016491 | 1856 | 372 | 1.75E-13 | 3 | oxidoreductase activity |
| GO:0020037 | 452 | 125 | 6.13E-13 | 5 | heme binding |
| GO:0022626 | 198 | 71 | 7.60E-13 | 5 | cytosolic ribosome |
