# Supplementary material for: De Novo Characterization of the Mung Bean Transcriptome and Transcriptomic Analysis of Adventitious Rooting in Seedlings Using RNA-Seq
Source: PLoS One. 2015 Jul 15;10(7):e0132969. doi: 10.1371/journal.pone.0132969 (PMC4503682; doi:10.1371/journal.pone.0132969)
Supplement: S4 Table — (PDF) [file pone.0132969.s008.pdf]

S4 Table. Top list of significant up- and down-regulated GOs

Top dif GO enrichment-wat6:con-up

GO ID	All num	DEGs num	P-value	FDR	Level	Term	Type
GO:0005198	606	152	2.73E-32	4.09E-29	2	structural molecule activity	molecular_function
GO:0005576	1222	221	1.31E-24	6.56E-22	2	extracellular region	cellular_component
GO:0044421	174	48	1.16E-12	1.86E-10	2	extracellular region part	cellular_component
GO:0009055	462	85	1.79E-10	2.30E-08	2	electron carrier activity	molecular_function
GO:0016209	188	46	2.90E-10	3.44E-08	2	antioxidant activity	molecular_function
GO:0005215	1416	188	3.41E-08	2.33E-06	2	transporter activity	molecular_function
GO:0030054	727	109	9.11E-08	5.55E-06	2	cell junction	cellular_component
GO:0055044	694	105	9.95E-08	5.82E-06	2	symplast	cellular_component
GO:0045735	76	18	0.000116	0.002727	2	nutrient reservoir activity	molecular_function
GO:0003735	471	140	1.82E-38	8.19E-35	3	structural constituent of ribosome	molecular_function
GO:0016491	1856	320	6.30E-32	7.09E-29	3	oxidoreductase activity	molecular_function
GO:0030529	801	161	8.70E-23	3.92E-20	3	ribonucleoprotein complex	cellular_component
GO:0005615	141	47	7.34E-16	2.36E-13	3	extracellular space	cellular_component
GO:0030312	671	120	2.27E-13	5.12E-11	3	external encapsulating structure	cellular_component
GO:0044710	3304	412	4.60E-13	9.02E-11	3	single-organism metabolic process	biological_process
GO:0048037	658	117	6.84E-13	1.14E-10	3	cofactor binding	molecular_function
GO:0042221	2287	293	1.44E-10	1.97E-08	3	response to chemical stimulus	biological_process
GO:0004601	164	42	3.78E-10	4.26E-08	3	peroxidase activity	molecular_function
GO:0051240	97	29	4.39E-09	3.88E-07	3	positive regulation of multicellular organismal process	biological_process
GO:0004857	161	38	2.85E-08	2.00E-06	3	enzyme inhibitor activity	molecular_function
GO:0061134	58	20	7.80E-08	4.88E-06	3	peptidase regulator activity	molecular_function
GO:0005911	702	106	9.57E-08	5.75E-06	3	cell-cell junction	cellular_component
GO:0009506	694	105	9.95E-08	5.82E-06	3	plasmodesma	cellular_component
GO:0070469	113	28	6.40E-07	3.14E-05	3	respiratory chain	cellular_component
GO:0022892	1051	142	6.69E-07	3.20E-05	3	substrate-specific transporter activity	molecular_function
GO:0044765	1550	192	3.14E-06	0.000135	3	single-organism transport	biological_process
GO:0043228	1628	200	3.35E-06	0.00014	3	non-membrane-bounded organelle	cellular_component
GO:0034358	13	8	3.76E-06	0.000156	3	plasma lipoprotein particle	cellular_component
GO:0032994	14	8	8.08E-06	0.000303	3	protein-lipid complex	cellular_component
GO:0016829	359	58	1.02E-05	0.000374	3	lyase activity	molecular_function
GO:0022857	1145	146	1.16E-05	0.000393	3	transmembrane transporter activity	molecular_function
GO:1901681	37	13	1.17E-05	0.000393	3	sulfur compound binding	molecular_function
GO:0009719	1364	166	3.99E-05	0.001159	3	response to endogenous stimulus	biological_process
GO:0071944	3429	372	4.83E-05	0.001352	3	cell periphery	cellular_component
GO:0031224	4742	497	6.75E-05	0.001779	3	intrinsic to membrane	cellular_component
GO:0005577	7	5	0.000108	0.002586	3	fibrinogen complex	cellular_component
GO:0097367	40	12	0.000147	0.00334	3	carbohydrate derivative binding	molecular_function

Top 50 dif GO enrichment-wat6:con-up

GO ID	All num	DEGs num	P-value	FDR	Level	Term	Type
GO:0003735	471	140	1.82E-38	8.19E-35	3	structural constituent of ribosome	molecular_function
GO:0005840	538	149	6.13E-37	1.38E-33	4	ribosome	cellular_component
GO:0005198	606	152	2.73E-32	4.09E-29	2	structural molecule activity	molecular_function
GO:0016491	1856	320	6.30E-32	7.09E-29	3	oxidoreductase activity	molecular_function
GO:0022626	198	77	3.43E-30	3.09E-27	5	cytosolic ribosome	cellular_component
GO:0044445	243	82	4.49E-27	3.37E-24	5	cytosolic part	cellular_component

GO:0006412	671	151	1.25E-26	8.03E-24	6	translation	biological_process
GO:0044391	216	76	1.65E-26	9.29E-24	4	ribosomal subunit	cellular_component
GO:0005576	1222	221	1.31E-24	6.56E-22	2	extracellular region	cellular_component
GO:0030529	801	161	8.70E-23	3.92E-20	3	ribonucleoprotein complex	cellular_component
GO:0020037	452	103	6.93E-19	2.84E-16	5	heme binding	molecular_function
GO:0022625	96	40	1.36E-17	5.10E-15	6	cytosolic large ribosomal subunit	cellular_component
GO:0046906	485	105	1.54E-17	5.33E-15	4	tetrapyrrole binding	molecular_function
GO:0005615	141	47	7.34E-16	2.36E-13	3	extracellular space	cellular_component
GO:0000302	241	62	2.10E-14	6.31E-12	5	response to reactive oxygen species	biological_process
GO:0015935	91	35	2.66E-14	7.48E-12	5	small ribosomal subunit	cellular_component
GO:0005618	654	120	3.54E-14	9.38E-12	4	cell wall	cellular_component
GO:0006979	368	80	9.08E-14	2.25E-11	4	response to oxidative stress	biological_process
GO:0015934	125	41	9.50E-14	2.25E-11	5	large ribosomal subunit	cellular_component
GO:0030312	671	120	2.27E-13	5.12E-11	3	external encapsulating structure	cellular_component
GO:0043436	1141	177	3.54E-13	7.59E-11	5	oxoacid metabolic process	biological_process
GO:0042542	164	47	4.34E-13	8.89E-11	5	response to hydrogen peroxide	biological_process
GO:0044710	3304	412	4.60E-13	9.02E-11	3	single-organism metabolic process	biological_process
GO:0006082	1147	177	5.66E-13	1.05E-10	4	organic acid metabolic process	biological_process
GO:0005506	489	95	5.81E-13	1.05E-10	7	iron ion binding	molecular_function
GO:0022627	62	27	6.84E-13	1.14E-10	6	cytosolic small ribosomal subunit	cellular_component
GO:0048037	658	117	6.84E-13	1.14E-10	3	cofactor binding	molecular_function
GO:0044421	174	48	1.16E-12	1.86E-10	2	extracellular region part	cellular_component
GO:0044711	911	147	2.03E-12	3.15E-10	4	single-organism biosynthetic process	biological_process
GO:0044444	6765	746	7.32E-12	1.10E-09	4	cytoplasmic part	cellular_component
GO:0019752	1091	163	6.73E-11	9.79E-09	6	carboxylic acid metabolic process	biological_process
GO:1901605	361	72	1.10E-10	1.55E-08	5	alpha-amino acid metabolic process	biological_process
GO:0042221	2287	293	1.44E-10	1.97E-08	3	response to chemical stimulus	biological_process
GO:0015291	349	70	1.54E-10	2.04E-08	5	secondary active transmembrane transporter activity	molecular_function
GO:0009055	462	85	1.79E-10	2.30E-08	2	electron carrier activity	molecular_function
GO:0042743	131	37	2.08E-10	2.61E-08	5	hydrogen peroxide metabolic process	biological_process
GO:0050662	473	86	2.59E-10	3.15E-08	4	coenzyme binding	molecular_function
GO:0016209	188	46	2.90E-10	3.44E-08	2	antioxidant activity	molecular_function
GO:0004601	164	42	3.78E-10	4.26E-08	3	peroxidase activity	molecular_function
GO:0016684	164	42	3.78E-10	4.26E-08	4	oxidoreductase activity, acting on peroxide as acceptor	molecular_function
GO:0010378	9	9	4.00E-10	4.39E-08	4	temperature compensation of the circadian clock	biological_process
GO:0044283	769	122	4.93E-10	5.29E-08	5	small molecule biosynthetic process	biological_process
GO:0034614	141	38	5.30E-10	5.55E-08	6	cellular response to reactive oxygen species	biological_process
GO:0034599	148	39	6.47E-10	6.62E-08	5	cellular response to oxidative stress	biological_process
GO:0016053	651	107	7.97E-10	7.81E-08	5	organic acid biosynthetic process	biological_process
GO:0046394	651	107	7.97E-10	7.81E-08	6	carboxylic acid biosynthetic process	biological_process
GO:0042744	114	33	9.72E-10	9.32E-08	5	hydrogen peroxide catabolic process	biological_process
GO:0070301	121	34	1.29E-09	1.21E-07	6	cellular response to hydrogen peroxide	biological_process
GO:0005739	1317	182	2.93E-09	2.70E-07	5	mitochondrion	cellular_component
GO:0055114	563	94	3.86E-09	3.48E-07	4	oxidation-reduction process	biological_process

Top list dif GO enrichment-wat6:con-down

GO ID	All num	DEGs num	P-value	FDR	Level	Term	Type
GO:0001071	1345	330	4.60E-30	1.08E-26	2	nucleic acid binding transcription factor activity	molecular_function

GO:0005488	12800	1821	1.14E-05	0.000505	2	binding	molecular_function
GO:0050789	4100	634	1.46E-05	0.000618	2	regulation of biological process	biological_process
GO:0050896	5231	791	2.23E-05	0.000877	2	response to stimulus	biological_process
GO:0065007	4447	680	2.76E-05	0.001051	2	biological regulation	biological_process
GO:0023052	1715	278	0.000319	0.008585	2	signaling	biological_process
GO:0003700	1345	330	4.60E-30	1.08E-26	3	sequence-specific DNA binding transcription factor activity	molecular_function
GO:0034357	276	87	4.58E-15	1.43E-12	3	photosynthetic membrane	cellular_component
GO:0009521	79	38	1.03E-13	2.20E-11	3	photosystem	cellular_component
GO:0009628	1566	291	1.53E-09	1.40E-07	3	response to abiotic stimulus	biological_process
GO:1901363	9640	1422	1.30E-07	9.24E-06	3	heterocyclic compound binding	molecular_function
GO:0019222	2140	367	1.36E-07	9.43E-06	3	regulation of metabolic process	biological_process
GO:0097159	9651	1422	1.79E-07	1.21E-05	3	organic cyclic compound binding	molecular_function
GO:0003682	340	79	5.24E-07	3.32E-05	3	chromatin binding	molecular_function
GO:0050794	3519	561	1.42E-06	7.90E-05	3	regulation of cellular process	biological_process
GO:0009791	1125	201	9.09E-06	0.000413	3	post-embryonic development	biological_process
GO:0051656	63	22	1.23E-05	0.000534	3	establishment of organelle localization	biological_process
GO:0009654	17	10	1.48E-05	0.000618	3	oxygen evolving complex	cellular_component
GO:0042754	5	5	4.35E-05	0.001604	3	negative regulation of circadian rhythm	biological_process
GO:0006807	6258	929	5.30E-05	0.001912	3	nitrogen compound metabolic process	biological_process
GO:0009719	1364	231	7.82E-05	0.002635	3	response to endogenous stimulus	biological_process
GO:0010166	28	12	0.000126	0.003907	3	wax metabolic process	biological_process
GO:0019898	37	14	0.000175	0.005136	3	extrinsic to membrane	cellular_component
GO:0044700	1710	276	0.000441	0.011169	3	single organism signaling	biological_process
GO:0006950	3162	484	0.000498	0.012404	3	response to stress	biological_process
GO:0010073	95	25	0.000592	0.0146	3	meristem maintenance	biological_process

Top 50 dif GO enrichment-wat6:con-down

GO ID	All num	DEGs num	P-value	FDR	Level	Term	Type
GO:0001071	1345	330	4.60E-30	1.08E-26	2	nucleic acid binding transcription factor activity	molecular_function
GO:0003700	1345	330	4.60E-30	1.08E-26	3	sequence-specific DNA binding transcription factor activity	molecular_function
GO:0032774	2282	480	1.05E-26	1.24E-23	6	RNA biosynthetic process	biological_process
GO:0006351	2276	479	1.06E-26	1.24E-23	6	transcription, DNA-dependent	biological_process
GO:0044271	2774	550	3.01E-24	2.82E-21	5	cellular nitrogen compound biosynthetic process	biological_process
GO:0034654	2580	511	2.65E-22	2.07E-19	5	nucleobase-containing compound biosynthetic process	biological_process
GO:0018130	2786	542	6.49E-22	4.34E-19	5	heterocycle biosynthetic process	biological_process
GO:0003677	2302	460	9.48E-21	5.56E-18	5	DNA binding	molecular_function
GO:0019438	2870	549	1.60E-20	8.31E-18	5	aromatic compound biosynthetic process	biological_process
GO:0015979	192	77	2.82E-20	1.32E-17	4	photosynthesis	biological_process
GO:1901362	2982	561	2.36E-19	1.00E-16	5	organic cyclic compound biosynthetic process	biological_process
GO:0044436	299	93	1.33E-15	5.20E-13	4	thylakoid part	cellular_component
GO:0009579	383	110	1.86E-15	6.69E-13	4	thylakoid	cellular_component
GO:0016070	2943	534	3.52E-15	1.18E-12	5	RNA metabolic process	biological_process
GO:0034357	276	87	4.58E-15	1.43E-12	3	photosynthetic membrane	cellular_component
GO:0044434	960	214	1.05E-14	3.07E-12	5	chloroplast part	cellular_component
GO:0044435	972	215	2.11E-14	5.83E-12	4	plastid part	cellular_component
GO:0031976	312	93	2.43E-14	6.00E-12	5	plastid thylakoid	cellular_component
GO:0009534	312	93	2.43E-14	6.00E-12	6	chloroplast thylakoid	cellular_component
GO:0006355	1265	264	3.01E-14	6.72E-12	6	regulation of transcription, DNA-dependent	biological_process

GO:2001141	1265	264	3.01E-14	6.72E-12	6	regulation of RNA biosynthetic process	biological_process
GO:0009521	79	38	1.03E-13	2.20E-11	3	photosystem	cellular_component
GO:0051252	1292	266	1.24E-13	2.53E-11	5	regulation of RNA metabolic process	biological_process
GO:0009416	589	144	2.02E-13	3.95E-11	5	response to light stimulus	biological_process
GO:0042651	262	80	3.85E-13	7.22E-11	4	thylakoid membrane	cellular_component
GO:0009314	617	147	9.80E-13	1.77E-10	4	response to radiation	biological_process
GO:0055035	250	76	1.90E-12	3.18E-10	5	plastid thylakoid membrane	cellular_component
GO:0009535	250	76	1.90E-12	3.18E-10	6	chloroplast thylakoid membrane	cellular_component
GO:0009507	1876	353	4.00E-12	6.46E-10	6	chloroplast	cellular_component
GO:0018298	59	30	6.59E-12	1.03E-09	7	protein-chromophore linkage	biological_process
GO:0031984	349	94	1.19E-11	1.80E-09	4	organelle subcompartment	cellular_component
GO:0000155	44	25	1.51E-11	2.02E-09	4	phosphorelay sensor kinase activity	molecular_function
GO:0023014	44	25	1.51E-11	2.02E-09	5	signal transduction by phosphorylation	biological_process
GO:0016775	44	25	1.51E-11	2.02E-09	5	phosphotransferase activity, nitrogenous group as acceptor	molecular_function
GO:0004673	44	25	1.51E-11	2.02E-09	6	protein histidine kinase activity	molecular_function
GO:0019219	1422	278	1.60E-11	2.08E-09	5	regulation of nucleobase-containing compound metabolic process	biological_process
GO:0009536	1965	362	3.48E-11	4.40E-09	5	plastid	cellular_component
GO:0009637	73	33	3.60E-11	4.44E-09	6	response to blue light	biological_process
GO:0009522	37	22	7.37E-11	8.86E-09	4	photosystem I	cellular_component
GO:0009523	64	30	9.09E-11	1.07E-08	4	photosystem II	cellular_component
GO:0051171	1451	279	9.37E-11	1.07E-08	4	regulation of nitrogen compound metabolic process	biological_process
GO:0003774	148	50	1.91E-10	2.14E-08	8	motor activity	molecular_function
GO:2000112	1409	270	2.79E-10	3.04E-08	6	regulation of cellular macromolecule biosynthetic process	biological_process
GO:0010556	1413	270	3.75E-10	3.99E-08	5	regulation of macromolecule biosynthetic process	biological_process
GO:0031326	1470	279	3.83E-10	3.99E-08	5	regulation of cellular biosynthetic process	biological_process
GO:0043565	507	118	8.00E-10	8.15E-08	6	sequence-specific DNA binding	molecular_function
GO:0003777	97	37	9.35E-10	9.32E-08	9	microtubule motor activity	molecular_function
GO:0009638	23	16	1.03E-09	9.91E-08	5	phototropism	biological_process
GO:0009889	1503	282	1.04E-09	9.91E-08	4	regulation of biosynthetic process	biological_process
GO:0031323	1771	323	1.47E-09	1.38E-07	4	regulation of cellular metabolic process	biological_process
GO:0009628	1566	291	1.53E-09	1.40E-07	3	response to abiotic stimulus	biological_process

Top 50 dif GO enrichment-wat24:con-up

GO ID	All num	DEGs num	P-value	FDR	Level	Term	Type
GO:0005198	606	289	9.46E-117	1.48E-113	2	structural molecule activity	molecular_function
GO:0005576	1222	282	1.61E-34	5.39E-32	2	extracellular region	cellular_component
GO:0032991	2491	466	1.10E-32	3.44E-30	2	macromolecular complex	cellular_component
GO:0016209	188	64	6.01E-17	1.13E-14	2	antioxidant activity	molecular_function
GO:0030054	727	136	7.87E-10	8.99E-08	2	cell junction	cellular_component
GO:0055044	694	130	1.72E-09	1.83E-07	2	symplast	cellular_component
GO:0044421	174	45	4.80E-08	3.51E-06	2	extracellular region part	cellular_component
GO:0045735	76	25	3.69E-07	2.28E-05	2	nutrient reservoir activity	molecular_function
GO:0009055	462	79	7.66E-05	0.0028453	2	electron carrier activity	molecular_function
GO:0071840	2261	303	0.00028231	0.008869	2	cellular component organization or biogenesis	biological_process
GO:0003735	471	277	1.25E-142	2.94E-139	3	structural constituent of ribosome	molecular_function
GO:0030529	801	312	3.29E-97	3.08E-94	3	ribonucleoprotein complex	cellular_component
GO:0043228	1628	418	5.35E-66	2.78E-63	3	non-membrane-bounded organelle	cellular_component
GO:0030312	671	157	5.22E-20	1.36E-17	3	external encapsulating structure	cellular_component
GO:0016491	1856	326	5.15E-18	1.15E-15	3	oxidoreductase activity	molecular_function

GO:0004601	164	59	5.21E-17	1.02E-14	3	peroxidase activity	molecular_function
GO:0005615	141	43	4.13E-10	4.96E-08	3	extracellular space	cellular_component
GO:0005911	702	133	5.20E-10	6.08E-08	3	cell-cell junction	cellular_component
GO:0004857	161	46	1.12E-09	1.25E-07	3	enzyme inhibitor activity	molecular_function
GO:0009506	694	130	1.72E-09	1.83E-07	3	plasmodesma	cellular_component
GO:0071944	3429	486	1.84E-09	1.91E-07	3	cell periphery	cellular_component
GO:0071554	437	89	1.24E-08	1.19E-06	3	cell wall organization or biogenesis	biological_process
GO:0032993	63	23	1.25E-07	8.62E-06	3	protein-DNA complex	cellular_component
GO:0061134	58	20	2.32E-06	0.0001196	3	peptidase regulator activity	molecular_function
GO:0009056	1868	270	3.03E-06	0.0001492	3	catabolic process	biological_process
GO:0044765	1550	224	2.28E-05	0.0009887	3	single-organism transport	biological_process
GO:0048046	346	64	3.41E-05	0.0014011	3	apoplast	cellular_component
GO:0031970	30	11	0.00023854	0.0078083	3	organelle envelope lumen	cellular_component
GO:0097367	40	13	0.00026729	0.0085114	3	carbohydrate derivative binding	molecular_function
GO:0009058	5727	710	0.00034892	0.0106058	3	biosynthetic process	biological_process
GO:0042221	2287	303	0.00060528	0.0175983	3	response to chemical stimulus	biological_process
GO:0071814	3	3	0.00139327	0.0329388	3	protein-lipid complex binding	molecular_function

Top 50 dif GO enrichment-wat24:con-up

GO ID	All num	DEGs num	P-value	FDR	Level	Term	Type
GO:0005840	538	297	1.12E-142	2.94E-139	4	ribosome	cellular_component
GO:0003735	471	277	1.25E-142	2.94E-139	3	structural constituent of ribosome	molecular_function
GO:0005198	606	289	9.46E-117	1.48E-113	2	structural molecule activity	molecular_function
GO:0006412	671	288	6.72E-102	7.86E-99	6	translation	biological_process
GO:0030529	801	312	3.29E-97	3.08E-94	3	ribonucleoprotein complex	cellular_component
GO:0044391	216	146	5.55E-87	4.33E-84	4	ribosomal subunit	cellular_component
GO:0022626	198	132	1.71E-77	1.15E-74	5	cytosolic ribosome	cellular_component
GO:0043228	1628	418	5.35E-66	2.78E-63	3	non-membrane-bounded organelle	cellular_component
GO:0043232	1628	418	5.35E-66	2.78E-63	4	intracellular non-membrane-bounded organelle	cellular_component
GO:0044445	243	136	8.93E-66	4.18E-63	5	cytosolic part	cellular_component
GO:0022625	96	74	2.89E-51	1.23E-48	6	cytosolic large ribosomal subunit	cellular_component
GO:0015934	125	84	4.84E-50	1.89E-47	5	large ribosomal subunit	cellular_component
GO:0015935	91	62	8.52E-38	3.07E-35	5	small ribosomal subunit	cellular_component
GO:0005576	1222	282	1.61E-34	5.39E-32	2	extracellular region	cellular_component
GO:0032991	2491	466	1.10E-32	3.44E-30	2	macromolecular complex	cellular_component
GO:0022627	62	46	4.93E-31	1.44E-28	6	cytosolic small ribosomal subunit	cellular_component
GO:0005618	654	156	9.41E-21	2.59E-18	4	cell wall	cellular_component
GO:0030312	671	157	5.22E-20	1.36E-17	3	external encapsulating structure	cellular_component
GO:0044267	2316	392	7.68E-19	1.89E-16	5	cellular protein metabolic process	biological_process
GO:0042744	114	49	4.60E-18	1.08E-15	5	hydrogen peroxide catabolic process	biological_process
GO:0016491	1856	326	5.15E-18	1.15E-15	3	oxidoreductase activity	molecular_function
GO:0070301	121	50	1.57E-17	3.35E-15	6	cellular response to hydrogen peroxide	biological_process
GO:0004601	164	59	5.21E-17	1.02E-14	3	peroxidase activity	molecular_function
GO:0016684	164	59	5.21E-17	1.02E-14	4	oxidoreductase activity, acting on peroxide as acceptor	molecular_function
GO:0016209	188	64	6.01E-17	1.13E-14	2	antioxidant activity	molecular_function
GO:0042743	131	51	1.51E-16	2.72E-14	5	hydrogen peroxide metabolic process	biological_process
GO:0020037	452	112	1.78E-16	3.08E-14	5	heme binding	molecular_function
GO:0046906	485	115	2.13E-15	3.56E-13	4	tetrapyrrole binding	molecular_function
GO:0034614	141	51	5.24E-15	8.46E-13	6	cellular response to reactive oxygen species	biological_process
GO:0034599	148	51	4.92E-14	7.68E-12	5	cellular response to oxidative stress	biological_process

GO:0006414	50	27	1.42E-13	2.14E-11	6	translational elongation	biological_process
GO:0072593	173	55	2.35E-13	3.44E-11	4	reactive oxygen species metabolic process	biological_process
GO:0042542	164	52	1.18E-12	1.68E-10	5	response to hydrogen peroxide	biological_process
GO:0003746	66	30	2.24E-12	3.09E-10	7	translation elongation factor activity	molecular_function
GO:0005730	378	89	4.89E-12	6.54E-10	5	nucleolus	cellular_component
GO:0000302	241	63	6.67E-11	8.68E-09	5	response to reactive oxygen species	biological_process
GO:0000272	192	54	7.95E-11	1.01E-08	6	polysaccharide catabolic process	biological_process
GO:0010467	3537	506	1.79E-10	2.21E-08	5	gene expression	biological_process
GO:0005615	141	43	4.13E-10	4.96E-08	3	extracellular space	cellular_component
GO:0005911	702	133	5.20E-10	6.08E-08	3	cell-cell junction	cellular_component
GO:0030054	727	136	7.87E-10	8.99E-08	2	cell junction	cellular_component
GO:0004857	161	46	1.12E-09	1.25E-07	3	enzyme inhibitor activity	molecular_function
GO:0055044	694	130	1.72E-09	1.83E-07	2	symplast	cellular_component
GO:0009506	694	130	1.72E-09	1.83E-07	3	plasmodesma	cellular_component
GO:0071944	3429	486	1.84E-09	1.91E-07	3	cell periphery	cellular_component
GO:0005829	1354	221	2.31E-09	2.35E-07	5	cytosol	cellular_component
GO:0006979	368	80	3.24E-09	3.23E-07	4	response to oxidative stress	biological_process
GO:0000786	51	22	6.43E-09	6.27E-07	4	nucleosome	cellular_component
GO:0071554	437	89	1.24E-08	1.19E-06	3	cell wall organization or biogenesis	biological_process
GO:0042274	20	13	1.49E-08	1.30E-06	5	ribosomal small subunit biogenesis	biological_process

Top 50 dif GO enrichment-wat24:con-down

GO ID	All num	DEGs num	P-value	FDR	Level	Term	Type
GO:0001071	1345	221	9.30E-17	9.96E-14	2	nucleic acid binding transcription factor activity	molecular_function
GO:0005488	12800	1327	4.26E-08	4.30E-06	2	binding	molecular_function
GO:0050896	5231	576	1.25E-05	0.000673	2	response to stimulus	biological_process
GO:0023052	1715	213	1.94E-05	0.000978	2	signaling	biological_process
GO:0008152	11841	1199	0.000236	0.007404	2	metabolic process	biological_process
GO:0044423	12	6	0.000407	0.010967	2	virion part	cellular_component
GO:0019012	13	6	0.000694	0.016943	2	virion	cellular_component
GO:0003700	1345	221	9.30E-17	9.96E-14	3	sequence-specific DNA binding transcription factor activity	molecular_function
GO:0006807	6258	736	5.28E-13	1.56E-10	3	nitrogen compound metabolic process	biological_process
GO:1901363	9640	1062	3.60E-12	8.61E-10	3	heterocyclic compound binding	molecular_function
GO:0097159	9651	1062	5.08E-12	1.14E-09	3	organic cyclic compound binding	molecular_function
GO:0009719	1364	203	3.21E-11	5.60E-09	3	response to endogenous stimulus	biological_process
GO:0042221	2287	288	1.55E-07	1.45E-05	3	response to chemical stimulus	biological_process
GO:0009607	858	118	2.40E-05	0.001163	3	response to biotic stimulus	biological_process
GO:0044700	1710	210	4.48E-05	0.002069	3	single organism signaling	biological_process
GO:0071704	10911	1120	4.69E-05	0.002118	3	organic substance metabolic process	biological_process
GO:0016787	4278	472	9.00E-05	0.003519	3	hydrolase activity	molecular_function
GO:0051707	790	107	0.000105	0.003911	3	response to other organism	biological_process
GO:0009628	1566	191	0.000147	0.005114	3	response to abiotic stimulus	biological_process
GO:0044237	10081	1034	0.000182	0.006114	3	cellular metabolic process	biological_process
GO:0009058	5727	611	0.000254	0.00779	3	biosynthetic process	biological_process
GO:0009791	1125	141	0.000349	0.010298	3	post-embryonic development	biological_process
GO:0042754	5	4	0.000375	0.010725	3	negative regulation of circadian rhythm	biological_process
GO:0003682	340	52	0.000401	0.010967	3	chromatin binding	molecular_function
GO:0019028	12	6	0.000407	0.010967	3	viral capsid	cellular_component
GO:0019222	2140	246	0.000665	0.016759	3	regulation of metabolic process	biological_process
GO:0044238	10226	1039	0.00081	0.019149	3	primary metabolic process	biological_process

GO:0043167	9044	924	0.001148	0.023403	3	ion binding	molecular_function

Top 50 dif GO enrichment wat24:con-down

GO_ID	All_num	DEGs_num	Pvalue	FDR	Level	Term	Type
GO:0015074	1125	201	2.64E-19	1.01E-15	6	DNA integration	biological_process
GO:0001071	1345	221	9.30E-17	9.96E-14	2	nucleic acid binding transcription factor activity	molecular_function
GO:0003700	1345	221	9.30E-17	9.96E-14	3	sequence-specific DNA binding transcription factor activity	molecular_function
GO:0090304	4585	585	1.04E-16	9.96E-14	5	nucleic acid metabolic process	biological_process
GO:1901360	5919	722	1.96E-16	1.50E-13	4	organic cyclic compound metabolic process	biological_process
GO:0046483	5667	693	7.31E-16	4.67E-13	4	heterocycle metabolic process	biological_process
GO:0006139	5376	660	2.56E-15	1.40E-12	4	nucleobase-containing compound metabolic process	biological_process
GO:0006725	5817	704	4.25E-15	2.03E-12	4	cellular aromatic compound metabolic process	biological_process
GO:0034641	5701	689	1.47E-14	6.26E-12	4	cellular nitrogen compound metabolic process	biological_process
GO:0006351	2276	322	2.07E-14	7.93E-12	6	transcription, DNA-dependent	biological_process
GO:0032774	2282	322	2.96E-14	1.03E-11	6	RNA biosynthetic process	biological_process
GO:0003676	4747	588	4.02E-14	1.28E-11	4	nucleic acid binding	molecular_function
GO:0006807	6258	736	5.28E-13	1.56E-10	3	nitrogen compound metabolic process	biological_process
GO:0018130	2786	369	2.52E-12	6.88E-10	5	heterocycle biosynthetic process	biological_process
GO:1901362	2982	390	3.00E-12	7.66E-10	5	organic cyclic compound biosynthetic process	biological_process
GO:1901363	9640	1062	3.60E-12	8.61E-10	3	heterocyclic compound binding	molecular_function
GO:0097159	9651	1062	5.08E-12	1.14E-09	3	organic cyclic compound binding	molecular_function
GO:0019438	2870	375	1.02E-11	2.18E-09	5	aromatic compound biosynthetic process	biological_process
GO:0070001	655	116	2.60E-11	4.98E-09	6	aspartic-type peptidase activity	molecular_function
GO:0004190	655	116	2.60E-11	4.98E-09	7	aspartic-type endopeptidase activity	molecular_function
GO:0034654	2580	341	2.80E-11	5.12E-09	5	nucleobase-containing compound biosynthetic process	biological_process
GO:0009719	1364	203	3.21E-11	5.60E-09	3	response to endogenous stimulus	biological_process
GO:0044271	2774	360	7.20E-11	1.20E-08	5	cellular nitrogen compound biosynthetic process	biological_process
GO:0003964	752	127	7.77E-11	1.24E-08	7	RNA-directed DNA polymerase activity	molecular_function
GO:0003677	2302	307	1.26E-10	1.93E-08	5	DNA binding	molecular_function
GO:0010033	1584	226	1.33E-10	1.96E-08	4	response to organic substance	biological_process
GO:0009725	1235	183	4.70E-10	6.58E-08	4	response to hormone stimulus	biological_process
GO:0034061	789	129	4.81E-10	6.58E-08	6	DNA polymerase activity	molecular_function
GO:0004175	906	142	1.20E-09	1.59E-07	6	endopeptidase activity	molecular_function
GO:0000155	44	19	4.74E-09	5.51E-07	4	phosphorelay sensor kinase activity	molecular_function
GO:0023014	44	19	4.74E-09	5.51E-07	5	signal transduction by phosphorylation	biological_process
GO:0016775	44	19	4.74E-09	5.51E-07	5	phosphotransferase activity, nitrogenous group as acceptor	molecular_function
GO:0004673	44	19	4.74E-09	5.51E-07	6	protein histidine kinase activity	molecular_function
GO:0016779	931	141	1.32E-08	1.48E-06	5	nucleotidyltransferase activity	molecular_function
GO:0006259	1753	234	2.64E-08	2.89E-06	5	DNA metabolic process	biological_process
GO:0005034	11	9	2.85E-08	3.03E-06	5	osmosensor activity	molecular_function
GO:0004519	516	88	4.15E-08	4.30E-06	6	endonuclease activity	molecular_function
GO:0005488	12800	1327	4.26E-08	4.30E-06	2	binding	molecular_function
GO:1901700	1372	190	4.47E-08	4.39E-06	4	response to oxygen-containing compound	biological_process
GO:0046914	3057	370	1.49E-07	1.43E-05	6	transition metal ion binding	molecular_function
GO:0042221	2287	288	1.55E-07	1.45E-05	3	response to chemical stimulus	biological_process
GO:0009751	178	40	1.95E-07	1.78E-05	5	response to salicylic acid stimulus	biological_process
GO:0008270	2314	290	2.17E-07	1.93E-05	7	zinc ion binding	molecular_function
GO:0008233	1200	167	2.25E-07	1.96E-05	4	peptidase activity	molecular_function
GO:0070011	1160	162	2.75E-07	2.34E-05	5	peptidase activity, acting on L-amino acid peptides	molecular_function
GO:0009733	367	66	2.88E-07	2.40E-05	5	response to auxin stimulus	biological_process

GO:0009755	768	116	3.04E-07	2.48E-05	5	hormone-mediated signaling pathway	biological_process
GO:0004518	604	95	5.98E-07	4.72E-05	5	nuclease activity	molecular_function
GO:0000160	234	47	6.03E-07	4.72E-05	5	phosphorelay signal transduction system	biological_process
GO:0009723	307	57	6.32E-07	4.84E-05	5	response to ethylene stimulus	biological_process

Top 50 dif GO enrichment wat 24:wat6 up

GO ID	All num	DEGs num	P-value	FDR	Level	Term	Type
GO:0005198	606	168	5.63E-40	7.43E-37	2	structural molecule activity	molecular_function
GO:0005576	1222	187	3.82E-12	1.16E-09	2	extracellular region	cellular_component
GO:0016209	188	43	2.35E-08	3.57E-06	2	antioxidant activity	molecular_function
GO:0032991	2491	306	1.06E-07	1.35E-05	2	macromolecular complex	cellular_component
GO:0001071	1345	177	1.16E-06	0.000107	2	nucleic acid binding transcription factor activity	molecular_function
GO:0045735	76	18	0.00017	0.008084	2	nutrient reservoir activity	molecular_function
GO:0009055	462	62	0.002242	0.06521	2	electron carrier activity	molecular_function
GO:0044699	7199	722	0.006214	0.130001	2	single-organism process	biological_process
GO:0003735	471	153	9.86E-46	1.95E-42	3	structural constituent of ribosome	molecular_function
GO:0030529	801	177	1.59E-28	1.26E-25	3	ribonucleoprotein complex	cellular_component
GO:0043228	1628	273	3.79E-23	1.67E-20	3	non-membrane-bounded organelle	cellular_component
GO:0004601	164	41	3.21E-09	6.34E-07	3	peroxidase activity	molecular_function
GO:0006950	3162	374	1.97E-07	2.29E-05	3	response to stress	biological_process
GO:0003700	1345	177	1.16E-06	0.000107	3	sequence-specific DNA binding transcription factor activity	molecular_function
GO:0009056	1868	232	2.10E-06	0.000181	3	catabolic process	biological_process
GO:0042754	5	5	7.01E-06	0.000523	3	negative regulation of circadian rhythm	biological_process
GO:0030312	671	96	1.45E-05	0.000986	3	external encapsulating structure	cellular_component
GO:0048046	346	57	1.61E-05	0.001044	3	apoplast	cellular_component
GO:0009628	1566	194	1.81E-05	0.001154	3	response to abiotic stimulus	biological_process
GO:0071944	3429	379	0.000107	0.005373	3	cell periphery	cellular_component
GO:0016491	1856	216	0.000279	0.012272	3	oxidoreductase activity	molecular_function
GO:0045848	3	3	0.000809	0.027366	3	positive regulation of nitrogen utilization	biological_process
GO:0003682	340	48	0.002456	0.069892	3	chromatin binding	molecular_function
GO:0042221	2287	251	0.002672	0.073537	3	response to chemical stimulus	biological_process
GO:0051656	63	13	0.004806	0.104722	3	establishment of organelle localization	biological_process

Top 50 dif GO enrichment wat 24:wat6-up

GO ID	All num	DEGs num	P-value	FDR	Level	Term	Type
GO:0005840	538	170	6.32E-49	2.50E-45	4	ribosome	cellular_component
GO:0003735	471	153	9.86E-46	1.95E-42	3	structural constituent of ribosome	molecular_function
GO:0005198	606	168	5.63E-40	7.43E-37	2	structural molecule activity	molecular_function
GO:0006412	671	166	7.43E-33	7.35E-30	6	translation	biological_process
GO:0030529	801	177	1.59E-28	1.26E-25	3	ribonucleoprotein complex	cellular_component
GO:0044391	216	77	1.98E-26	1.31E-23	4	ribosomal subunit	cellular_component
GO:0022626	198	69	3.67E-23	1.67E-20	5	cytosolic ribosome	cellular_component
GO:0043228	1628	273	3.79E-23	1.67E-20	3	non-membrane-bounded organelle	cellular_component
GO:0043232	1628	273	3.79E-23	1.67E-20	4	intracellular non-membrane-bounded organelle	cellular_component
GO:0044445	243	70	3.04E-18	1.20E-15	5	cytosolic part	cellular_component
GO:0015934	125	42	5.13E-14	1.84E-11	5	large ribosomal subunit	cellular_component
GO:0015935	91	35	6.47E-14	2.13E-11	5	small ribosomal subunit	cellular_component
GO:0005576	1222	187	3.82E-12	1.16E-09	2	extracellular region	cellular_component
GO:0022627	62	26	1.04E-11	2.93E-09	6	cytosolic small ribosomal subunit	cellular_component
GO:0022625	96	33	1.26E-11	3.33E-09	6	cytosolic large ribosomal subunit	cellular_component

GO:0042542	164	42	9.40E-10	2.32E-07	5	response to hydrogen peroxide	biological_process
GO:0042744	114	33	2.08E-09	4.83E-07	5	hydrogen peroxide catabolic process	biological_process
GO:0070301	121	34	2.80E-09	6.15E-07	6	cellular response to hydrogen peroxide	biological_process
GO:0004601	164	41	3.21E-09	6.34E-07	3	peroxidase activity	molecular_function
GO:0016684	164	41	3.21E-09	6.34E-07	4	oxidoreductase activity, acting on peroxide as acceptor	molecular_function
GO:0046906	485	86	3.93E-09	7.40E-07	4	tetrapyrrole binding	molecular_function
GO:0042743	131	35	7.03E-09	1.26E-06	5	hydrogen peroxide metabolic process	biological_process
GO:0044267	2316	294	8.38E-09	1.44E-06	5	cellular protein metabolic process	biological_process
GO:0020037	452	80	1.49E-08	2.46E-06	5	heme binding	molecular_function
GO:0034614	141	36	1.59E-08	2.51E-06	6	cellular response to reactive oxygen species	biological_process
GO:0016209	188	43	2.35E-08	3.57E-06	2	antioxidant activity	molecular_function
GO:0051301	391	71	3.33E-08	4.89E-06	4	cell division	biological_process
GO:0004553	478	82	4.14E-08	5.85E-06	5	hydrolase activity, hydrolyzing O-glycosyl compounds	molecular_function
GO:0034599	148	36	6.11E-08	8.33E-06	5	cellular response to oxidative stress	biological_process
GO:0010035	854	127	7.09E-08	9.35E-06	4	response to inorganic substance	biological_process
GO:0032991	2491	306	1.06E-07	1.35E-05	2	macromolecular complex	cellular_component
GO:0000302	241	49	1.36E-07	1.64E-05	5	response to reactive oxygen species	biological_process
GO:0016798	523	86	1.37E-07	1.64E-05	4	hydrolase activity, acting on glycosyl bonds	molecular_function
GO:0006950	3162	374	1.97E-07	2.29E-05	3	response to stress	biological_process
GO:0003746	66	21	2.97E-07	3.36E-05	7	translation elongation factor activity	molecular_function
GO:0072593	173	38	4.51E-07	4.96E-05	4	reactive oxygen species metabolic process	biological_process
GO:0005874	238	47	5.91E-07	6.32E-05	4	microtubule	cellular_component
GO:0009644	43	16	7.06E-07	7.35E-05	7	response to high light intensity	biological_process
GO:0006979	368	64	7.63E-07	7.74E-05	4	response to oxidative stress	biological_process
GO:0007018	130	31	7.85E-07	7.76E-05	5	microtubule-based movement	biological_process
GO:0000272	192	40	9.73E-07	9.39E-05	6	polysaccharide catabolic process	biological_process
GO:0001071	1345	177	1.16E-06	0.000107	2	nucleic acid binding transcription factor activity	molecular_function
GO:0003700	1345	177	1.16E-06	0.000107	3	sequence-specific DNA binding transcription factor activity	molecular_function
GO:0006414	50	17	1.40E-06	0.000126	6	translational elongation	biological_process
GO:0016998	67	20	1.76E-06	0.000155	5	cell wall macromolecule catabolic process	biological_process
GO:0009056	1868	232	2.10E-06	0.000181	3	catabolic process	biological_process
GO:0009415	296	53	2.74E-06	0.00023	4	response to water stimulus	biological_process
GO:0042274	20	10	3.73E-06	0.000308	5	ribosomal small subunit biogenesis	biological_process
GO:0009414	287	51	5.23E-06	0.000423	4	response to water deprivation	biological_process
GO:0002181	17	9	6.35E-06	0.000502	7	cytoplasmic translation	biological_process

Top 50 dif GO enrichment wat 24:wat6-down

GO ID	All num	DEGs num	P-value	FDR	Level	Term	Type
GO:0050896	5231	312	3.04E-12	1.72E-09	2	response to stimulus	biological_process
GO:0005215	1416	105	1.15E-08	1.71E-06	2	transporter activity	molecular_function
GO:0005576	1222	85	4.27E-06	0.000201	2	extracellular region	cellular_component
GO:0009055	462	40	1.62E-05	0.000578	2	electron carrier activity	molecular_function
GO:0048511	143	17	0.000119	0.002887	2	rhythmic process	biological_process
GO:0045735	76	11	0.00034	0.006724	2	nutrient reservoir activity	molecular_function
GO:0023052	1715	100	0.000635	0.009452	2	signaling	biological_process
GO:0042221	2287	182	1.10E-17	3.10E-14	3	response to chemical stimulus	biological_process
GO:0016491	1856	156	3.23E-17	4.56E-14	3	oxidoreductase activity	molecular_function

GO:0009719	1364	117	1.41E-13	1.32E-10	3	response to endogenous stimulus	biological_process
GO:0044710	3304	211	1.53E-10	3.61E-08	3	single-organism metabolic process	biological_process
GO:0048037	658	65	1.99E-10	4.32E-08	3	cofactor binding	molecular_function
GO:0022892	1051	82	6.32E-08	8.50E-06	3	substrate-specific transporter activity	molecular_function
GO:0051240	97	18	1.15E-07	1.31E-05	3	positive regulation of multicellular organismal process	biological_process
GO:0022857	1145	86	1.58E-07	1.65E-05	3	transmembrane transporter activity	molecular_function
GO:0048046	346	37	2.43E-07	2.37E-05	3	apoplast	cellular_component
GO:0051094	92	16	1.44E-06	0.000101	3	positive regulation of developmental process	biological_process
GO:0090484	128	18	7.59E-06	0.000335	3	drug transporter activity	molecular_function
GO:0042752	52	11	9.05E-06	0.000387	3	regulation of circadian rhythm	biological_process
GO:0009628	1566	100	2.23E-05	0.000725	3	response to abiotic stimulus	biological_process
GO:0031224	4742	250	4.82E-05	0.001375	3	intrinsic to membrane	cellular_component
GO:0044765	1550	96	0.000103	0.002562	3	single-organism transport	biological_process
GO:0070469	113	14	0.0003	0.006372	3	respiratory chain	cellular_component
GO:0051050	32	7	0.000317	0.006626	3	positive regulation of transport	biological_process
GO:0042445	114	14	0.000329	0.006724	3	hormone metabolic process	biological_process
GO:0007623	116	14	0.000394	0.007521	3	circadian rhythm	biological_process
GO:0006950	3162	170	0.000458	0.00856	3	response to stress	biological_process

Top 50 dif GOs enrichment wat 24:wat6 down

GO ID	All num	DEGs num	P-value	FDR	Level	Term	Type
GO:0042221	2287	182	1.10E-17	3.10E-14	3	response to chemical stimulus	biological_process
GO:0016491	1856	156	3.23E-17	4.56E-14	3	oxidoreductase activity	molecular_function
GO:0009719	1364	117	1.41E-13	1.32E-10	3	response to endogenous stimulus	biological_process
GO:0009733	367	50	2.78E-13	1.97E-10	5	response to auxin stimulus	biological_process
GO:0050896	5231	312	3.04E-12	1.72E-09	2	response to stimulus	biological_process
GO:0009725	1235	105	5.05E-12	2.38E-09	4	response to hormone stimulus	biological_process
GO:0010033	1584	125	6.44E-12	2.60E-09	4	response to organic substance	biological_process
GO:0080167	105	24	9.33E-12	3.30E-09	4	response to karrikin	biological_process
GO:0010378	9	8	8.68E-11	2.57E-08	4	temperature compensation of the circadian clock	biological_process
GO:0071365	247	36	9.11E-11	2.57E-08	6	cellular response to auxin stimulus	biological_process
GO:0009734	238	35	1.29E-10	3.32E-08	6	auxin mediated signaling pathway	biological_process
GO:0044710	3304	211	1.53E-10	3.61E-08	3	single-organism metabolic process	biological_process
GO:0048037	658	65	1.99E-10	4.32E-08	3	cofactor binding	molecular_function
GO:0015291	349	43	3.49E-10	7.03E-08	5	secondary active transmembrane transporter activity	molecular_function
GO:0005506	489	53	3.77E-10	7.10E-08	7	iron ion binding	molecular_function
GO:0016638	48	15	6.62E-10	1.17E-07	4	oxidoreductase activity, acting on the CH-NH2 group of donors	molecular_function
GO:0048578	13	8	1.07E-08	1.67E-06	4	positive regulation of long-day photoperiodism, flowering	biological_process
GO:0016639	13	8	1.07E-08	1.67E-06	5	oxidoreductase activity, acting on the CH-NH2 group of donors, NAD or NADP as acceptor	molecular_function
GO:0005215	1416	105	1.15E-08	1.71E-06	2	transporter activity	molecular_function
GO:0009755	768	66	3.91E-08	5.52E-06	5	hormone-mediated signaling pathway	biological_process
GO:0022892	1051	82	6.32E-08	8.50E-06	3	substrate-specific transporter activity	molecular_function
GO:0022891	932	75	6.94E-08	8.91E-06	4	substrate-specific transmembrane transporter activity	molecular_function
GO:0020037	452	45	1.03E-07	1.27E-05	5	heme binding	molecular_function

GO:0051240	97	18	1.15E-07	1.31E-05	3	positive regulation of multicellular organismal process	biological_process
GO:0071495	842	69	1.20E-07	1.31E-05	4	cellular response to endogenous stimulus	biological_process
GO:0046906	485	47	1.21E-07	1.31E-05	4	tetrapyrrole binding	molecular_function
GO:0022857	1145	86	1.58E-07	1.65E-05	3	transmembrane transporter activity	molecular_function
GO:0032870	816	67	1.71E-07	1.73E-05	5	cellular response to hormone stimulus	biological_process
GO:0048046	346	37	2.43E-07	2.37E-05	3	apoplast	cellular_component
GO:0016705	487	46	3.45E-07	3.25E-05	4	oxidoreductase activity, acting on paired donors, with incorporation or reduction of molecular oxygen	molecular_function
GO:0050662	473	45	3.78E-07	3.45E-05	4	coenzyme binding	molecular_function
GO:0022904	56	13	4.49E-07	3.96E-05	6	respiratory electron transport chain	biological_process
GO:0043436	1141	84	5.12E-07	4.39E-05	5	oxoacid metabolic process	biological_process
GO:0006082	1147	84	6.35E-07	5.28E-05	4	organic acid metabolic process	biological_process
GO:0070887	1169	85	7.29E-07	5.79E-05	4	cellular response to chemical stimulus	biological_process
GO:1901700	1372	96	7.52E-07	5.79E-05	4	response to oxygen-containing compound	biological_process
GO:0045181	6	5	7.79E-07	5.79E-05	6	glutamate synthase activity, NAD(P)H as acceptor	molecular_function
GO:0016040	6	5	7.79E-07	5.79E-05	7	glutamate synthase (NADH) activity	molecular_function
GO:0050660	161	22	1.25E-06	9.09E-05	5	flavin adenine dinucleotide binding	molecular_function
GO:0051094	92	16	1.44E-06	0.000101	3	positive regulation of developmental process	biological_process
GO:0043449	36	10	1.64E-06	0.000103	4	cellular alkene metabolic process	biological_process
GO:0009692	36	10	1.64E-06	0.000103	5	ethylene metabolic process	biological_process
GO:0043450	36	10	1.64E-06	0.000103	5	alkene biosynthetic process	biological_process
GO:1900674	36	10	1.64E-06	0.000103	5	olefin biosynthetic process	biological_process
GO:0009693	36	10	1.64E-06	0.000103	6	ethylene biosynthetic process	biological_process
GO:0010035	854	66	1.79E-06	0.00011	4	response to inorganic substance	biological_process
GO:0048586	22	8	1.88E-06	0.000111	5	regulation of long-day photoperiodism, flowering	biological_process
GO:0048582	63	13	1.89E-06	0.000111	4	positive regulation of post-embryonic development	biological_process
GO:0002705	7	5	2.63E-06	0.000138	5	positive regulation of leukocyte mediated immunity	biological_process
GO:0002821	7	5	2.63E-06	0.000138	5	positive regulation of adaptive immune response	biological_process
