# Supplementary material for: De Novo Characterization of the Mung Bean Transcriptome and Transcriptomic Analysis of Adventitious Rooting in Seedlings Using RNA-Seq
Source: PLoS One. 2015 Jul 15;10(7):e0132969. doi: 10.1371/journal.pone.0132969 (PMC4503682; doi:10.1371/journal.pone.0132969)
Supplement: S5 Table — (PDF) [file pone.0132969.s009.pdf]

S5 Table. Top list of significant KOs

wat6:con-down

KO ID	All num	DEGs num	P-value	FDR	Description
ko00195	53	20	1.51E-06	0.0004	Photosynthesis
ko00710	46	15	0.0002	0.0149	Carbon fixation in photosynthetic organisms
ko00906	20	9	0.0003	0.0149	Carotenoid biosynthesis
ko00910	31	10	0.0027	0.1250	Nitrogen metabolism
ko00600	34	10	0.0057	0.1740	Sphingolipid metabolism
ko00561	52	13	0.0078	0.1867	Glycerolipid metabolism
ko00860	58	14	0.0082	0.1867	Porphyrin and chlorophyll metabolism
ko04712	23	7	0.0162	0.2524	Circadian rhythm - plant
ko00053	35	9	0.0210	0.2942	Ascorbate and aldarate metabolism
ko04744	11	4	0.0355	0.3684	Phototransduction
ko02010	42	9	0.0618	0.5110	ABC transporters
ko04075	130	21	0.1043	0.7124	Plant hormone signal transduction

wat6:con-up

KO ID	All num	DEGs num	P-value	FDR	Description
ko03010	337	110	4.42E-29	1.02E-26	Ribosome
ko00940	69	25	2.66E-08	3.07E-06	Phenylpropanoid biosynthesis
ko00360	57	21	2.55E-07	1.96E-05	Phenylalanine metabolism
ko00980	40	13	0.0002	0.0078	Metabolism of xenobiotics by cytochrome P450
ko00909	7	5	0.0003	0.0078	Sesquiterpenoid and triterpenoid biosynthesis
ko00480	63	17	0.0003	0.0078	Glutathione metabolism
ko04610	18	8	0.0003	0.0078	Complement and coagulation cascades
ko00270	68	17	0.0009	0.0181	Cysteine and methionine metabolism
ko00260	52	14	0.0011	0.0216	Glycine
ko00750	10	5	0.0025	0.0451	Vitamin B6 metabolism
ko00920	29	9	0.0030	0.0496	Sulfur metabolism
ko01230	180	32	0.0040	0.0610	Biosynthesis of amino acids

wat24:con-up

KO_ID	All_num	DEGs_num	Pvalue	FDR	Description
ko03010	337	212	5.34E-115	1.46E-112	Ribosome
ko00940	69	34	1.92E-13	2.63E-11	Phenylpropanoid biosynthesis
ko00360	57	27	2.00E-10	1.82E-08	Phenylalanine metabolism
ko00909	7	5	0.0006	0.0276	Sesquiterpenoid and triterpenoid biosynthesis
ko04110	94	21	0.0077	0.2684	Cell cycle
ko00040	49	13	0.0078	0.2684	Pentose and glucuronate interconversions
ko00945	26	8	0.0140	0.4267	Stilbenoid
ko00941	17	6	0.0162	0.4442	Flavonoid biosynthesis
ko04610	18	6	0.0217	0.5410	Complement and coagulation cascades
ko00980	40	10	0.0280	0.6097	Metabolism of xenobiotics by cytochrome P450

wat24:con-down

KO_ID	All_num	DEGs_num	Pvalue	FDR	Description
ko00073	12	8	1.40E-06	0.0002	Cutin

ko00904	8	6	1.20E-05	0.0011	Diterpenoid biosynthesis
ko04060	58	15	0.0001	0.0081	Cytokine-cytokine receptor interaction
ko04712	23	7	0.0031	0.1194	Circadian rhythm - plant
ko04744	11	4	0.0126	0.3408	Phototransduction
ko04075	130	19	0.0217	0.4567	Plant hormone signal transduction
ko04062	36	7	0.0377	0.5445	Chemokine signaling pathway
ko04020	59	9	0.0776	0.7337	Calcium signaling pathway
ko00053	35	6	0.0879	0.7337	Ascorbate and aldarate metabolism
ko04210	27	5	0.0883	0.7337	Apoptosis
ko00906	20	4	0.0978	0.7337	Carotenoid biosynthesis

wat24:wat6-up

KO_ID	All_num	DEGs_num	Pvalue	FDR	Description
ko03010	337	117	5.20E-43	1.32E-40	Ribosome
ko00940	69	21	3.96E-07	5.03E-05	Phenylpropanoid biosynthesis
ko00360	57	15	0.0001	0.0077	Phenylalanine metabolism
ko04110	94	20	0.0002	0.0122	Cell cycle
ko00052	38	11	0.0004	0.0170	Galactose metabolism
ko00500	119	19	0.0108	0.3036	Starch and sucrose metabolism
ko00710	46	8	0.0541	0.9999	Carbon fixation in photosynthetic organisms
ko00980	40	7	0.0674	0.9999	Metabolism of xenobiotics by cytochrome P450
ko00040	49	8	0.0738	0.9999	Pentose and glucuronate interconversions
ko02010	42	7	0.0835	0.9999	ABC transporters
ko04626	77	11	0.0891	0.9999	Plant-pathogen interaction
ko04141	145	17	0.1693	0.9999	Protein processing in endoplasmic reticulum
ko00195	53	7	0.20610	0.9999	Photosynthesis

wat24:wat6-down

KO ID	All num	DEGs num	P-value	FDR	Description
ko00190	143	16	0.0003	0.0293	Oxidative phosphorylation
ko00910	31	6	0.0017	0.0753	Nitrogen metabolism
ko04075	130	13	0.0033	0.1095	Plant hormone signal transduction
ko00904	8	3	0.0036	0.1095	Diterpenoid biosynthesis
ko00195	53	7	0.0067	0.1720	Photosynthesis
ko00270	68	8	0.0077	0.1743	Cysteine and methionine metabolism
ko04712	23	4	0.0149	0.3003	Circadian rhythm - plant
ko00940	69	7	0.0263	0.4102	Phenylpropanoid biosynthesis
ko00920	29	4	0.0329	0.4257	Sulfur metabolism
ko00360	57	6	0.0329	0.4257	Phenylalanine metabolism
ko00908	11	2	0.0770	0.5809	Zeatin biosynthesis
ko00380	26	3	0.0964	0.6036	Tryptophan metabolism
ko00480	63	5	0.1288	0.6277	Glutathione metabolism
