# Supplementary material for: De Novo Characterization of the Mung Bean Transcriptome and Transcriptomic Analysis of Adventitious Rooting in Seedlings Using RNA-Seq
Source: PLoS One. 2015 Jul 15;10(7):e0132969. doi: 10.1371/journal.pone.0132969 (PMC4503682; doi:10.1371/journal.pone.0132969)
Supplement: S6 Table — (PDF) [file pone.0132969.s010.pdf]

S6 Table. Top list of significant DEGs in the three samples

Top 50 DGEs con:wat6-up

Gene_ID	Reads1	RPKM1	Reads2	RPKM2	Log2	Pvalue	FDR	NR ref	Description
Vr39448_c0_seq1	0	0.00	973	53.6	NA	0	0	XP_004500339.1	cationic peroxidase 1-like [Cicer arietinum]
Vr44006_c0_seq1	0	0.00	489	9.79	NA	0	0	BAJ22384.1	terminal flower 1a [Vigna unguiculata]
Vr58732_c0_seq1	0	0.00	370	6.08	NA	0	0	XP_003551178.1	cationic peroxidase 1-like [Glycine max]
Vr69119_c0_seq1	0	0.00	335	5.16	NA	0	0	XP_003520006.1	probable pectinesterase 29-like [Glycine max]
Vr43029_c0_seq1	0	0.00	197	6.19	NA	0	0	XP_003529031.1	patatin group A-3-like [Glycine max]
Vr37067_c0_seq4	0	0.00	128	1.2	NA	0	0	XP_003548678.1	G-type lectin S-receptor-like serine/threonine-protein kinase
Vr35851_c0_seq1	825	11.60	43774	752	-6.02	0	0	NP_001237786.1	Kunitz trypsin protease inhibitor precursor [Glycine max]
Vr38231_c0_seq1	1125	17.01	29772	550	-5.02	0	0	NP_001235010.1	uncharacterized protein LOC100500484 precursor [Glycine max]
Vr35207_c0_seq2	668	7.68	18340	258	-5.07	1.6E-11	1.1E-10	XP_003537598.1	isoflavone reductase-like [Glycine max]
Vr38993_c0_seq1	509	3.90	15083	141	-5.18	9.5E-12	6.4E-11	NP_001240000.1	isoflavone 2'-hydroxylase-like [Glycine max]
Vr31128_c0_seq2	366	4.20	12075	169	-5.33	4.7E-12	3.3E-11	XP_003547616.1	cationic peroxidase 2-like [Glycine max]
Vr39039_c0_seq1	52	0.82	10890	210	-8.00	6.2E-14	4.9E-13	ADX66343.1	pathogenesis-related protein [Glycine max]
Vr36526_c0_seq1	174.5	6.49	10690	486	-6.23	5.8E-13	4.4E-12	ABS70717.1	pathogen-related protein [Vigna angularis]
Vr40472_c0_seq1	109	1.22	7732	106	-6.44	0	0	CAA61279.1	basic chitinase class 3 [Vigna unguiculata]
Vr22610_c0_seq1	13	0.19	7681	140	-9.50	0	0	XP_003544327.1	cationic peroxidase 1-like [Glycine max]
Vr39180_c0_seq1	2	0.04	6952	155	-12.05	1.6E-15	1.3E-14	XP_003538325.1	cationic peroxidase 1-like [Glycine max]
Vr34333_c0_seq1	170	2.00	6546	94.3	-5.56	6.2E-13	4.7E-12	NP_001236116.1	uncharacterized protein LOC100500048 precursor [Glycine max]
Vr39339_c0_seq1	227	2.28	6519	80.2	-5.13	7.2E-13	5.4E-12	NP_001236520.1	vestitone reductase [Glycine max]
Vr35930_c0_seq1	165	2.51	6102	114	-5.50	7.6E-13	5.7E-12	NP_001236934.1	uncharacterized protein LOC100500621 precursor [Glycine max]
Vr39225_c0_seq1	133	2.02	5616	104	-5.69	6.4E-13	4.9E-12	XP_003534655.1	cationic peroxidase 1-like [Glycine max]
Vr36176_c0_seq1	95	0.90	5444	63.3	-6.13	0	0	XP_003529158.1	leucoanthocyanidin dioxygenase-like [Glycine max]
Vr39450_c0_seq1	136	2.21	5138	102	-5.53	6.9E-13	5.2E-12	XP_003533952.1	21 kDa protein-like [Glycine max]
Vr13687_c0_seq1	77	0.47	4326	32.1	-6.10	0	0	XP_003531311.1	low affinity cationic amino acid transporter 2-like [Glycine max]
Vr42500_c0_seq1	156	1.32	4293	44.5	-5.07	1.3E-12	9.8E-12	XP_003547675.1	lysine histidine transporter 1-like [Glycine max]
Vr41972_c0_seq1	93	1.04	3151	43.1	-5.37	0	0	XP_003536550.1	thiazole biosynthetic enzyme, chloroplastic-like [Glycine max]
Vr42108_c0_seq1	6	0.11	3129	69.2	-9.32	0	0	NP_001238339.1	uncharacterized protein LOC100527589 [Glycine max]
Vr41271_c0_seq1	66	0.83	2487	38.4	-5.53	0	0	ACM89628.1	TIR-NBS-LRR type disease resistance protein [Glycine max]
Vr41725_c0_seq1	45	0.59	1929	31	-5.71	6.3E-14	5E-13	NP_001241518.1	uncharacterized protein LOC100788713 [Glycine max]
Vr36323_c0_seq1	35	0.31	1573	17	-5.78	4.5E-14	3.6E-13	XP_003552581.1	anthocyanin 5-aromatic acyltransferase-like [Glycine max]
Vr49784_c0_seq1	18	0.33	1553	35.1	-6.72	1.3E-14	1.1E-13	XP_003543377.1	cysteine-rich repeat secretory protein 38 [Glycine max]

Vr31555_c0_seq1	48	0.38	1542	14.9	-5.30	9.5E-14	7.5E-13	XP_003521608.1	sex determination protein tasselseed-2-like [Glycine max]
Vr13838_c0_seq1	11	0.08	1386	12.5	-7.27	0	0	XP_003518665.1	polygalacturonase At1g48100-like [Glycine max]
Vr41762_c0_seq1	1	0.01	1370	16.4	-10.71	2.2E-16	2E-15	XP_003556683.1	polygalacturonase-like [Glycine max]
Vr50416_c0_seq1	12	0.10	1313	13.3	-7.06	0	0	XP_003548705.1	D-inositol-3-phosphate glycosyltransferase-like [Glycine max]
Vr46027_c0_seq1	6	0.11	1094	24.4	-7.80	0	0	NP_001235010.1	uncharacterized protein LOC100500484 precursor [Glycine max]
Vr21159_c0_seq1	28	0.32	1088	15.1	-5.57	5.9E-14	4.7E-13	ABN08535.1	Auxin Efflux Carrier [Medicago truncatula]
Vr28474_c0_seq3	32	0.21	1074	8.72	-5.36	4.9E-14	3.9E-13	XP_003543367.1	equilibrative nucleoside transporter 3-like [Glycine max]
Vr49226_c0_seq1	3	0.03	1028	11.5	-8.71	0	0	XP_003525322.1	cytochrome P450 716B2-like [Glycine max]
Vr46582_c0_seq1	8	0.14	941	19.4	-7.17	0	0	NP_001241331.1	uncharacterized protein LOC100811719 precursor [Glycine max]
Vr34208_c0_seq1	9	0.23	889.5	27.4	-6.92	1.1E-15	9.6E-15	XP_003537598.1	isoflavone reductase-like [Glycine max]
Vr39778_c0_seq1	19	0.93	802	47.8	-5.69	1.8E-14	1.5E-13	XP_003552297.1	cationic peroxidase 1-like [Glycine max]
Vr35894_c0_seq1	18	0.15	699	7.02	-5.57	1.3E-14	1.1E-13	XP_003520743.1	probable pectinesterase/pectinesterase inhibitor 7-like [Glycine max]
Vr53527_c0_seq1	17	0.21	658	9.91	-5.56	5.6E-15	4.7E-14	XP_003538484.1	abscisic acid receptor PYL6-like [Glycine max]
Vr48273_c0_seq1	2.5	0.07	624	21.5	-8.25	1.6E-15	1.3E-14	BAA92940.1	yieldin precursor [Vigna unguiculata]
Vr45385_c0_seq1	14	0.11	617	5.71	-5.75	0	0	BAA19161.1	1-aminocyclopropane-1-carboxylate synthase [Vigna radiata var. radiata]
Vr45777_c0_seq1	22	0.31	609	10.4	-5.08	3.4E-14	2.7E-13	XP_003548263.1	4,5-DOPA dioxygenase extradiol-like protein-like [Glycine max]
Vr45152_c0_seq1	22	0.39	607	13.1	-5.08	3.4E-14	2.7E-13	XP_003527180.1	blue copper protein-like [Glycine max]
Vr44274_c0_seq1	12	0.58	605	35.6	-5.95	0	0	AAM12529.1	chalcone reductase [Pueraria montana var. lobata]
Vr50284_c0_seq1	4	0.05	534	8.41	-7.35	2.2E-16	2E-15	XP_003524361.1	expansin-like B1-like [Glycine max]
Vr59439_c0_seq1	2	0.02	522	6.85	-8.32	1.6E-15	1.3E-14	XP_003546286.1	reticuline oxidase-like protein-like [Glycine max]
Vr34971_c0_seq1	6195	96.16	1E+05	2679	-4.80	2.8E-10	1.6E-09	XP_003548287.1	trypsin inhibitor A-like [Glycine max]
Vr38156_c0_seq1	4981	36.71	91843	827	-4.49	3E-11	1.9E-10	ACC77194.1	isoflavone synthase 1 [Vigna unguiculata]
Vr36280_c0_seq2	4779	38.56	83481	823	-4.42	0	0	XP_003539343.1	anthocyanin 3'-O-beta-glucosyltransferase-like [Glycine max]

Top 50 DGEs con:wat6-down

Gene_ID	Reads1 -con	RPKM1	Reads2 -wat6	RPKM2	Log2	Pvalue	FDR	NR references	description
Vr22942_c0_seq1	209	2.20	0	0.00	NA	6.05E-55	9.57E-54	XP_003520521.1	polygalacturonase-like [Glycine max]
Vr175308_c0_seq1	197	7.32	0	0.00	NA	7.89E-52	1.22E-50	XP_003526623.1	auxin-induced protein 5NG4-like [Glycine max]
Vr108548_c0_seq1	176	11.43	0	0.00	NA	2.23E-46	3.27E-45	ABG56534.1|	polyprotein precursor [Bean common mosaic virus]
Vr123225_c0_seq1	167	11.23	0	0.00	NA	8.81E-44	1.25E-42	AGS41513.1	HC-Pro, partial [Bean common mosaic virus strain peanut stripe]
Vr754819_c0_seq1	107	1.17	0	0.00	NA	1.83E-28	2.11E-27	XP_003548659.1	transcription factor bHLH91-like [Glycine max]

Vr30105_c0_seq1	103	6.01	0	0.00	NA	2E-27	2.25E-26	XP_003541676.1	serine carboxypeptidase-like 12-like [Glycine max]
Vr39799_c0_seq1	21964	249.38	377	5.23	5.57	0	0	ABH02878.1	MYB transcription factor MYB134 [Glycine max]
Vr41217_c0_seq1	16245	142.63	474	5.09	4.81	0	0	ACJ61246.1	serine glyoxylate aminotransferase 2 [Glycine max]
Vr40489_c0_seq1	13321	223.86	200	4.11	5.77	0	0	NP_001236400.1|	MYB transcription factor MYB114 [Glycine max]
Vr38043_c0_seq1	12585	19.59	8	0.02	10.33	0	0	ACE95704.1	polyprotein precursor [Bean common mosaic virus]
Vr15096_c0_seq1	10976	70.99	364	2.88	4.62	0	0	XP_003544189.1	probable E3 ubiquitin-protein ligase HERC1-like [Glycine max]
Vr33063_c0_seq2	8706	28.73	270	1.09	4.72	0	0	XP_004512150.1	histidine kinase 1-like isoform X2 [Cicer arietinum]
Vr40796_c0_seq1	5243	57.69	153	2.06	4.81		0	XP_003549537.1	heat shock 70 kDa protein-like [Glycine max]
Vr50534_c0_seq1	4847	28.21	226	1.61	4.13	0	0	XP_003519092.1	ABC transporter G family member 22-like [Glycine max]
Vr13836_c0_seq2	4622	75.72	84	1.68	5.49	0	0	ABH02918.1	MYB transcription factor MYB114 [Glycine max]
Vr48206_c0_seq1	3644	65.60	153	3.37	4.28	0	0	XP_003523924.1	auxin-induced protein 5NG4-like [Glycine max]
Vr55469_c0_seq1	3578	27.63	74	0.70	5.31	0	0	XP_003537193.1	auxin-induced protein 5NG4-like isoform 1 [Glycine max]
Vr56588_c0_seq1	2173	16.46	72	0.67	4.63	0	0	AAO83155.1	putative organic cation transport protein [Phaseolus vulgaris]
Vr55489_c0_seq1	2101	23.85	97	1.35	4.15	0	0	XP_003529644.1	S-type anion channel SLAH1-like [Glycine max]
Vr24590_c0_seq1	2030	13.88	28	0.23	5.89	0	0	XP_003535586.1	S-type anion channel SLAH3-like [Glycine max]
Vr28255_c0_seq1	1388	11.09	39	0.38	4.86	4.8E-298	1.6E-296	XP_003539445.1	lysosomal Pro-X carboxypeptidase-like [Glycine max]
Vr14132_c0_seq1	1308	14.39	59	0.79	4.18	2.3E-256	7.2E-255	XP_002304053.1	predicted protein [Populus trichocarpa]
Vr42894_c0_seq1	1211	19.88	48	0.96	4.37	2.2E-244	6.9E-243	XP_003549537.1	heat shock 70 kDa protein-like [Glycine max]
Vr54849_c0_seq1	902	18.45	36	0.90	4.36	3.6E-182	9.8E-181	XP_003523141.1	calcium-binding protein CML38-like [Glycine max]
Vr37145_c0_seq1	808	7.32	24	0.27	4.78	1.3E-172	3.5E-171	NP_001236165.1	cytochrome P450 71D10 [Glycine max]
Vr27182_c0_seq1	806	6.18	21	0.20	4.97	9.6E-176	2.6E-174	XP_003529007.1	protein WAX2-like [Glycine max]
Vr112545_c0_seq1	753	12.39	14	0.28	5.46	1.3E-171	3.4E-170	XP_003529396.1	ethylene-responsive transcription factor ERF109-like [Glycine max]
Vr35442_c0_seq1	730	16.72	14	0.39	5.41	7.8E-166	2.1E-164	AAF81194.1	LEA-18 [Phaseolus vulgaris]
Vr50845_c0_seq1	726	16.18	15	0.41	5.31	1.8E-163	4.6E-162	XP_003531926.1	3-ketoacyl-CoA synthase 6-like [Glycine max]
Vr77281_c0_seq1	709	19.03	26	0.85	4.48	5.2E-146	1.3E-144	XP_003538774.1	nitrate transporter 1.5-like [Glycine max]
Vr51893_c0_seq1	694	19.39	25	0.85	4.51	1.9E-143	4.7E-142	ACZ51240.1	3-ketoacyl-CoA synthase [Arachis hypogaea]
Vr71314_c0_seq1	673	13.19	25	0.60	4.46	2.5E-138	6.2E-137	XP_003538534.1	cysteine proteinase inhibitor 2 [Glycine max]
Vr46055_c0_seq1	672	8.55	31	0.48	4.15	8.3E-132	2E-130	XP_003538574.1	small heat shock protein, chloroplastic-like [Glycine max]
Vr22084_c0_seq1	645	8.78	4	0.07	7.04	1.2E-159	3.1E-158	XP_003516973.1	UPF0098 protein MTH_273-like [Glycine max]
Vr15106_c0_seq1	602	15.34	30	0.93	4.04	4.9E-116	1.1E-114	NP_001241449.1	uncharacterized protein LOC100775900 [Glycine max]
Vr72294_c0_seq1	529	11.23	15	0.39	4.85	2.3E-114	5.2E-113	XP_003538774.1|	nitrate transporter 1.5-like [Glycine max]
Vr10403_c0_seq1	506	8.13	25	0.49	4.05	5.5E-98	1.15E-96	XP_003522798.1	sugar transport protein 5-like [Glycine max]
Vr32314_c0_seq1	146470	1096.47	9745	89.17	3.62	0	0	CAX94843.1	myo-inositol 1-phosphate synthase [Phaseolus vulgaris]

Vr28874_c0_seq9	73259	300.52	3932	19.72	3.93	0	0	XP_003528756.1	protein LHY [Glycine max]
Vr39139_c0_seq1	45984	283.81	3680	27.76	3.35	0	0	XP_003523384.1|	subtilisin-like protease-like [Glycine max]
Vr14424_c0_seq1	15783	88.89	1373	9.45	3.23	0	0	XP_003549515.1	protein WAX2-like [Glycine max]
Vr13806_c0_seq1	11403	74.71	1115	8.93	3.06	0	0	XP_003548314.1	uncharacterized protein LOC778089 [Glycine max]
Vr39861_c0_seq1	10124	242.94	1026	30.09	3.01	0	0	XP_003541070.1	21 kDa protein-like [Glycine max]
Vr43985_c0_seq1	9228	76.96	904	9.22	3.06	0	0	XP_003549204.1	putative nuclease HARBI1-like [Glycine max]
Vr43431_c0_seq1	8805	72.74	825	8.33	3.13	0	0	NP_001238594.1	syringolide-induced protein 13-1-1 [Glycine max]
Vr36823_c0_seq4	7049	41.46	702	5.05	3.04	0	0	XP_004492929.1	subtilisin-like protease-like isoform X1 [Cicer arietinum]
Vr29009_c0_seq6	6073	56.56	464	5.28	3.42	0	0	XP_003540767.1	uncharacterized protein LOC100797817 [Glycine max]
Vr38900_c0_seq1	5313	99.19	417	9.51	3.38	0	0	XP_003542731.1	18.5 kDa class I heat shock protein-like [Glycine max]
Vr32665_c0_seq2	5083	25.15	293	1.77	3.83	0	0	XP_003519894.1	phosphate transporter PHO1-like [Glycine max]
Vr36885_c0_seq1	4234	37.79	317	3.46	3.45	0	0	XP_003548043.1	isoflavone 2'-hydroxylase-like [Glycine max]
Vr47011_c0_seq1	4080	40.82	363	4.44	3.20	0	0	XP_003524651.1	transcription factor HEC1-like [Glycine max]
Vr50091_c0_seq1	3891	55.39	304	5.29	3.39	0	0	XP_003533795.1	ethylene-responsive transcription factor ERF017-like [Glycine max]
Vr22806_c0_seq1	3879	36.92	334	3.89	3.25	0	0	XP_003534929.1	putative nuclease HARBI1-like [Glycine max]
Vr44946_c0_seq1	3251	32.73	328	4.04	3.02	0	0	XP_003544508.1	homeobox-leucine zipper protein HOX19-like [Glycine max]
Vr17354_c0_seq1	2317	22.31	232	2.73	3.03	0	0	XP_003545292.1	phytoene synthase, chloroplastic-like [Glycine max]

Top 50 DGEs wat24:wat6-Down

Gene_ID	Reads1-wat6	RPKM1	Reads 2-wat 24	RPKM2	Log2	P-value	FDR	NR	
Vr41972_c0_seq1	3151	43.11	10	0.14	8.30	0	0	XP_003536550.1	thiazole biosynthetic enzyme, chloroplastic-like [Glycine max]
Vr13298_c0_seq1	2934	61.12	83	1.73	5.14	0	0	XP_003594345.1	hypothetical protein MTR_2g027550 [Medicago truncatula]
Vr49846_c0_seq1	2439	19.96	74	0.61	5.04	0	0	NP_001235886.1	circadian clock-associated FKF1 [Glycine max]
Vr34247_c0_seq1	1056	23.76	30	0.68	5.14	5.3E-269	1.7E-267	NP_001239781.1	uncharacterized protein LOC100782877 [Glycine max]
Vr45385_c0_seq1	617	5.71	6	0.06	6.68	2.8E-174	8.5E-173	BAA19161.1	1-aminocyclopropane-1-carboxylate synthase [Vigna radiata var. radiata]
Vr21502_c0_seq1	561	9.02	6	0.10	6.55	1.1E-157	3.4E-156	XP_003536475.1	uncharacterized protein LOC100792832 [Glycine max]
Vr13406_c0_seq1	101800	1268.32	4898	61.06	4.38	0	0	ACZ74696.1	formate dehydrogenase [Phaseolus vulgaris]
Vr22757_c0_seq3	11699	187.39	620	9.94	4.24	0	0	NP_001235540.1	uncharacterized protein LOC100305960 [Glycine max]
Vr38378_c0_seq1	9249	205.20	375	8.33	4.62	0	0	AAY51975.1	GIR1 [Vigna radiata]
Vr40132_c0_seq1	7291	157.37	334	7.21	4.45	0	0	XP_003551324.1	uncharacterized protein LOC100811254 [Glycine max]
Vr41355_c0_seq1	6670	66.93	302	3.03	4.46	0	0	XP_003547247.1	beta-glucosidase 47-like [Glycine max]

Vr32306_c0_seq1	3766	51.45	160	2.19	4.56	0	0	NP_001242314.1	uncharacterized protein LOC100820588 [Glycine max]
Vr45510_c0_seq1	1515	18.91	54	0.67	4.81	0	0	XP_003521784.1	GDSL esterase/lipase At5g33370-like [Glycine max]
Vr42551_c0_seq1	1050	20.68	33	0.65	4.99	1.4E-263	4.4E-262	NP_001238664.1	uncharacterized protein LOC100500218 [Glycine max]
Vr67595_c0_seq1	868	6.95	52	0.42	4.06	6.2E-192	1.9E-190	XP_003526280.1	probable L-type lectin-domain containing receptor kinase VII.2-like [Glycine max]
Vr53207_c0_seq1	843	21.33	50	1.27	4.07	6.2E-187	1.9E-185	XP_003546144.1	15.4 kDa class V heat shock protein-like [Glycine max]
Vr10963_c0_seq2	696	7.86	40	0.45	4.12	7.6E-156	2.2E-154	XP_003531287.1	UDP-glycosyltransferase 74F1-like [Glycine max]
Vr20800_c0_seq2	519	8.79	30	0.51	4.11	1.8E-116	5.3E-115	XP_003548408.1	uncharacterized protein LOC100791528 [Glycine max]
Vr34550_c0_seq2	29551	394.94	2952	39.48	3.32	0	0	AAW27915.1	xyloglucan endotransglucosylase/hydrolase precursor [Vigna radiata]
Vr23172_c0_seq1	21227	223.54	1912	20.15	3.47	0	0	XP_003518844.1	benzyl alcohol O-benzoyltransferase-like [Glycine max]
Vr37396_c0_seq1	18259	190.19	1665	17.35	3.45	0	0	XP_003520588.1	LOW QUALITY PROTEIN: cytochrome P450 94A1-like [Glycine max]
Vr36176_c0_seq1	5444	63.33	478	5.56	3.51	0	0	XP_003529158.1	leucoanthocyanidin dioxygenase-like [Glycine max]
Vr42461_c0_seq1	5259	73.63	531	7.44	3.31	0	0	XP_003546057.1	GDSL esterase/lipase At1g29670-like [Glycine max]
Vr22359_c0_seq1	4255	50.17	404	4.77	3.40	0	0	XP_003531568.1	auxin-induced protein 5NG4-like [Glycine max]
Vr36822_c0_seq1	3653	40.93	426	4.78	3.10	0	0	AAA87182.1	auxin-induced protein [Vigna radiata]
Vr22565_c0_seq2	3414	22.05	423	2.73	3.01	0	0	ACU23356.1	unknown [Glycine max]
Vr44347_c0_seq1	2886	40.20	301	4.20	3.26	0	0	XP_003520449.1	leucoanthocyanidin dioxygenase-like [Glycine max]
Vr40851_c0_seq1	2437	85.99	206	7.27	3.56	0	0	XP_003522964.1	uncharacterized protein LOC100500383 [Glycine max]
Vr46260_c0_seq1	2070	28.75	201	2.79	3.36	0	0	XP_003534534.1	7-methylxanthosine synthase 1-like [Glycine max]
Vr42547_c0_seq1	1939	80.97	170	7.10	3.51	0	0	AAK18751.1|	patatin-like protein [Vigna unguiculata]
Vr45762_c0_seq1	1747	28.34	214	3.47	3.03	1E-298	3.3E-297	NP_001240893.1	uncharacterized protein LOC100790141 [Glycine max]
Vr52177_c0_seq1	1700	24.63	125	1.81	3.76	0	0	NP_001248360.1	expansin-B3-like precursor [Glycine max]
Vr41278_c0_seq1	1682	83.99	119	5.95	3.82	0	0	NP_001237144.1	uncharacterized protein LOC100305819 precursor [Glycine max]
Vr39198_c0_seq1	1659	43.77	181	4.78	3.20	6.3E-299	2E-297	O24541.1	Auxin-induced protein 22C; Aux22c [Vigna radiata]
Vr47043_c0_seq1	1597	39.72	106	2.64	3.91	0	0	XP_003537630.1	auxin-induced protein 15A-like [Glycine max]
Vr40704_c0_seq1	1571	64.61	160	6.58	3.29	2.3E-291	7.4E-290	AAK18751.1	patatin-like protein [Vigna unguiculata]
Vr39142_c0_seq1	1254	45.41	136	4.94	3.20	9.9E-227	3.1E-225	O24541.1	Auxin-induced protein 22C; AltName: Aux22c [Vigna radiata]
Vr30665_c0_seq1	1211	15.24	128	1.61	3.24	1.2E-221	3.7E-220	XP_003518070.1	uncharacterized protein LOC100809248 [Glycine max]
Vr42706_c0_seq1	1131	17.64	81	1.26	3.80	1.5E-237	4.7E-236	XP_004494855.1	uncharacterized protein LOC101493620 [Cicer arietinum]
Vr46027_c0_seq1	1094	24.41	80	1.79	3.77	2.1E-228	6.5E-227	NP_001235010.1	uncharacterized protein LOC100500484 precursor [Glycine max]
Vr20019_c0_seq1	1082	7.32	72	0.49	3.91	3.3E-232	1E-230	XP_003528000.1	two-Vronent response regulator-like PRR95-like [Glycine max]

Vr37789_c0_seq1	981	21.08	86	1.85	3.51	3.2E-193	9.9E-192	XP_003534344.1	auxin-induced protein 10A5-like [Glycine max]
Vr34208_c0_seq1	890	27.39	92	2.84	3.27	1.3E-164	3.9E-163	XP_003537598.1	isoflavone reductase-like [Glycine max]
Vr51018_c0_seq1	862	17.80	100	2.07	3.11	4.4E-152	1.3E-150	XP_003528935.1	uncharacterized protein LOC100795735 [Glycine max]
Vr36836_c0_seq3	829	19.60	82	1.95	3.33	2.1E-156	6.3E-155	XP_003533487.1	auxin-induced protein X10A-like [Glycine max]
Vr22989_c0_seq1	730	8.35	75	0.86	3.28	7E-136	2E-134	XP_003517852.1	UDP-glycosyltransferase 74B1-like [Glycine max]
Vr37739_c0_seq2	714	15.46	86	1.86	3.05	3.9E-124	1.1E-122	ADI17229.1	hypothetical protein [uncultured alpha proteobacterium HF0070_14E07]
Vr57190_c0_seq1	697	9.94	52	0.74	3.74	2.9E-145	8.5E-144	XP_003544885.1	uncharacterized protein LOC100793086 [Glycine max]
Vr22629_c0_seq1	658	12.22	64	1.19	3.36	2.6E-125	7.5E-124	XP_003530613.1	uncharacterized protein LOC100500563 [Glycine max] gb|ACU15678.1| unknown [Glycine max]
Vr33294_c0_seq1	573	11.63	59	1.20	3.28	6.3E-107	1.7E-105	XP_003535935.1	calvin cycle protein CP12-like [Glycine max]
Vr44933_c0_seq1	513	10.01	40	0.78	3.68	6.8E-106	1.9E-104	XP_003542633.1	auxin-responsive protein IAA12-like [Glycine max]
Vr34844_c1_seq1	61856	154.98	14781	37.06	2.06	0	0	XP_003637074.1	Cell wall-associated hydrolase, partial [Medicago truncatula]
Vr38305_c0_seq1	32326	597.89	7981	147.71	2.02	0	0	P14298.2	Chalcone--flavonone isomerase; [Phaseolus vulgaris]
Vr38333_c0_seq1	31984	537.03	4339	72.90	2.88	0	0	XP_003539812.1	uncharacterized protein At5g22580-like [Glycine max]
Vr38231_c0_seq1	29772	550.12	5015	92.73	2.57	0	0	NP_001235010.1	uncharacterized protein LOC100500484 precursor [Glycine max]
Vr38502_c0_seq1	21672	302.31	4920	68.68	2.14	0	0	XP_003549741.1	uncharacterized protein LOC100801982 [Glycine max]

Top 50 DGEs Wat24:Wat6-Up

Gene_ID	Reads 1-wat 6	RPKM1	Reads2-wat24	RPKM2	Log2	P-value	FDR	NR	Description
Vr68124_c0_seq1	0	0.00	988	22.01	NA	0	0	P0DI41.1	VIGUN Casparian strip membrane protein 3
Vr68249_c0_seq1	0	0.00	705	18.66	NA	0	0	AFK36631.1	unknown [Medicago truncatula]
Vr41572_c0_seq1	0	0.00	438	5.81	NA	0	0	XP_003554607.1	auxin-induced protein 5NG4-like [Glycine max]
Vr51177_c0_seq1	0	0.00	244	4.99	NA	0	0	XP_003528524.1	auxin-binding protein ABP19a-like [Glycine max]
Vr19710_c0_seq1	0	0.00	205	3.07	NA	0	0	CBJ28620.1	Ribosomal protein L3 [Ectocarpus siliculosus]
Vr36832_c0_seq1	0	0.00	188	3.01	NA	0	0	CBJ31444.1	Cathepsin L-like proteinase [Ectocarpus siliculosus]
Vr17981_c0_seq1	0	0.00	177	4.02	NA	0	0	NP_001236648.1	CASP-like protein 1 [Glycine max]
Vr118414_c0_seq	0	0.00	175	2.90	NA	0	0	XP_003528650.1	ethylene-responsive transcription factor ERF086-like [Glycine max]
Vr404398_c0_seq	0	0.00	141	2.81	NA	0	0	NP_001239790.1	uncharacterized protein LOC100778526 precursor [Glycine max]
Vr44673_c0_seq1	15	0.28	3255	61.19	-7.76	2.66E-15	4.34E-14	XP_003539771.1	vignain-like [Glycine max]
Vr22850_c0_seq1	14	0.16	2822	32.99	-7.66	0	0	NP_001241330.1	uncharacterized protein LOC100808391 [Glycine max]
Vr34411_c0_seq1	20	0.21	2782	28.56	-7.12	2.35E-14	3.69E-13	XP_003521576.1	endoglucanase 17-like [Glycine max]

Vr35020_c0_seq1	7	0.11	1955	31.52	-8.13	0	0	XP_003539707.1	uncharacterized protein LOC100784571 [Glycine max]
Vr59584_c0_seq1	7	0.12	1296	21.83	-7.53	0	0	XP_003543376.1	early nodulin-like protein 1-like [Glycine max]
Vr36698_c0_seq1	4	0.09	1294	29.64	-8.34	0	0	P0DI40.1	VIGUNCasparian strip membrane protein 2
Vr22476_c0_seq1	15	0.16	1023	10.96	-6.09	2.66E-15	4.34E-14	XP_003556105.1	low-temperature-induced 65 kDa protein-like [Glycine max]
Vr35419_c0_seq1	20	0.26	972	12.57	-5.60	2.35E-14	3.69E-13	AAU94657.	ef1a [Stramenopile sp. ex Nuclearia delicatula CCAP1552/1]
Vr58105_c0_seq1	2	0.04	951	16.99	-8.89	0	0	XP_003555719.1	uncharacterized protein LOC100810925 [Glycine max]
Vr35442_c0_seq1	14	0.39	868	24.32	-5.96	0	0	AAF81194.1	LEA-18 [Phaseolus vulgaris]
Vr100707_c0_seq	4	0.02	511	2.29	-7.00	0	0	XP_003524132.1	LRR receptor-like serine/threonine-protein kinase GSO1-like [Glycine max]
Vr22680_c0_seq1	1892	23.45	34863	432.38	-4.20	0	0	XP_003529031.1	patatin group A-3-like [Glycine max]
Vr39095_c0_seq1	1149	14.01	25010	305.24	-4.44	0	0	XP_003554078.1	metacaspase-9-like [Glycine max]
Vr39799_c0_seq1	377	5.23	8130	112.91	-4.43	7.58E-12	9.08E-11	ABH02878.1	MYB transcription factor MYB134 [Glycine max]
Vr15096_c0_seq1	364	2.88	6605	52.25	-4.18	7.32E-12	8.83E-11	XP_003544189.1	probable E3 ubiquitin-protein ligase HERC1-like [Glycine max]
Vr43029_c0_seq1	197	6.19	5100	160.26	-4.70	2.64E-13	3.86E-12	XP_003529031.1	patatin group A-3-like [Glycine max]
Vr23696_c0_seq1	243	1.69	4951	34.48	-4.35	1.6E-12	2.11E-11	XP_003547234.1	uncharacterized protein LOC100782145 [Glycine max]
Vr40489_c0_seq1	200	4.11	4616	94.88	-4.53	6.08E-13	8.64E-12	NP_001236400.1	MYB transcription factor MYB114 [Glycine max] gb|ABW87009.1| late elongated hypocotyl and circadian clock associated-1-like protein 2 [Glycine max]
Vr40796_c0_seq1	153	2.06	4450	59.89	-4.86	8.39E-13	1.16E-11	XP_003549537.1	heat shock 70 kDa protein-like [Glycine max]
Vr24999_c1_seq1	90	2.05	2396	54.52	-4.74	0	0	XP_003534948.1	protein LURP-one-related 6-like [Glycine max]
Vr53159_c0_seq1	90	0.80	1914	16.95	-4.41	0	0	XP_003539873.1	isocitrate lyase 2-like [Glycine max]
Vr42894_c0_seq1	48	0.96	1432	28.76	-4.90	6.73E-14	1.01E-12	XP_003549537.1	heat shock 70 kDa protein-like [Glycine max]
Vr13836_c0_seq2	84	1.68	1369	27.43	-4.03	0	0	ABH02918.1	MYB transcription factor MYB114 [Glycine max]
Vr51789_c0_seq1	65	1.11	1125	19.15	-4.11	0	0	XP_003540338.1	protein THYLAKOID FORMATION1, chloroplastic-like [Glycine max]
Vr24590_c0_seq1	28	0.23	512	4.28	-4.19	5.37E-14	8.15E-13	XP_003535586.1	S-type anion channel SLAH3-like [Glycine max]
Vr38255_c0_seq1	9577	64.00	85379	570.89	-3.16	0	0	AAA92063.1	cysteinyl endopeptidase [Vigna radiata]
Vr38664_c0_seq1	3003	43.67	31249	454.75	-3.38	1.01E-10	9.98E-10	XP_003540200.1	serine carboxypeptidase-like 48-like [Glycine max]
Vr36096_c0_seq1	2660	24.31	29102	266.09	-3.45	0	0	XP_003544396.1	galactose oxidase-like [Glycine max]
Vr36446_c0_seq1	1372	7.64	15451	86.10	-3.49	0	0	XP_003516513.1	subtilisin-like protease-like [Glycine max]
Vr14990_c0_seq1	1128	8.55	15322	116.17	-3.76	0	0	XP_003555206.1	hippocampus abundant transcript 1 protein-like [Glycine max]
Vr39702_c0_seq1	1066	14.00	14742	193.76	-3.79	0	0	XP_003519813.1	auxin-induced protein 5NG4-like [Glycine max]
Vr34889_c0_seq1	1143	19.33	13312	225.24	-3.54	0	0	P29024.1	Acidic endochitinase; Flags: Precursor
Vr13808_c0_seq1	579	8.82	8271	126.02	-3.84	1.81E-11	2E-10	NP_001235325.1	endonuclease [Glycine max]
Vr34289_c0_seq2	698	11.61	7644	127.20	-3.45	3.47E-12	4.4E-11	XP_002523505.1	apolipoprotein d, putative [Ricinus communis]

Vr41941_c0_seq1	591	6.27	4983	52.92	-3.08	1.82E-11	2E-10	XP_003533818.1	CBL-interacting serine/threonine-protein kinase 1-like [Glycine max]
Vr41217_c0_seq1	474	5.09	4775	51.28	-3.33	1.02E-11	1.18E-10	ACJ61246.1	serine glyoxylate aminotransferase 2 [Glycine max]
Vr35961_c0_seq6	295	1.18	4115	16.47	-3.80	2.32E-12	2.99E-11	XP_003527521.1	probable nucleoredoxin 2-like isoform 2 [Glycine max]
Vr38900_c0_seq1	417	9.51	4064	92.80	-3.29	7.17E-12	8.68E-11	XP_003542731.1	18.5 kDa class I heat shock protein-like [Glycine max]
Vr39642_c0_seq1	446	17.81	3633	145.14	-3.03	9.97E-12	1.15E-10	XP_003540200.1	serine carboxypeptidase-like 48-like [Glycine max]
Vr13830_c0_seq1	324	3.62	3295	36.83	-3.35	4.18E-12	5.26E-11	XP_003547855.1	probable calcium-binding protein CML16-like [Glycine max]
Vr13152_c0_seq1	256	3.14	3028	37.17	-3.57	2.32E-12	2.98E-11	XP_003535208.1	thaumatin-like protein 1a-like [Glycine max]
Vr46251_c0_seq1	266	2.62	2892	28.53	-3.44	1.68E-12	2.21E-11	XP_003517028.1	pectinesterase 2-like [Glycine max]
Vr40741_c0_seq1	305	3.90	2650	33.94	-3.12	4.1E-12	5.16E-11	XP_003526939.1	bidirectional sugar transporter SWEET12-like [Glycine max]
Vr35212_c0_seq3	191	1.28	2487	16.73	-3.70	4.11E-13	5.94E-12	XP_003552931.1	laccase-3-like [Glycine max]
Vr46167_c0_seq1	165	2.05	2086	25.96	-3.66	5.3E-13	7.57E-12	XP_003541076.1	UPF0496 protein At4g34320-like [Glycine max]
Vr13168_c0_seq1	131	2.33	2065	36.83	-3.98	1.08E-12	1.48E-11	BAB33036.1	CPRD49 [Vigna unguiculata]
Vr44076_c0_seq1	161	2.35	1710	24.96	-3.41	9.11E-13	1.26E-11	XP_003546306.1	desiccation-related protein PCC13-62-like [Glycine max]

Top 50 DGEs Con :Wat24 -Up

Gene_ID	Reads1	RPKM1	Reads2	RPKM2	Log2	P-value	FDR	NR	Description
Vr43029_c0_seq1	0	0	5100	160.26	NA	4.4E-16	4.4E-15	XP_003529031.1	patatin group A-3-like [Glycine max]
Vr39448_c0_seq1	0	0	4245	234.11	NA	4.4E-16	4.4E-15	XP_004500339.1	cationic peroxidase 1-like [Cicer arietinum]
Vr39419_c0_seq1	0	0	3670	79.1797	NA	4.4E-16	4.4E-15	ACU24511.1	unknown [Glycine max]
Vr44006_c0_seq1	0	0	2547	51.0384	NA	4.4E-16	4.4E-15	BAJ22384.1	terminal flower 1a [Vigna unguiculata]
Vr58791_c0_seq1	0	0	1221	15.6191	NA	4.4E-16	4.4E-15	XP_003529031.1	patatin group A-3-like [Glycine max]
Vr58732_c0_seq1	0	0	787	12.9499	NA	4.4E-16	4.4E-15	XP_003551178.1	cationic peroxidase 1-like [Glycine max]
Vr114032_c0_seq1	0	0	711	14.8541	NA	4.4E-16	4.4E-15	gb|ACG69488.1	germin-like protein 12 [Glycine max]
Vr65533_c0_seq1	0	0	536	7.87212	NA	4.4E-16	4.4E-15	XP_003518150.1	ethylene-responsive transcription factor LEP-like [Glycine max]
Vr145400_c0_seq1	0	0	420	9.2919	NA	4.4E-16	4.4E-15	ABP52096.1	phosphate transporter [Phaseolus vulgaris]
Vr69119_c0_seq1	0	0	254	3.91682	NA	4.4E-16	4.4E-15	XP_003520006.1	probable pectinesterase 29-like [Glycine max]
Vr51177_c0_seq1	0	0	244	4.99401	NA	4.4E-16	4.4E-15	XP_003528524.1	auxin-binding protein ABP19a-like [Glycine max]
Vr185592_c0_seq1	0	0	237	8.84102	NA	4.4E-16	4.4E-15	NP_001241164.1	phosphate transporter 1-7-like [Glycine max]
Vr48482_c0_seq1	0	0	207	9.75698	NA	4.4E-16	4.4E-15	XP_003551393.1	probable LRR receptor-like serine/threonine-protein kinase At4g26540-like isoform 1 [Glycine max]
Vr37067_c0_seq4	0	0	168	1.56982	NA	4.4E-16	4.4E-15	XP_003548678.1	G-type lectin S-receptor-like serine/threonine-protein kinase At1g11330-like [Glycine max]
Vr64026_c0_seq1	0	0	163	6.82563	NA	4.4E-16	4.4E-15	XP_003551603.1|	denine/guanine permease AZG1-like [Glycine max]
Vr12323_c0_seq1	0	0	144	2.91301	NA	4.4E-16	4.4E-15	XP_003545985.1	polygalacturonase-like [Glycine max]
Vr10342_c0_seq1	0	0	141	1.62646	NA	4.4E-16	4.4E-15	XP_003519899.1|	cytochrome P450 90B1-like [Glycine max]

Vr66297_c0_seq1	0	0	135	11.9054	NA	4.4E-16	4.4E-15	P34952.1	Chymotrypsin inhibitor ECI
Vr16293_c0_seq1	0	0	130	2.88943	NA	4.4E-16	4.4E-15	NP_001242396.1	uncharacterized protein LOC100812163 precursor [Glycine max]
Vr160743_c0_seq1	0	0	102	1.58438	NA	4.4E-16	4.4E-15	XP_003541980.1	transcription factor FER-LIKE IRON DEFICIENCY-INDUCED TRANSCRIPTION FACTOR-like [Glycine max]
Vr34521_c0_seq2	2822	47.5	1E+05	2117.22	-5.477	2.7E-11	1.8E-10	AAA66288.1	proline-rich protein [Glycine max]
Vr35851_c0_seq1	825	11.6	46041	791.627	-6.093	0	0	NP_001237786.1	Kunitz trypsin protease inhibitor precursor [Glycine max]
Vr22610_c0_seq1	13	0.19	20516	372.845	-10.91	0	0	XP_003544327.1	cationic peroxidase 1-like [Glycine max]
Vr31128_c0_seq2	366	4.2	16176	226.783	-5.756	4.8E-12	3.6E-11	XP_003547616.1	cationic peroxidase 2-like [Glycine max]
Vr34889_c0_seq1	154	2.13	13312	225.243	-6.724	1.4E-12	1.1E-11	P29024.1	Acidic endochitinase; Flags: Precursor
Vr39225_c0_seq1	133	2.02	12040	223.697	-6.791	6.3E-13	5.2E-12	XP_003534655.1	cationic peroxidase 1-like [Glycine max]
Vr38890_c0_seq1	138	1.28	10070	114.164	-6.48	7.6E-13	6.2E-12	XP_003536300.1	7-ethoxycoumarin O-deethylase-like [Glycine max]
Vr39450_c0_seq1	136	2.21	8109	161.311	-6.188	6.8E-13	5.6E-12	XP_003533952.1	21 kDa protein-like [Glycine max]
Vr13838_c0_seq1	11	0.08	7752	70.0421	-9.752	0	0	XP_003518665.1	polygalacturonase At1g48100-like [Glycine max]
Vr39180_c0_seq1	2	0.04	7480	166.842	-12.16	4.4E-16	4.4E-15	XP_003538325.1	cationic peroxidase 1-like [Glycine max]
Vr34333_c0_seq1	170	2	7123	102.645	-5.679	6E-13	5E-12	NP_001236116.1	uncharacterized protein LOC100500048 precursor [Glycine max]
Vr36526_c0_seq1	174.5	6.49	5962	270.993	-5.385	5.6E-13	4.7E-12	ABS70717.1	pathogen-related protein [Vigna angularis]
Vr39755_c0_seq1	92	0.86	5637	64.1347	-6.228	0	0	NP_001238091.1	polygalacturonase PG1 precursor [Glycine max]
Vr40472_c0_seq1	109	1.22	4060	55.656	-5.51	0	0	CAA61279.1	basic chitinase class 3 [Vigna unguiculata]
Vr39039_c0_seq1	52	0.82	3989	76.9527	-6.552	6.3E-14	5.5E-13	ADX66343.1	pathogenesis-related protein [Glycine max]
Vr44673_c0_seq1	82	1.26	3255	61.1892	-5.601	0	0	XP_003539771.1	vignain-like [Glycine max]
Vr46251_c0_seq1	30	0.24	2892	28.5278	-6.882	5.4E-14	4.8E-13	XP_003517028.1	pectinesterase 2-like [Glycine max]
Vr22850_c0_seq1	88	0.84	2822	32.9898	-5.294	0	0	NP_001241330.1	uncharacterized protein LOC100808391 [Glycine max]
Vr34411_c0_seq1	6	0.05	2782	28.5616	-9.148	0	0	XP_003521576.1	endoglucanase 17-like [Glycine max]
Vr41032_c0_seq1	51	0.79	2736	51.6866	-6.036	8E-14	7E-13	XP_003535070.1	peroxidase C3-like isoform 2 [Glycine max]
Vr31555_c0_seq1	48	0.38	2186	21.0853	-5.8	9.5E-14	8.2E-13	XP_003521608.1	sex determination protein tasselseed-2-like [Glycine max]
Vr40216_c0_seq1	47	1.2	2120	66.183	-5.786	8.7E-14	7.6E-13	XP_003535071.1	peroxidase C3-like isoform 3 [Glycine max]
Vr42825_c0_seq1	47	0.68	2087	36.8778	-5.763	8.7E-14	7.6E-13	XP_003542895.1	endo-1,3;1,4-beta-D-glucanase-like [Glycine max]
Vr35020_c0_seq1	50	0.66	1955	31.5186	-5.58	1E-13	8.7E-13	XP_003539707.1	uncharacterized protein LOC100784571 [Glycine max]
Vr41271_c0_seq1	66	0.83	1918	29.6483	-5.152	0	0	ACM89628.1	TIR-NBS-LRR type disease resistance protein [Glycine max]
Vr42199_c0_seq1	14	0.15	1858	23.72	-7.343	0	0	XP_003528650.1	ethylene-responsive transcription factor ERF086-like [Glycine max]
Vr41762_c0_seq1	1	0.01	1669	20.0373	-11	8.9E-16	8.6E-15	XP_003556683.1	polygalacturonase-like [Glycine max]
Vr45152_c0_seq1	22	0.39	1579	33.99	-6.456	3.6E-14	3.3E-13	XP_003527180.1	blue copper protein-like [Glycine max]
Vr39778_c0_seq1	19	0.93	1532	91.3324	-6.624	1.7E-14	1.6E-13	XP_003552297.1	cationic peroxidase 1-like [Glycine max]

Vr46582_c0_seq1	8	0.14	1486	30.6768	-7.828	0	0	NP_001241331.1	uncharacterized protein LOC100811719 precursor [Glycine max]
Vr59584_c0_seq1	43	0.59	1296	21.8322	-5.204	7.3E-14	6.4E-13	XP_003543376.1	early nodulin-like protein 1-like [Glycine max]
Vr36698_c0_seq1	34	0.64	1294	29.645	-5.54	4.6E-14	4.1E-13	P0DI40.1	Casparian strip membrane protein 2
Vr50416_c0_seq1	12	0.1	1158	11.7687	-6.883	0	0	XP_003548705.1	D-inositol-3-phosphate glycosyltransferase-like [Glycine max]
Vr18948_c0_seq1	18	0.11	1081	7.77121	-6.199	1.1E-14	1E-13	XP_003553810.1	potassium transporter 5-like [Glycine max]
Vr44815_c0_seq1	37	0.24	1059	8.48303	-5.13	5.1E-14	4.5E-13	XP_003529133.1	laccase-9-like [Glycine max]

Top 50 DGEs Con :Wat24 -Down

Gene_ID	Length	Reads1	RPKM1	Reads2	RPKM2	Log2	P-value	FDR	NR	Description
Vr12598_c0_seq1	1156	506	6.85	0	0.00	NA	5.5E-132	1.6E-130	XP_003517303.1	transcription factor HEC1-like [Glycine max]
Vr108548_c0_seq1	241	176	11.43	0	0.00	NA	2.35E-46	4.2E-45	ABG56534.1	polyprotein precursor [Bean common mosaic virus]
Vr123225_c0_seq1	232	167	11.23	0	0.00	NA	9.25E-44	1.6E-42	AGS41513.1	HC-Pro, partial [Bean common mosaic virus strain peanut stripe]
Vr20601_c0_seq1	742	158	3.32	0	0.00	NA	2E-41	3.37E-40	XP_004509731.1	uncharacterized protein LOC101508712 [Cicer arietinum]
Vr34044_c0_seq3	587	116	3.09	0	0.00	NA	8.72E-31	1.26E-29	XP_004492294.1	uncharacterized protein LOC101515081 [Cicer arietinum]
Vr28706_c0_seq1	250	111	6.95	0	0.00	NA	1.73E-29	2.45E-28	AAX82171.1	polyprotein [Bean common mosaic virus strain peanut stripe]
Vr748356_c0_seq1	1589	105	1.03	0	0.00	NA	6.24E-28	8.6E-27	XP_004510150.1	protein ECERIFERUM 1-like [Cicer arietinum]
Vr30105_c0_seq1	268	103	6.01	0	0.00	NA	2.06E-27	2.81E-26	XP_003541676.1	serine carboxypeptidase-like 12-like [Glycine max]
Vr533222_c0_seq1	999	101	1.58	0	0.00	NA	6.81E-27	9.18E-26	XP_003542039.1	laccase-17-like [Glycine max]
Vr38043_c0_seq1	10052	12585	19.59	63	0.12	7.35	0	0	ACE95704.1	polyprotein precursor [Bean common mosaic virus]
Vr48206_c0_seq1	869	3644	65.60	70	1.54	5.41	0	0	XP_003523924.1	auxin-induced protein 5NG4-like [Glycine max]
Vr27182_c0_seq1	2039	806	6.18	8	0.08	6.36	6.2E-194	2E-192	XP_003529007.1	protein WAX2-like [Glycine max]
Vr72740_c0_seq1	1426	729	8.00	18	0.24	5.05	3E-160	9.1E-159	XP_003535206.1	peptide transporter PTR3-A-like [Glycine max]
Vr72751_c0_seq1	1496	699	7.31	17	0.22	5.07	4.8E-154	1.5E-152	XP_003536954.1	GDSL esterase/lipase At4g28780-like [Glycine max]
Vr22359_c0_seq1	1622	11841	114.22	404	4.77	4.58	0	0	XP_003531568.1	auxin-induced protein 5NG4-like [Glycine max]
Vr38378_c0_seq1	862	7422	134.71	375	8.33	4.02	0	0	AAY51975.1	GIR1 [Vigna radiata]
Vr13211_c1_seq1	1273	5205	63.97	244	3.67	4.12	0	0	XP_003550171.1	uncharacterized protein LOC100813910 [Glycine max]
Vr55469_c0_seq1	2026	3578	27.63	141	1.33	4.37	0	0	XP_003537193.1	auxin-induced protein 5NG4-like isoform 1 [Glycine max]
Vr45510_c0_seq1	1532	1959	20.01	54	0.67	4.89	0	0	XP_003521784.1	GDSL esterase/lipase At5g33370-like [Glycine max]
Vr101604_c0_seq1	1616	802	7.76	33	0.39	4.31	5.2E-161	1.6E-159	XP_003554335.1	probable pectate lyase 3-like [Glycine max]
Vr112545_c0_seq1	951	753	12.39	34	0.68	4.18	3.7E-148	1.1E-146	XP_003529396.1	ethylene-responsive transcription factor ERF109-like [Glycine max]
Vr57343_c0_seq1	405	673	26.00	33	1.56	4.06	5.5E-130	1.6E-128	NP_001236386.1	uncharacterized protein LOC100306533 [Glycine max]
Vr30607_c0_seq1	1539	639	6.50	26	0.32	4.33	6.6E-129	1.9E-127	XP_003553275.1	GDSL esterase/lipase At5g33370-like [Glycine max]
Vr72294_c0_seq1	737	529	11.23	23	0.60	4.23	1.9E-105	5E-104	XP_003538774.1	nitrate transporter 1.5-like [Glycine max]
Vr34550_c0_seq2	1431	40042	437.80	2952	39.48	3.47	0	0	AAW27915.1	xyloglucan endotransglucosylase/hydrolase precursor [Vigna radiata]

Vr31512_c0_seq1	1405	8929	99.43	661	9.00	3.47	0	0	XP_003533364.1	transcription factor BEE 3-like [Glycine max]
Vr44566_c0_seq1	1467	6118	65.25	396	5.17	3.66	0	0	NP_001241603.1	uncharacterized protein LOC100812537 [Glycine max]
Vr36885_c0_seq1	1753	4234	37.79	421	4.60	3.04	0	0	XP_003548043.1	isoflavone 2'-hydroxylase-like [Glycine max]
Vr39383_c0_seq1	806	4139	80.34	401	9.52	3.08	0	0	XP_003553069.1	uncharacterized protein LOC100788078 [Glycine max]
Vr30665_c0_seq1	1520	1694	17.44	128	1.61	3.44	8E-285	2.9E-283	XP_003518070.1	uncharacterized protein LOC100809248 [Glycine max]
Vr44134_c0_seq1	1127	1520	21.10	132	2.24	3.23	2E-242	6.8E-241	XP_003534342.1	indole-3-acetic acid-induced protein ARG7-like [Glycine max]
Vr77281_c0_seq1	583	709	19.03	44	1.44	3.72	2.8E-128	8.1E-127	XP_003538774.1	nitrate transporter 1.5-like [Glycine max]
Vr14500_c0_seq1	1197	648	8.47	60	0.96	3.14	1.3E-101	3.5E-100	XP_003553615.1	uncharacterized protein LOC100785434 [Glycine max]
Vr30229_c0_seq3	2272	624	4.30	50	0.42	3.35	9.8E-104	2.5E-102	XP_003525884.1	transcription factor bHLH25-like [Glycine max]
Vr59002_c0_seq1	685	600	13.70	56	1.56	3.13	7.13E-94	1.78E-92	ACU17538.1	unknown [Glycine max]
Vr38010_c0_seq4	2806	503	2.80	45	0.31	3.19	3.75E-80	8.71E-79	XP_003528365.1	pleiotropic drug resistance protein 1-like [Glycine max]
Vr38147_c0_seq1	1608	191529	1863.56	39055	464.80	2.00	0	0	NP_001237601.1	cationic peroxidase 2 precursor [Glycine max]
Vr40878_c0_seq1	1582	18917	187.09	2801	33.88	2.47	0	0	AAC49758.1	gibberellin 20-oxidase [Phaseolus vulgaris]
Vr32702_c0_seq1	1886	14859	123.27	3015	30.59	2.01	0	0	XP_003519380.1	uncharacterized acetyltransferase At3g50280 [Glycine max]
Vr34855_c0_seq2	1659	14639	138.06	1577	18.19	2.92	0	0	XP_003550831.1	zinc finger Ran-binding domain-containing protein 2-like [Glycine max]
Vr13816_c0_seq1	3608	13084	56.74	2548	13.51	2.07	0	0	XP_003555774.1	transcription factor PIF3-like [Glycine max]
Vr39199_c0_seq1	794	12881	253.82	2396	57.75	2.14	0	0	AFU81504.1	LEA5-2.1, partial, late embryogenesis abundant [Vigna unguiculata]
Vr34116_c0_seq7	2268	9555	65.92	1469	12.40	2.41	0	0	XP_003547999.1	uncharacterized membrane protein YOL092W-like [Glycine max]
Vr43985_c0_seq1	1876	9228	76.96	1049	10.70	2.85	0	0	XP_003549204.1	putative nuclease HARBI1-like [Glycine max]
Vr28990_c0_seq3	3586	9216	40.21	1776	9.48	2.08	0	0	XP_003526176.1	chitin-inducible gibberellin-responsive protein 1-like [Glycine max]
Vr41680_c0_seq1	1842	8813	74.86	961	9.98	2.91	0	0	XP_003542412.1	cytochrome P450 94A2-like [Glycine max]
Vr33063_c0_seq2	4741	8706	28.73	1443	5.82	2.30	0	0	XP_004512150.1	histidine kinase 1-like isoform X2 [Cicer arietinum]
Vr13792_c0_seq1	2255	8402	58.30	1317	11.18	2.38	0	0	XP_003553732.1	uncharacterized protein LOC100811813 [Glycine max]
Vr41811_c0_seq1	1779	8207	72.18	1647	17.72	2.03	0	0	XP_003553527.1	auxin-induced protein 5NG4-like [Glycine max]
Vr33482_c1_seq4	2171	7035	50.70	1332	11.74	2.11	0	0	NP_001242710.1	uncharacterized protein LOC100805837 [Glycine max]
