# Supplementary material for: De Novo Characterization of the Mung Bean Transcriptome and Transcriptomic Analysis of Adventitious Rooting in Seedlings Using RNA-Seq
Source: PLoS One. 2015 Jul 15;10(7):e0132969. doi: 10.1371/journal.pone.0132969 (PMC4503682; doi:10.1371/journal.pone.0132969)
Supplement: S8 Table — (PDF) [file pone.0132969.s012.pdf]

S8 Table. Genes selected for q-PCR validation

Gene ID	Functional description	Swissprot ID	Bitscore	E-value	RPKM		
					Con	Wat6	Wat24
comp37254	Auxin response factor 2, ARF2	sp|Q94JM3	952	0	89.852	37.954	50.760
comp35374	Auxin response factor 18, ARF18	sp|Q653H7	816	0	26.952	16.698	14.999
comp35831	Auxin response factor 1,ARF1	sp|Q8L7G0	885	0	29.128	15.349	16.768
comp29470	Auxin response factor 3,ARF3	sp|O23661	450	1E-142	32.138	10.622	12.376
comp13756	Auxin response factor 6,ARF6	sp|Q9ZTX8	376	2E-114	40.028	15.658	13.549
comp36957	Auxin response factor 19,ARF19	sp|Q8RYC8	734	0	57.040	24.402	29.694
comp24483	Auxin response factor 8,ARF8	sp|Q9FGV1	278	3E-82	13.053	8.386	8.696
comp23260	Auxin transporter-like protein 4	sp|Q8L884	851	0	159.520	73.881	49.185
comp34322	LOB domain-containing protein 41,LBD41	sp|Q9M886	185	3E-53	55.243	121.677	134.119
comp39013	LOB domain-containing protein 21,LBD21	sp|Q9SRL8	188	1E-54	94.987	84.197	46.743
comp40540	LOB domain-containing protein 29,LBD29	sp|Q9M2J7	186	3E-54	3.714	26.745	12.554
comp38129	Sodium/hydrogen exchanger 2, NHX2	sp|Q56XP4	333	5E-103	381.908	429.401	472.443
comp40252	NAC domain-containing protein 72 NAC72	sp|Q93VY3	350	2E-114	73.946	21.275	61.914
comp44893	NAC domain-containing protein ,NAC1	sp|Q84TE6	339	5E-112	24.715	17.499	19.606
comp56348	NAC transcription factor , NAC2	sp|Q8GY42	303	3E-97	7.724	1.993	6.702
comp34452	NAC transcription factor NAM-B2, NAMB2	sp|A0SPJ6	188	9E-53	105.389	35.786	100.297
comp32636	Auxin-responsive protein IAA9, IAA9	sp|Q38827	404	8E-132	167.645	180.603	152.081
comp13386	Auxin-responsive protein IAA26, IAA26	sp|Q8LAL2	227	9E-67	95.846	129.343	104.800
comp32189	Auxin-responsive protein IAA14, IAA14	sp|Q38832	337	7E-111	630.691	551.345	220.222
comp13709	Auxin-responsive protein IAA8, IAA8	sp|Q38826	207	1E-58	112.586	79.588	82.916
comp38087	Alcohol dehydrogenase 1, ADH1	sp|P13603	618	0	7170.197	7091.752	3678.503
comp13399	Alcohol dehydrogenase 1, ADH1	sp|P13603	307	5E-101	52.076	202.286	151.123
comp33063	Histidine kinase 1, AHK1	sp|Q9SXL4	847	0	28.731	1.089	5.825
comp33358	Histidine kinase 2, AHK2	sp|Q9C5U2	1275	0	20.795	12.318	11.801
comp40153	Histidine kinase 3, AHK3	sp|Q9C5U1	1351	0	49.501	35.556	34.619
comp38180	Dehydration-responsive protein RD22, RD22	sp|Q08298	310	2E-99	1249.984	356.157	738.560
comp34639	Auxin-induced protein 22E, AUX22E	sp|O24543	379	8E-129	52.651	50.996	26.642
comp31234	Auxin-induced protein 22B ,AUX22B	sp|P32294	279	3E-88	58.082	58.968	37.863
comp39198	Auxin-induced protein 22C ,AUX22C	sp|O24541	268	5E-89	6.366	43.768	4.778
comp47043	Auxin-induced protein 15A, AUX15A	sp|P33081	85.9	8E-20	1.261	39.716	2.638
comp22610	Cationic peroxidase 1, PER1	sp|P22195	324	2E-107	0.193	139.508	372.845
comp21159	Auxin efflux carrier component 1,PINI	sp|Q9C6B8	464	2E-156	0.317	15.078	10.054
comp39799	Protein LHY ,LHY	sp|Q6R0H1	122	2E-28	249.382	5.232	112.908
comp40489	Protein LHY,LHY	sp|Q6R0H1	118	5E-28	223.864	4.108	94.885
comp31128	Cationic peroxidase 2 , PER2	sp|P22196	485	1E-168	4.195	169.179	226.783
comp36822	Quinone oxidoreductase-like protein	sp|Q9ZUC1	378	1E-124	7.085	40.927	4.776
comp39707	Peptidyl-prolyl cis-trans isomerase CYP20,CYP20	sp|Q9ASS6	289	9E-94	58.173	62.356	58.411
comp38164	Eukaryotic translation initiation factor 5A,e IF5A	sp|Q9AXJ4	313	4E-105	606.853	602.711	602.154
comp31451	Actin-related protein 4, ACTIN	sp|Q84M92	712	0	18.727	19.322	20.725

Primers for q-PCR verification

comp39707 F: 5' TCCCCAAACAGCCGAAAA 3' 59.5
comp39707 R: 5' CCCCTTGAATCATGAAATCCTT 3' 59.9 107bp
comp38164 F: 5' ACCTTAGGCTTCCAACCGAT 3' 58.0
comp38164 R: 5' CTCCCATTGCAGACATAACAGA 3' 57.8 99bp
comp31451 F: 5' CGTGTTTCCTTCTGTTGTTGG 3' 58.1
comp31451 R: 5' CCTCTTTCCCTTAGCCTTGTC 3' 57.7 142bp
comp37254 F：5' ATCTCTCATCCGCAACACTCCT 3' 60.3
comp37254 R：5' TTCACCCCCACTTCTCTTACCTC 3' 61.4 123bp
comp35374 F：5' ACCTTGTCGCTGTCTTCGTGG 3' 62.4
comp35374 R：5' GTGGCGGCTGTTAAATTCCTC 3' 61.4 110bp
comp35831 F：5' CCGCAGCCAACACAAAGAAAC 3' 63
comp35831 R：5' GGTGATGGATAGAGGCCACGTC 3' 63.1 155bp
comp29470 F：5' CTTGTTCTAAGTGCCCTTGTGG 3' 59.3
comp29470 R: 5' GCTTGCGTTTGCGTTTCCT 3' 61.3 175bp
comp13756 F：5' AAGGTTGGCTGGGATGAAT 3' 58.7
comp13756 R: 5' TAAGGGGAAATGGAGATGGAT 3' 57.1 109bp
comp36957 F: 5' GCTGGCTATTCTGCTGTACCTG 3' 59.5
comp36957 R: 5' TCCCTTACTATTGGAGTCGGC 3' 58.7 96bp
comp24483 F: 5' TTGAGGGAAAGTTGGAAGACC 3' 58.3
comp24483 R: 5' CCTGAACCTAGAGCAAGGGATT 3' 59.5 196bp
comp23260 F: 5' ACTCCAACGCCTTCTCTCTCCTC 3' 63.1
comp23260 R: 5' ATCCCAATCACCTTCTCCCACA 3' 62.9 136bp
comp34322 F: 5' CAATGGATAAGTTGCCCTCAC 3' 57.2
comp34322 R: 5' ACCGCTGGTTGGAGATGG 3' 58.5 119bp
comp39013 F: 5' CAAGGCTCAGAGACCCCG 3' 58.4
comp39013 R: 5' TAGCAGCAGCGTGACATCG 3' 58.8 126bp
comp40540 F: 5' ATCTCAGGGAACAGGCAGGTC 3' 60.4
comp40540 R: 5' TCCGTCTGGAACCAACTCTGA 3' 60.2 121bp
comp38129 F: 5' CTGAACTGCGAACCAATGC 3' 57.1
comp38129 R: 5' TTCTTGCGTTTAGGAGGTGG 3' 58.0 136bp
comp40252 F: 5' AGACCCTCTTGCCCAGTTG 3' 57.1
comp40252 R: 5' CCGAAAATCGCCTTGCTT 3' 58.7 180bp
comp44893 F: 5' AGGGTTCAGGTTCCATCCAA 3' 59.0
comp44893 R: 5' GAATATCCCAAGGTTCGCACT 3' 58.5 125bp
comp56348 F: 5' ATTCAACCTCAAAACCCCCT 3' 57.6
comp56348 R: 5' CACAACACCCAATCATCAAGC 3' 58.5 76bp
comp34452 F: 5' CCGACGAGGAATTGGTTGTG 3' 61.0
comp34452 R: 5' GAGCGGTTGCCGTTAGGATA 3' 60.4 181bp
comp32636 F: 5' GACTGTAGCGTCTGGTTCTTCG 3' 59.3
comp32636 R: 5' CCTCAACTCCGTAGCCTTCAAA 3' 61.3 181bp
comp13386 F: 5' ACCCCACCAAGAGAAAGGAAG 3' 59.8
comp13386 R: 5' CCCACTAAGCGTGCAAATAAAA 3' 60.2 128bp
comp32189 F: 5' GTGGAAACACCAAGGGCTACTG 3' 61.2
comp32189 R: 5' CCAACCAACCACTTGAGCCT 3' 59.7 171bp

comp13709 F: 5' TGGGCATAGGAGAGGAGGA 3' 57.5
comp13709 R: 5' GAGAAAGACGGAACGGAGC 3' 57.0 163bp
comp38087 F: 5' AGAATCCGCTGTTTCCTCGT 3' 58.8
comp38087 R: 5' ACAGGTTGCTCTCTTCCGATT 3' 58.1 162bp
comp13399 F: 5' AGGCTGGTGAAGTGCGTTT 3' 57.9
comp13399 R: 5' TATGCGAGGAAACAATGGAGT 3' 57.4 101bp
comp33063 F: 5' ACAAGAGCACCACCCCAAAA 3' 60.2
comp33063 R: 5' TGTGTAAATCTTCGTGAAATGCC 3' 59.7 194bp
comp33358 F: 5' GCATCAGGGGGGGTGTTTG 3' 62.9
comp33358 R: 5' GCCTCAGCACGGCTTTTCAG 3' 63.2 125bp
comp40153 F: 5' CAAATCGTCTGGGGGTTATCC 3' 60.8
comp40153 R: 5' GAAGCAGTTTCTCCACCAATGAC 3' 60.5 144bp
comp38180 F: 5' CCCGAGTCAGAGGAGGCTAA 3' 59.3
comp38180 R: 5' CCCCGTTTCTTTGTCAACTTCT 3' 60.2 177bp
comp34639 F: 5' AACTCTGATTCTTCGGACTCCACAAC 3' 64.3
comp34639 R: 5' TCGCCCTGCTCCACCTTTTT 3' 64.3 143bp
comp31234 F: 5' GCTCCCAGGAACAGAAGAAA 3' 56.8
comp31234 R: 5' ATAAATCCCAGCACCCTCAT 3' 56.1 199bp
comp39198 F: 5' TGGCCTCCGGTTTGCTCCTA 3' 65.1
comp39198 R: 5' CCGCATCCTTCAACGCTTCG 3' 66.1 193bp
comp47043 F: 5' TCAAGTTTTGGCAAGAAGCA 3' 57.0
comp47043 R: 5' GTTCTCCCCAACATAGACAGC 3' 56.2 90bp
comp22610 F: 5' CAAGAATCTCATCAACCAAAAGGG 3' 62.1
comp22610 R: 5' GGGCTAAGGTTTCCCATTTTG 3' 60.5 156bp
comp21159 F: 5' ATGCGGAGATTTACTCGCTTCA 3' 61.7
comp21159 R: 5' CCCCCCCAAAACTACTCTGC 3' 61.0 132bp
comp39799 F: 5' AAAAGGAAGAGCCCAACAAAG 3 '58.2
comp39799 R: 5' CTGGAGAATAGCGCCTGAAA 3' 58.2 145bp
comp40489 F: 5' GGACCTGCTTCATCTGTATCACTG 3' 60.5
comp40489 R: 5' TGTTTGTGGTCTCCGTCATCTAA 3' 59.8 164bp
comp31128 F: 5' CCTAGACACGGGTAGTCAAAGAGC 3' 61.6
comp31128 R: 5' TCATCGTTCCAGAGGGCTTG 3' 60.7 102bp
